# Supplementary material for: Highly ionic-dispersed oxygen electrode for reversible proton ceramic electrochemical cells
Source: Nat Commun. 2026 Mar 14;17:3989. doi: 10.1038/s41467-026-70738-z (PMC13136414; doi:10.1038/s41467-026-70738-z)
Supplement: Supplementary file 1 — Supplementary Information [file 41467_2026_70738_MOESM1_ESM.pdf]

Supplementary Information

Highly ionic-dispersion oxygen electrode for proton ceramic electrochemical cells

Xiaoyu Wang[1,2], Zhaohui Cai[2], Zeping Chen[1,2], Donliang Liu[1,2], Wanqing Chen[1,2], Jianqiu Zhu[3], Wenhuai Li[1], Xixi Wang[4], Linjuan Zhan[3], Wei Wang[1,2], Chuan Zhou[1,2], Wei Zhou[1,2]*, Zongping Shao[5]**

[1]State Key Laboratory of Materials-Oriented Chemical Engineering, College of Chemical Engineering, Nanjing Tech University, Nanjing 211816, China.

[2]Suzhou Laboratory, Suzhou 215000, China

[3]Key Laboratory of Interfacial Physics and Technology, Shanghai Institute of Applied Physics, Chinese Academy of Sciences, Shanghai 201800, China

[4]School of Environmental Science and Engineering, Nanjing Tech University, Nanjing 211816, China

[5]Curtin Centre for Advanced Energy Materials and Technologies (CAEMT), Western Australian School of Mines (WASM), Curtin University, Perth, Western Australia 6102, Australia

*Corresponding author E-mail: zhouc@szlab.ac.cn (C. Zhou), zhouwei1982@njtech.edu.cn (W. Zhou), Zongping.Shao@curtin.edu.au (Z. Shao).

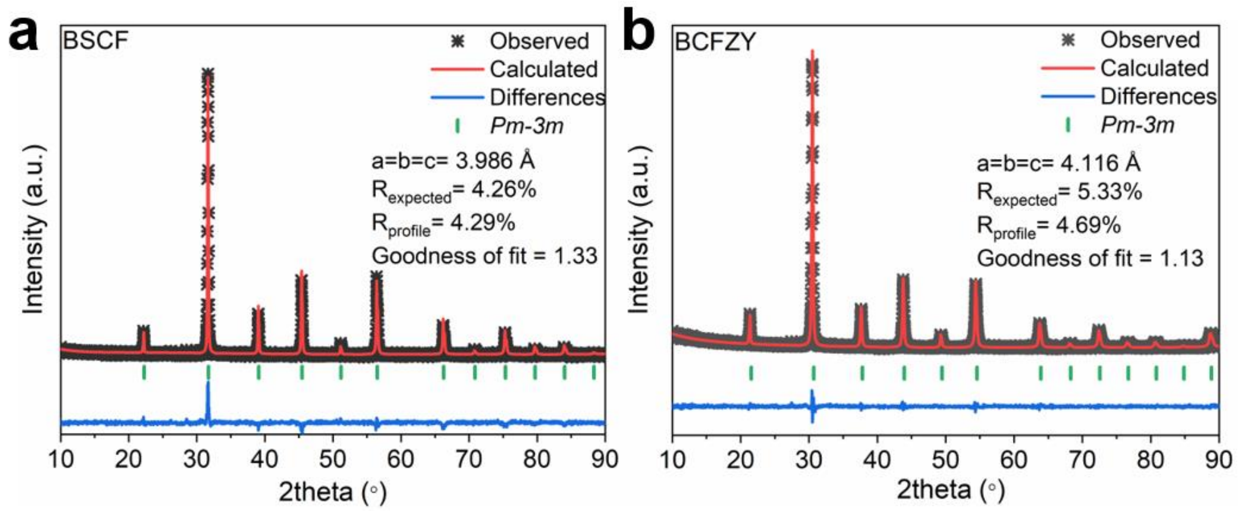

Supplementary Fig. 1.

Rietveld Refinement XRD profiles of the prepared (a) BSCF and (b) BCFZY.

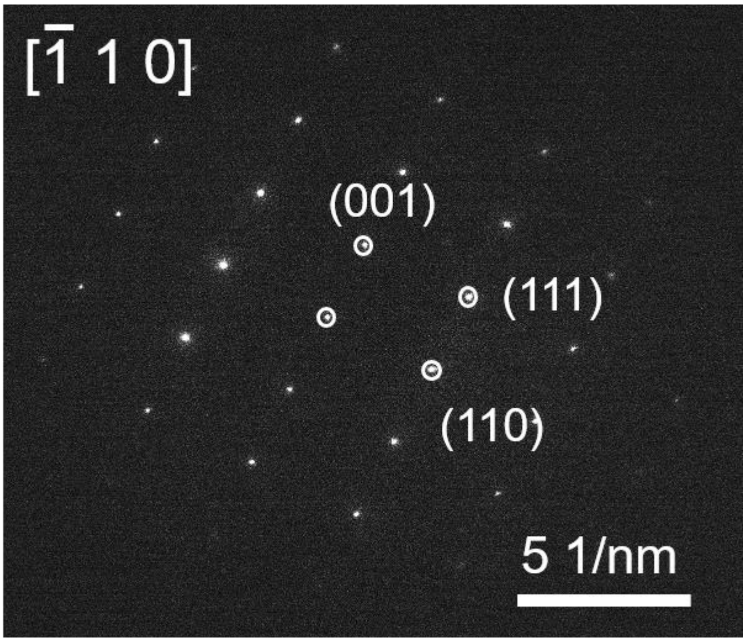

Supplementary Fig. 2.

SEAD pattern of the [$\bar{1}$10] zone axis of the BCZTZICM.

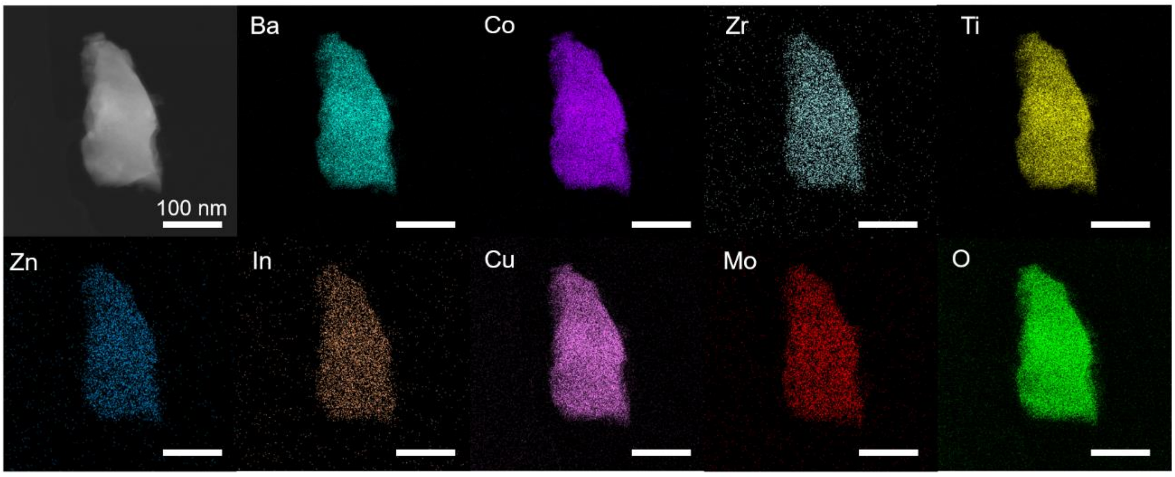

Supplementary Fig. 3.

EDS-mapping result of BCZTZICM.

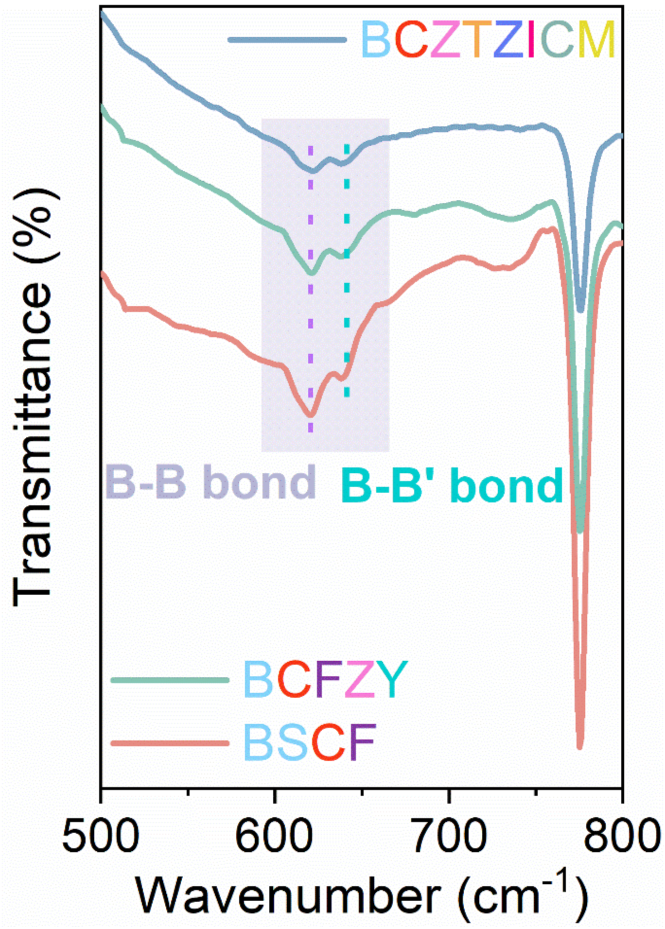

Supplementary Fig. 4.

FT-IR spectra of BCZTZICM, BSCF and BCFZY samples.

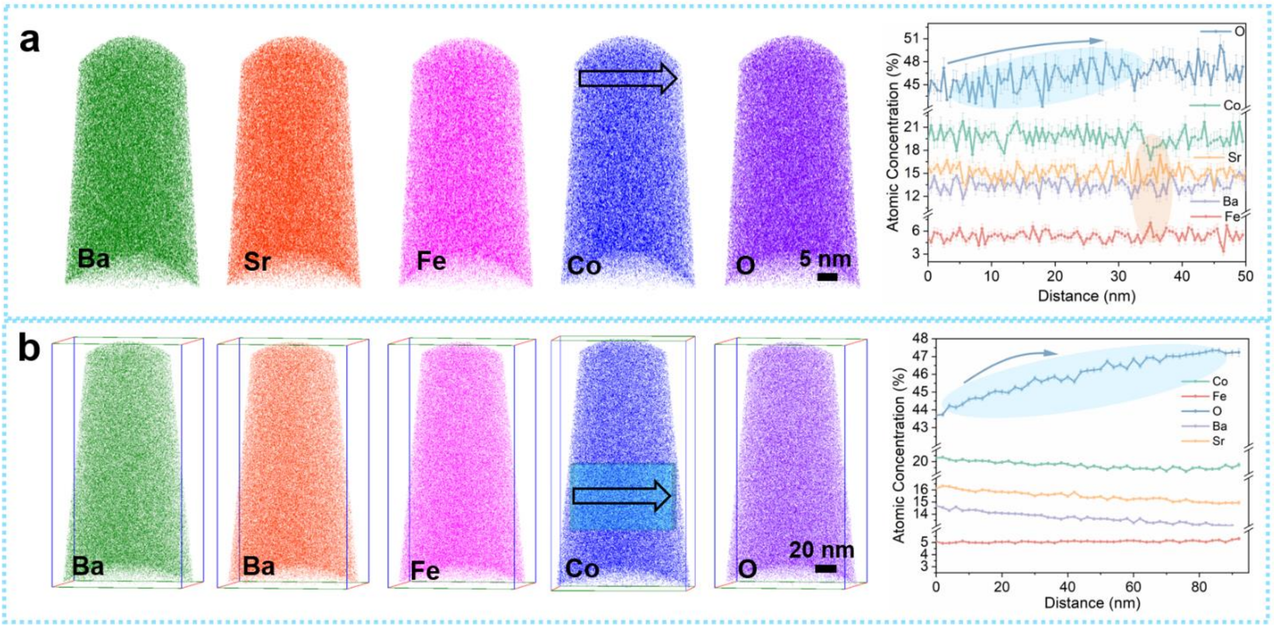

Supplementary Fig. 5.

3D reconstruction of the APT data showing mapping of the BSCF sample with Ba, Sr, Co, Fe, and O ionic distributions. Integrated line profiles show chemical composition at the (a) apex and (b) midsection regions of the analyzed needle.

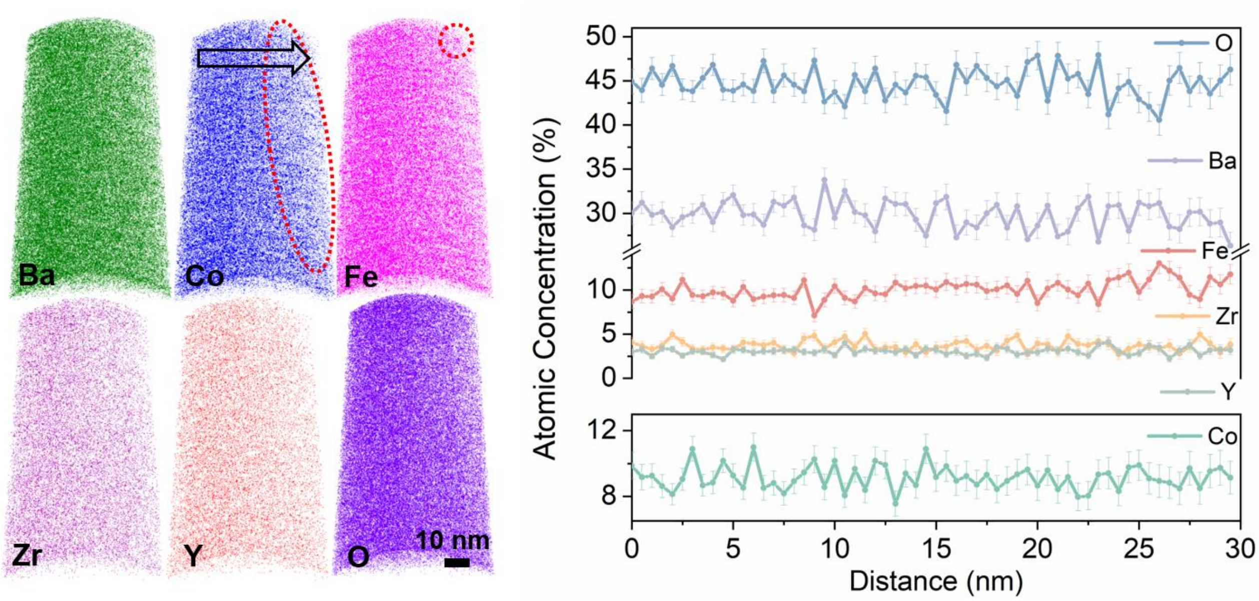

Supplementary Fig. 6.

3D reconstruction of the APT data showing mapping of the BCFZY sample with Ba, Co, Fe, Zr, Y, and O ionic distributions. Integrated line profiles show chemical composition at the apex regions of the analyzed needle.

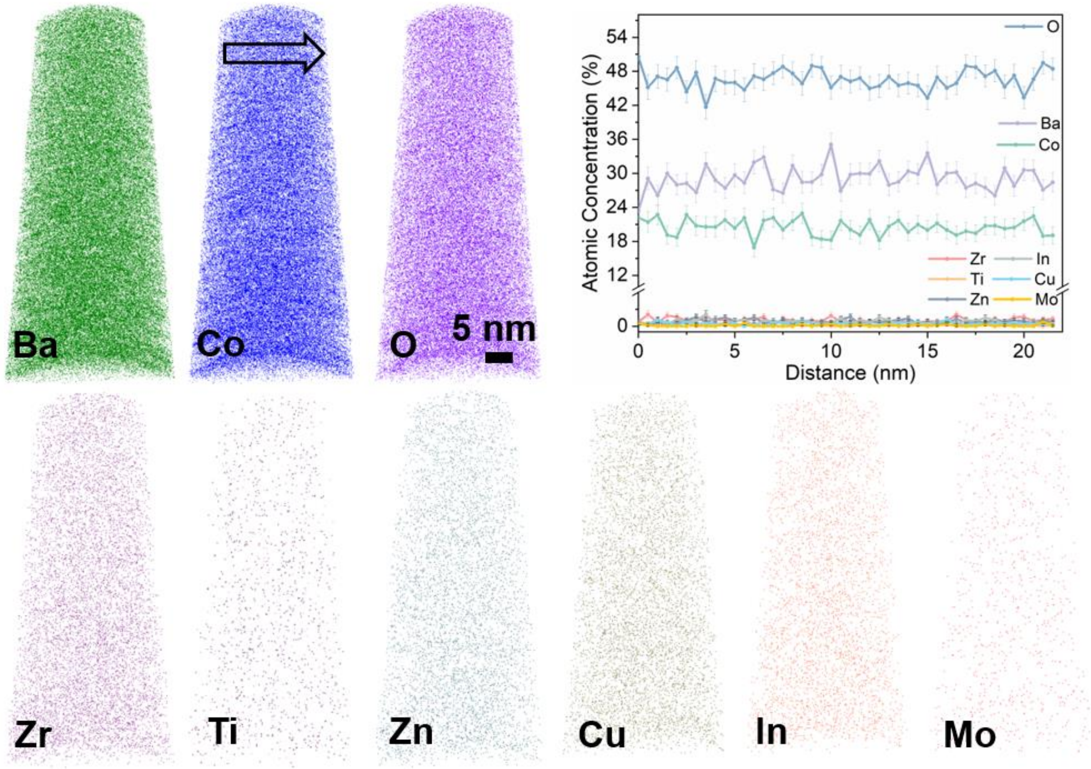

Supplementary Fig. 7.

3D reconstruction of the APT data showing mapping of the BCZTZICM sample with elements distributions. Integrated line profiles show chemical composition alongside the y-axis of the analyzed needle.

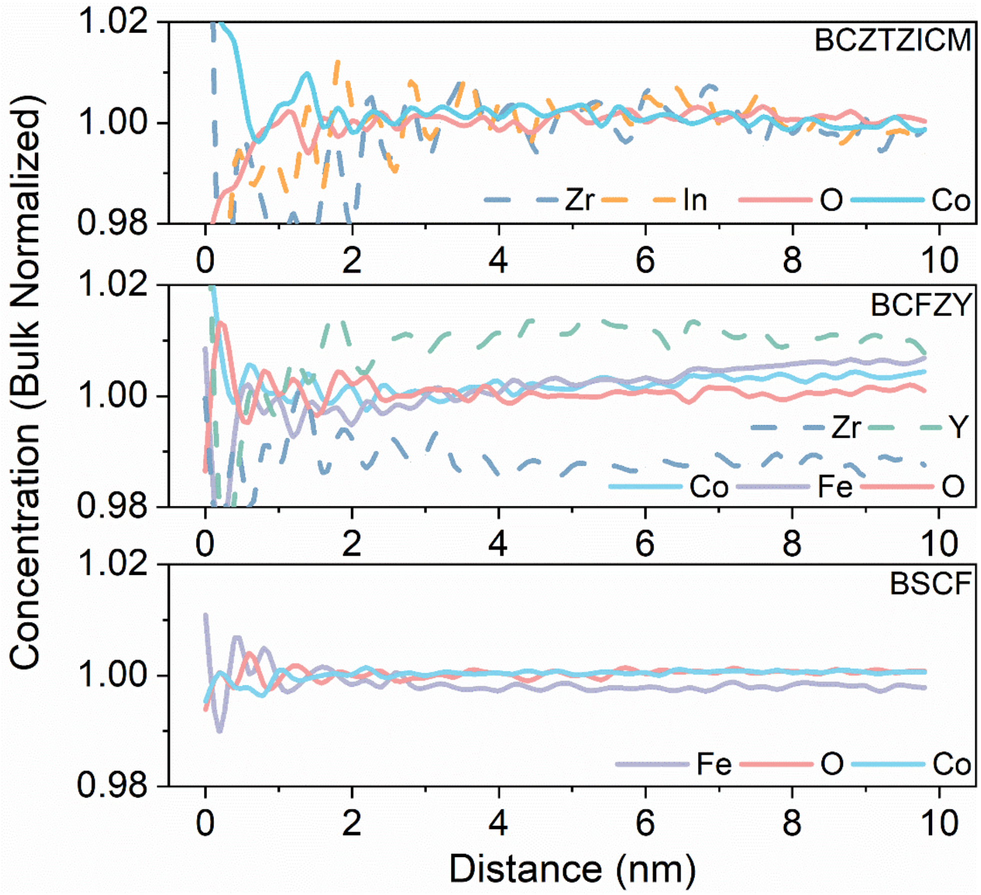

Supplementary Fig. 8.

Radial distribution function (RDF) analysis of neighboring ions around Co centers within a 10 nm range, derived from APT data.

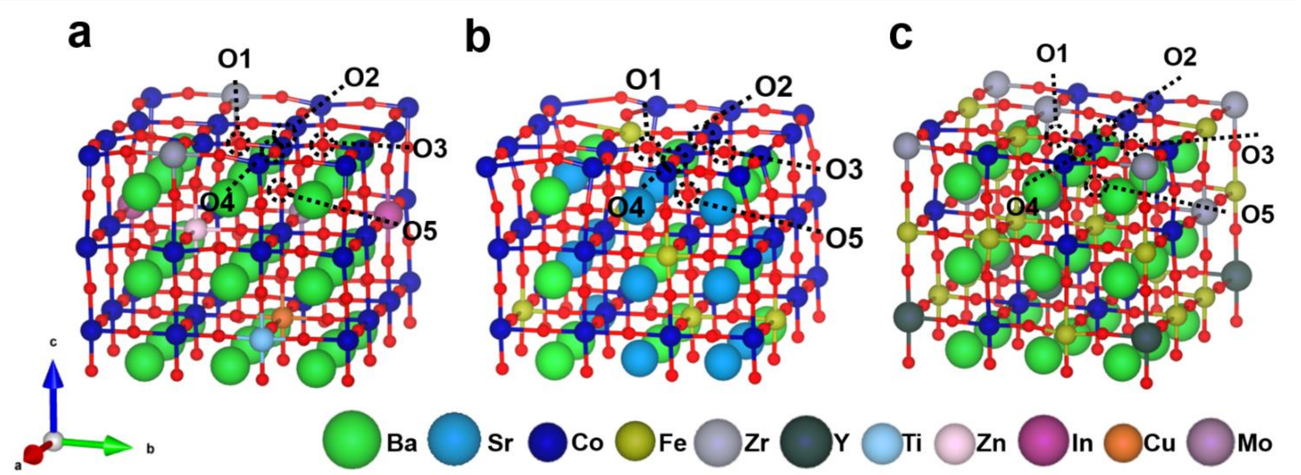

Supplementary Fig. 9.

Optimized structure of stoichiometric pristine of (a) BCZTZICM, (b) BSCF, and (c) BCFZY, and unit cell oxygen sites in perovskite are labeled as calculated sites.

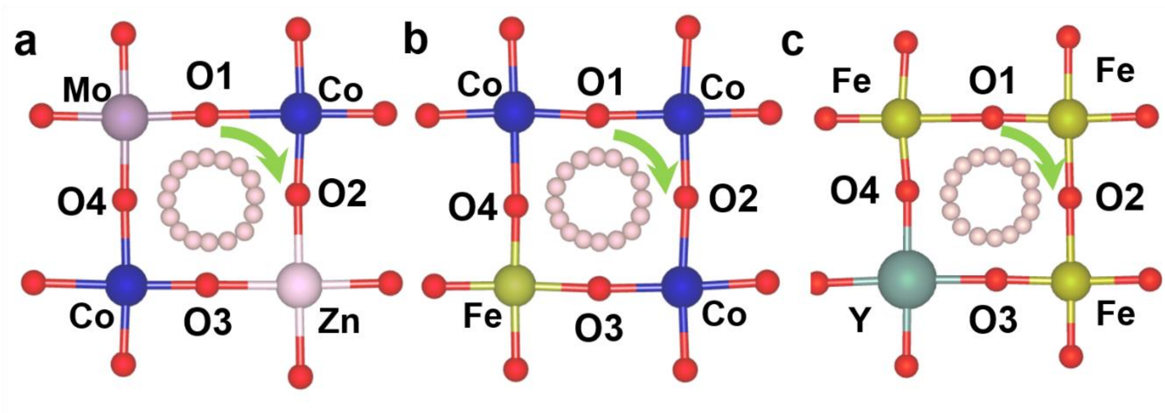

Supplementary Fig. 10.

Proton diffusion paths of the perovskites (a) BCZTZICM, (b) BSCF, and (c) BCFZY, with the direction of the proton migration indicated on the inside.

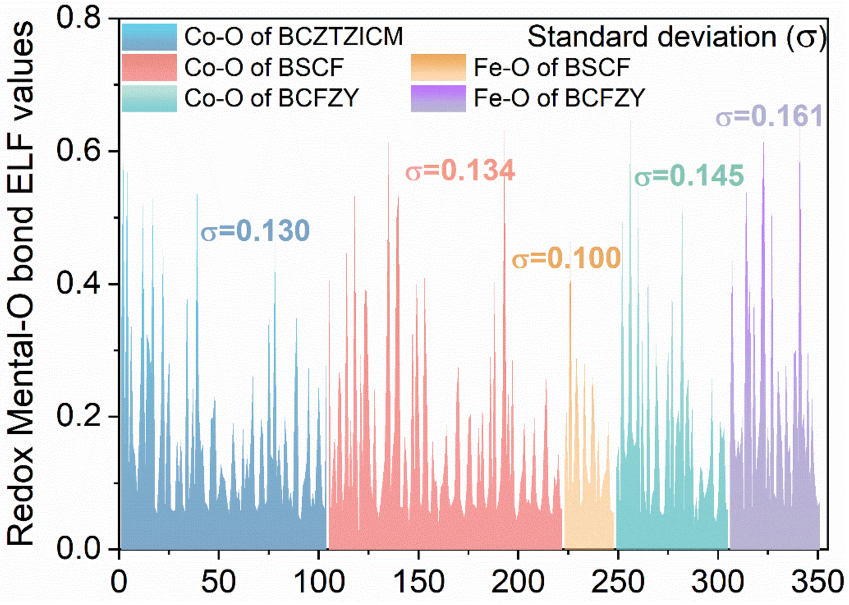

Supplementary Fig. 11.

ELF values of redox metal-oxygen bonds of BSCF, BCFZY, and multi-element micro-doping BCZTZICM sample.

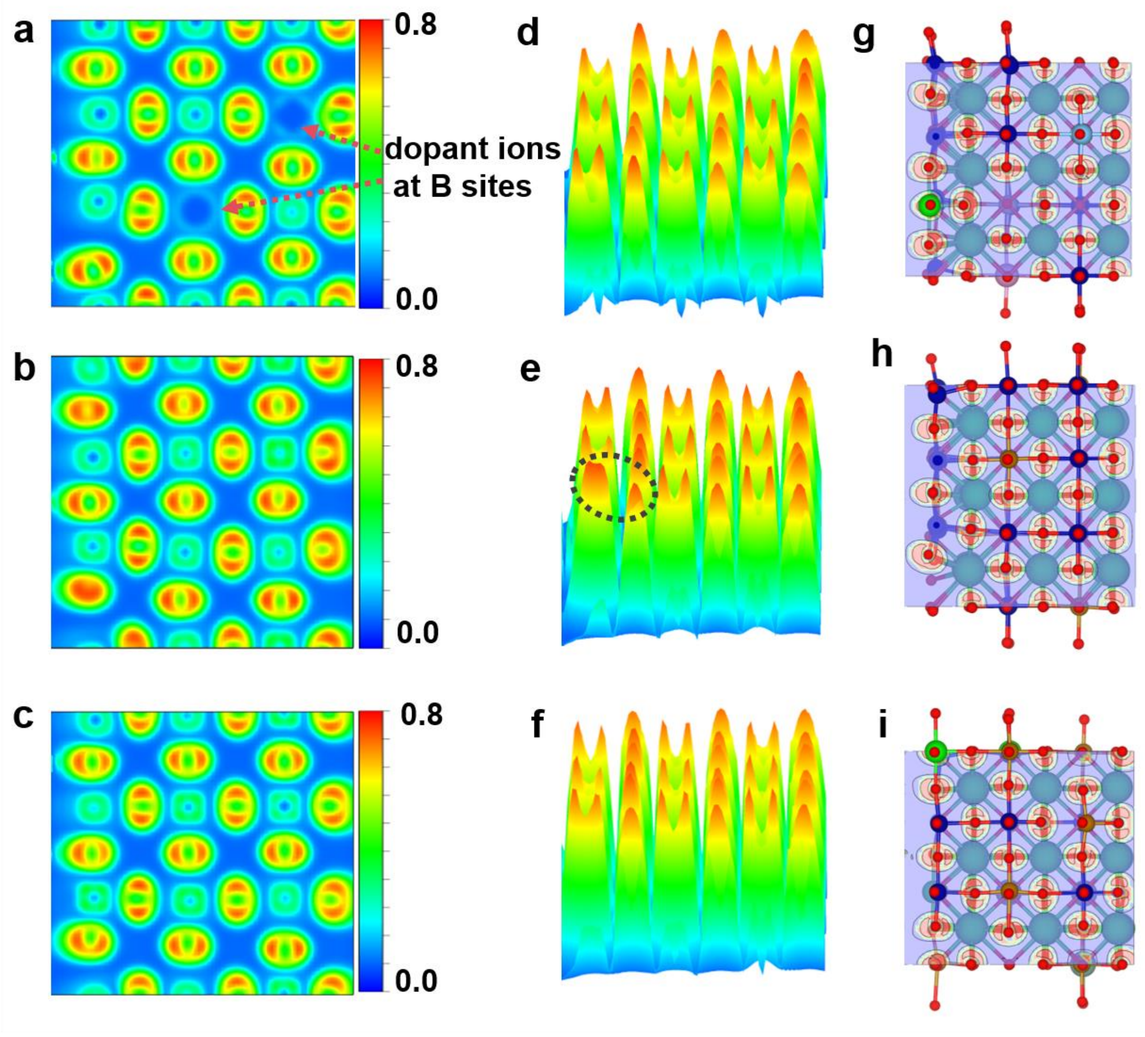

Supplementary Fig. 12.

ELF visualizations diagram along the (100) plane of (a) BCZTZICM, (b) BSCF, and (c) BCFZY, where the color bars indicate ELF values. Bird's-eye view of ELF distributions for (d) BCZTZICM, (e) BSCF, and (f) BCFZY. Electron density isosurfaces for (g) BCZTZICM, (h) BSCF, and (i) BCFZY, respectively.

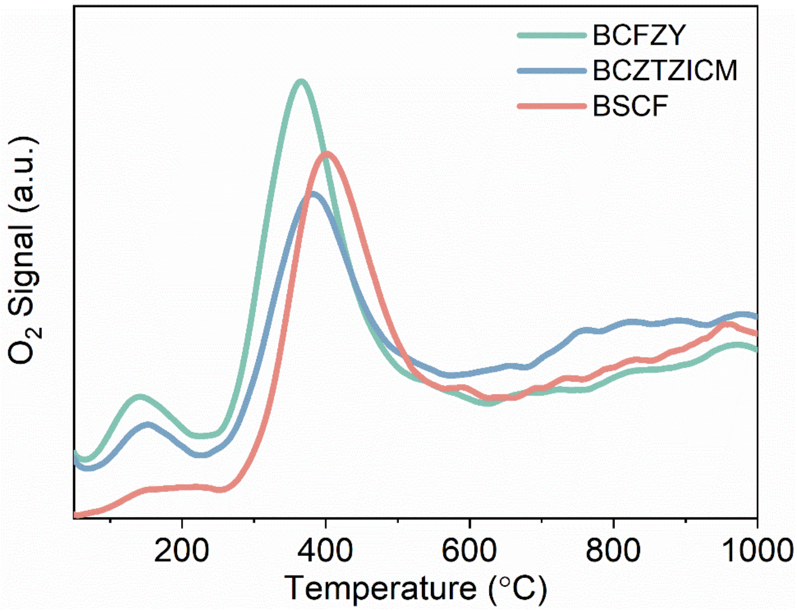

Supplementary Fig. 13.

O_2-TPD profiles from 25 °C to 1000 °C.

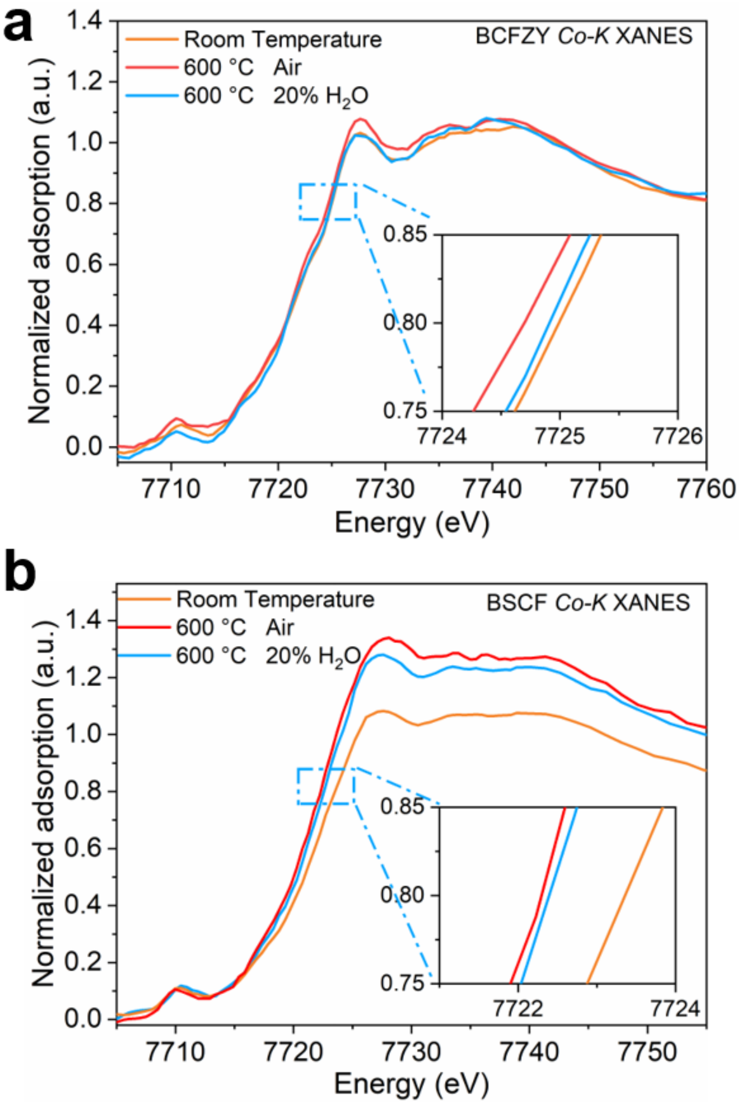

Supplementary Fig. 14.

Co K-edge XANES data of (a) BCFZY and (b) BSCF at different temperatures and treatments.

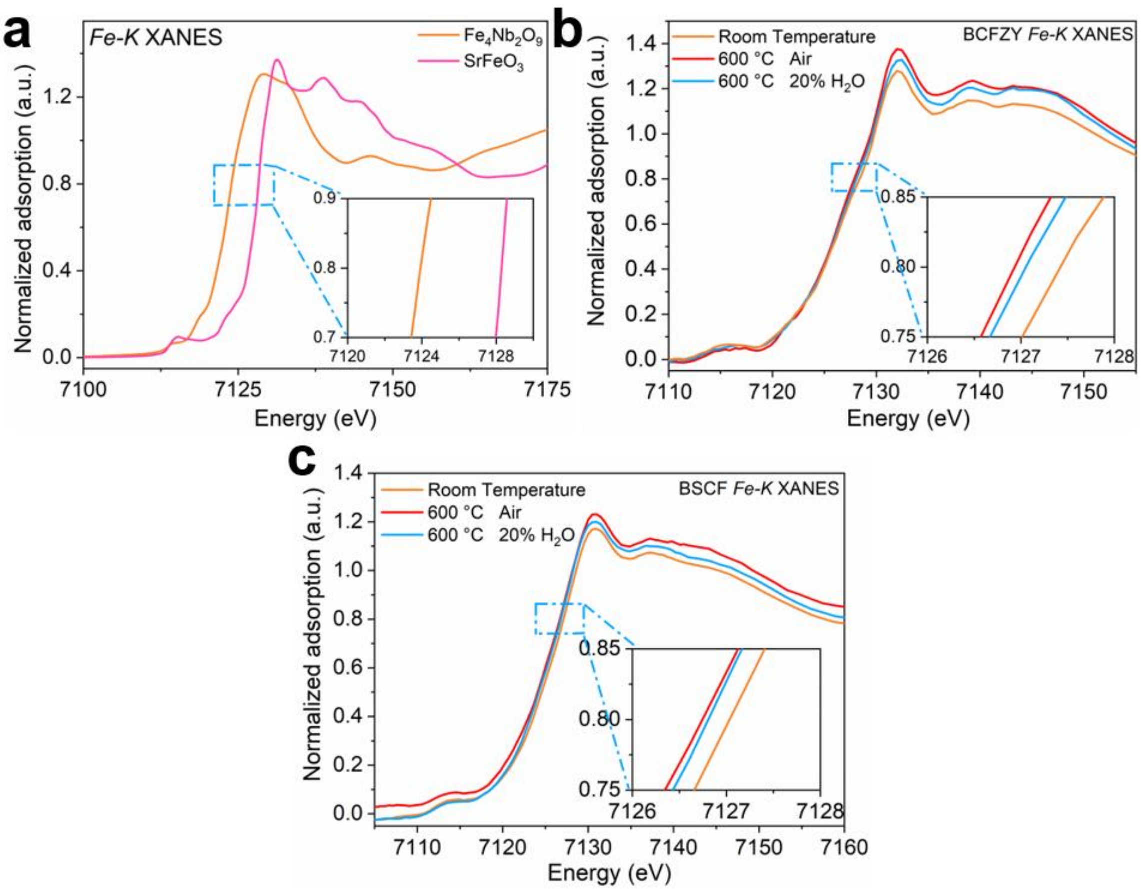

Supplementary Fig. 15.

(a) XANES data for Fe standard samples. Fe K-edge XANES data of (b) BCFZY and (c) BSCF at different temperatures and treatments.

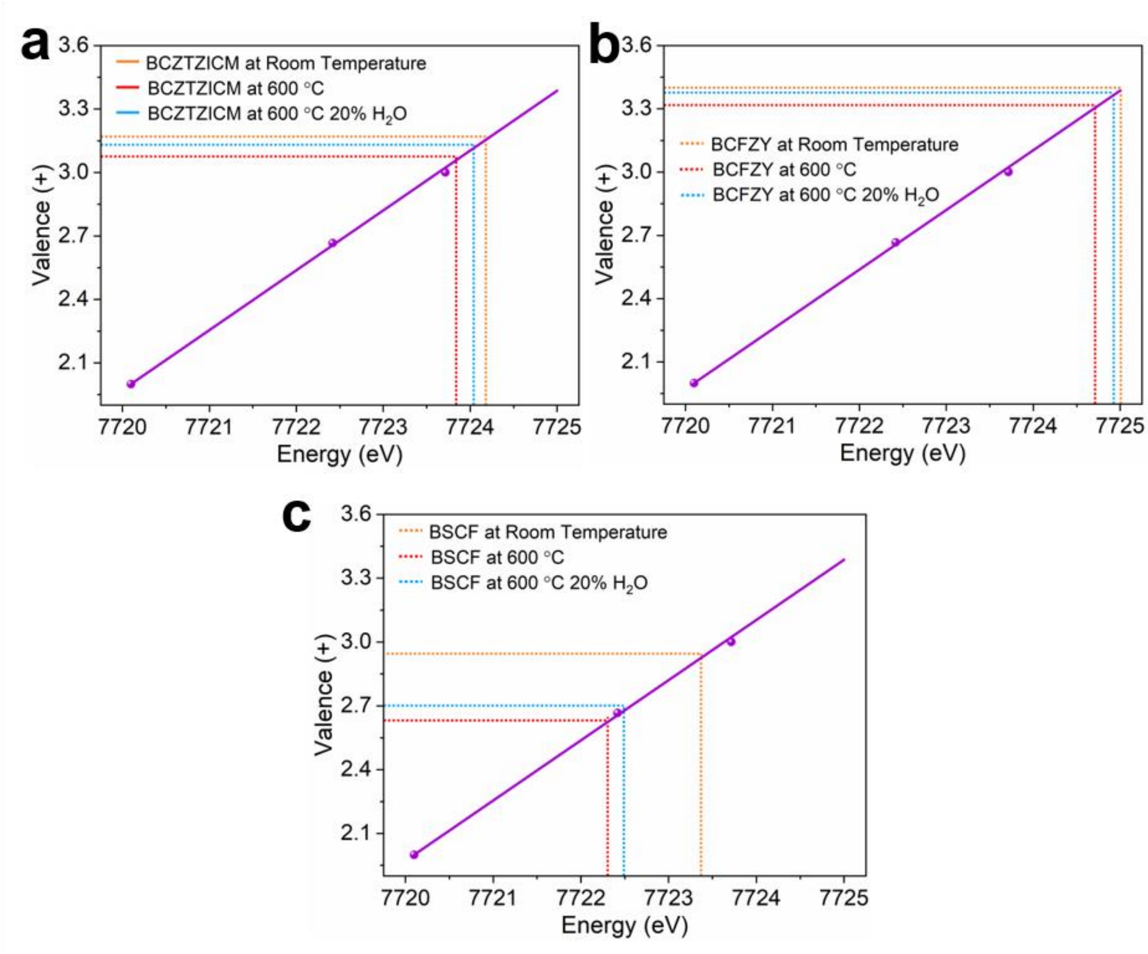

Supplementary Fig. 16.

Co ions average valence calculated from the absorption edge shift in XANES data of (a) BCZTZICM, (b) BCFZY, and (c) BSCF at different temperatures and treatments.

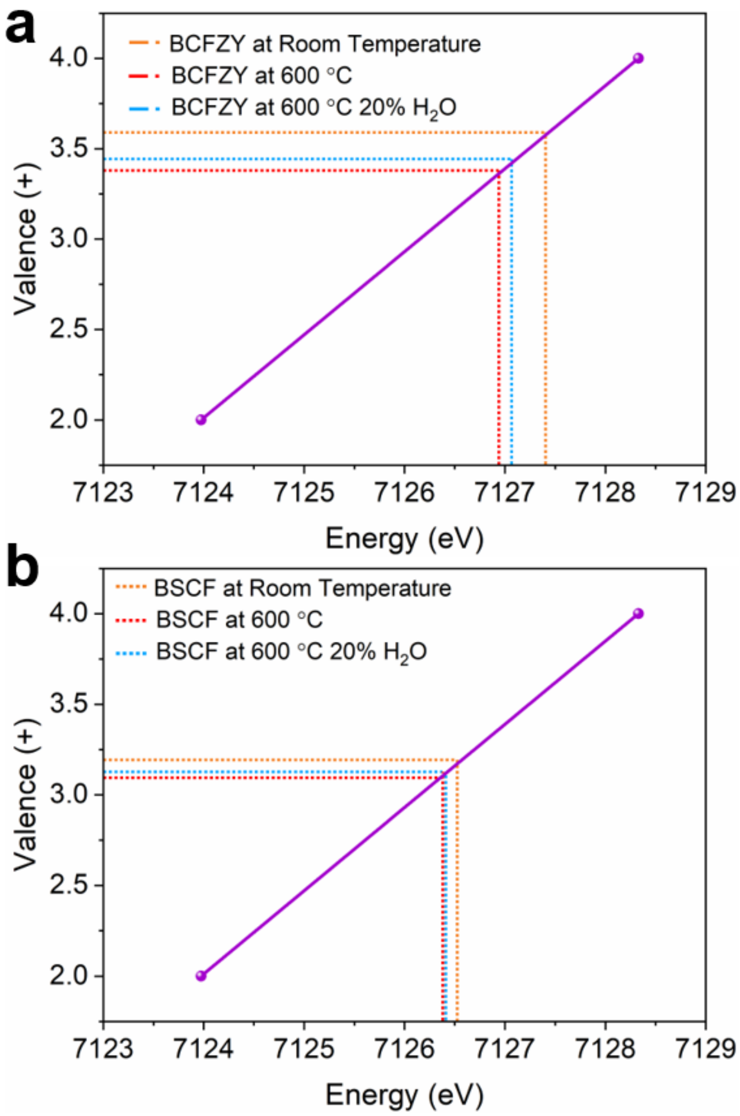

Supplementary Fig. 17.

Fe average valence calculated from the absorption edge shift in XANES data of (a) BCFZY and (b) BSCF at different temperatures and treatments.

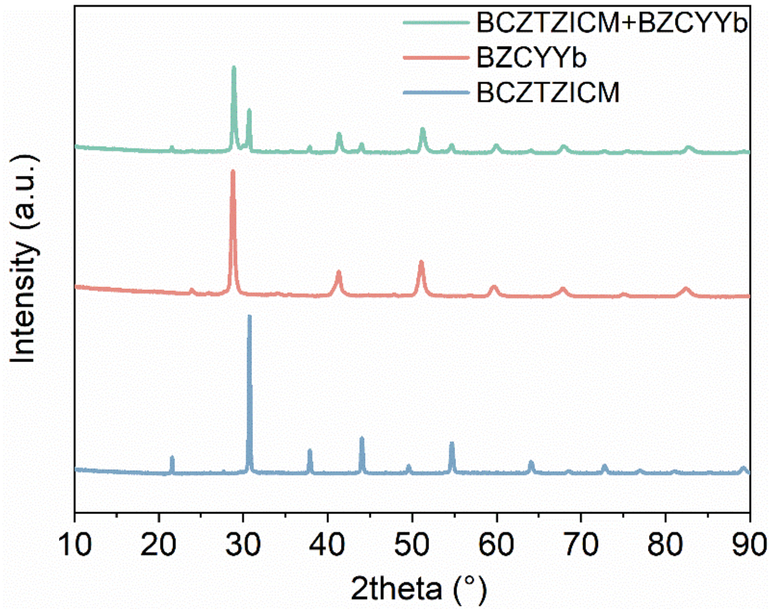

Supplementary Fig. 18.

XRD patterns of BCZTZICM electrode, BZCYYb electrolyte, and their composites with a mass ratio of 1:1 after calcination at 1000 °C for 2 h in air.

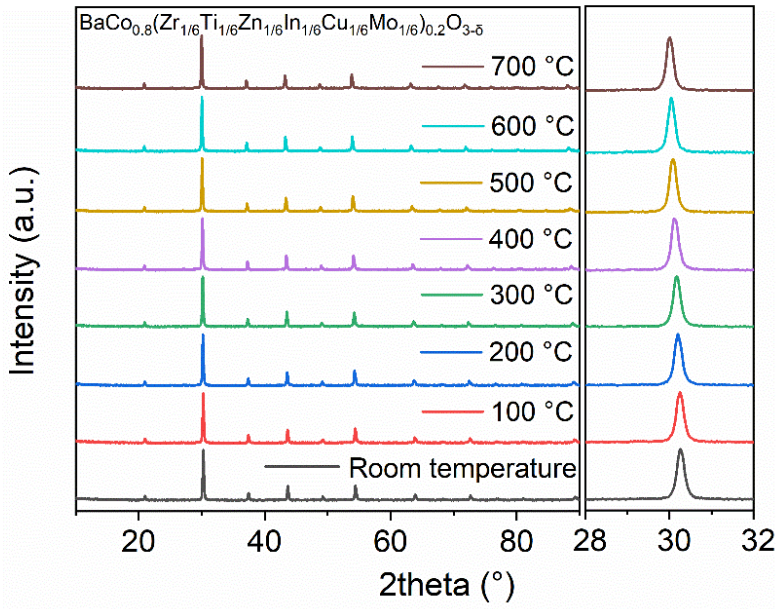

Supplementary Fig. 19.

High temperature XRD patterns of BCZTZICM powder, and magnified XRD patterns of the selected 2theta range of 28-32°.

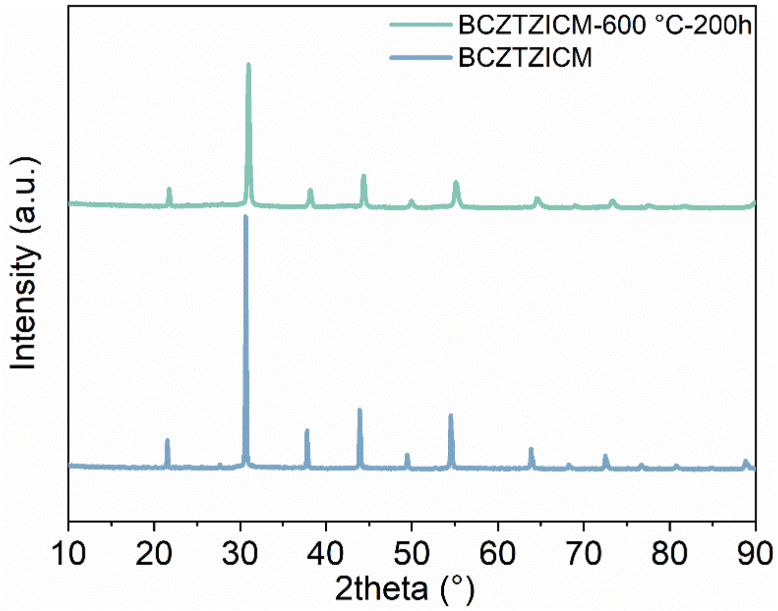

Supplementary Fig. 20.

XRD patterns of the BCZTZICM material after calcination at 600 °C for 200 h in air.

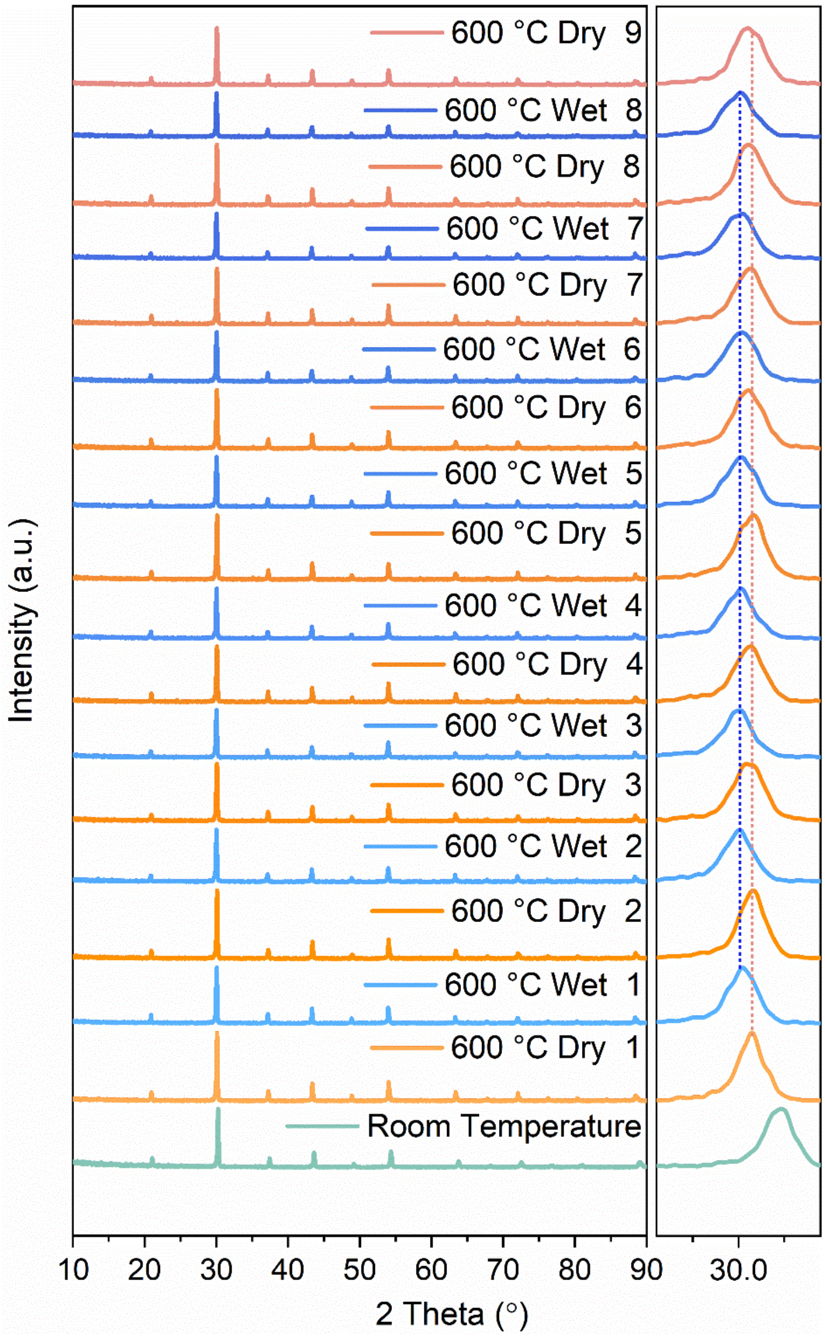

Supplementary Fig. 21.

High temperature XRD patterns of BCZTZICM at 600 °C during switching between dry and humid air atmospheres, and magnified XRD patterns of the selected 2 theta range of 29.55-30.45°.

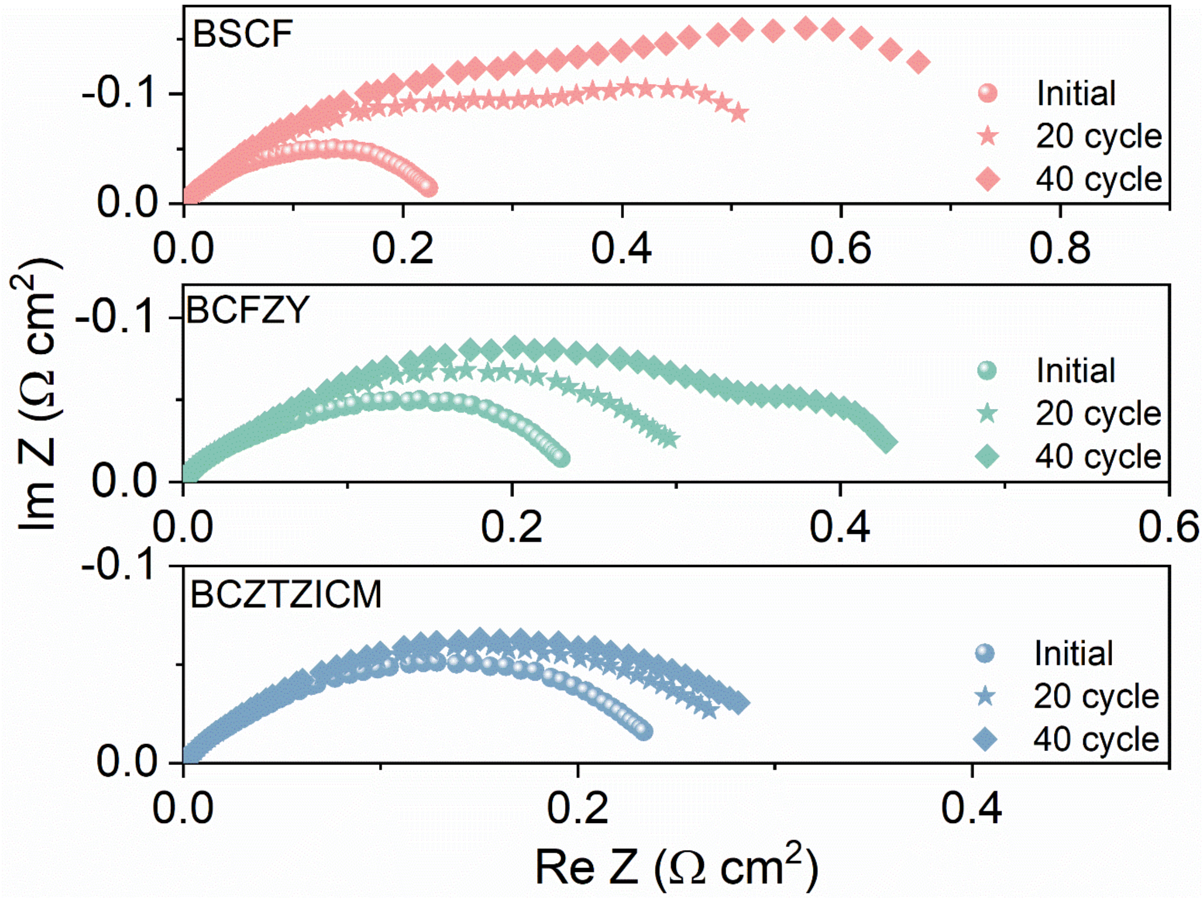

Supplementary Fig. 22.

Polarization resistance of BSCF, BCFZY, and BCZTZICM electrodes initially, after 20 and 40 thermal cycles, respectively.

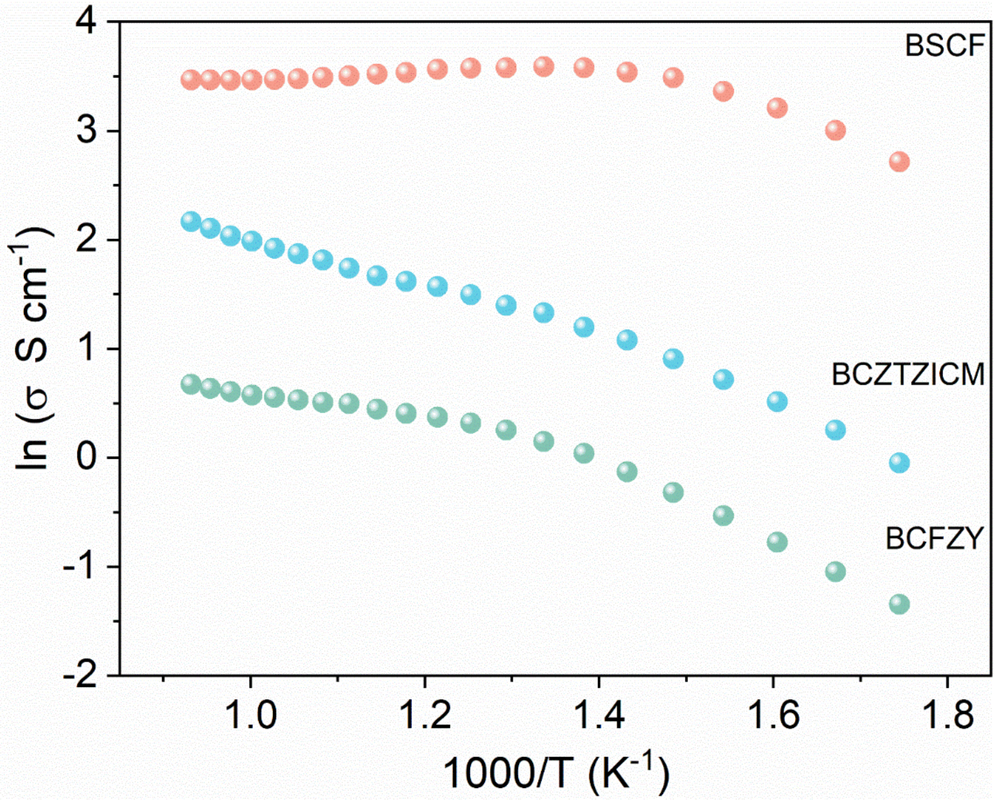

Supplementary Fig. 23.

Electronic conductivities of the BCZTZICM, BSCF, and BCFZY samples at 300-800 °C in air, as measured by the 4-probe DC conductivity technique.

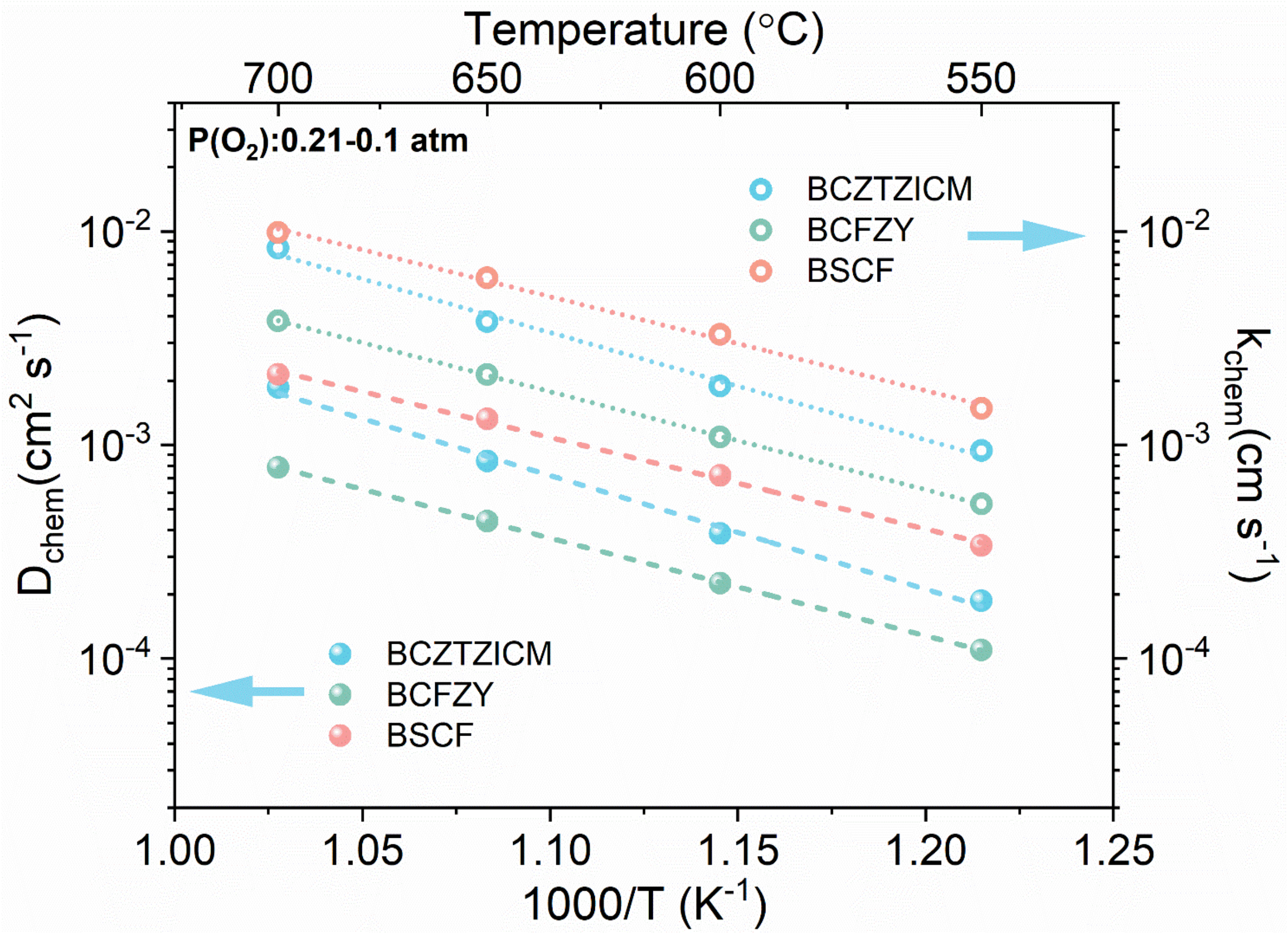

Supplementary Fig. 24.

The fitted values of D_{chem} and k_{chem} of BCZTZICM, BSCF, and BCFZY samples at temperature range of 550-700 °C from electronic conductivity relaxation curves.

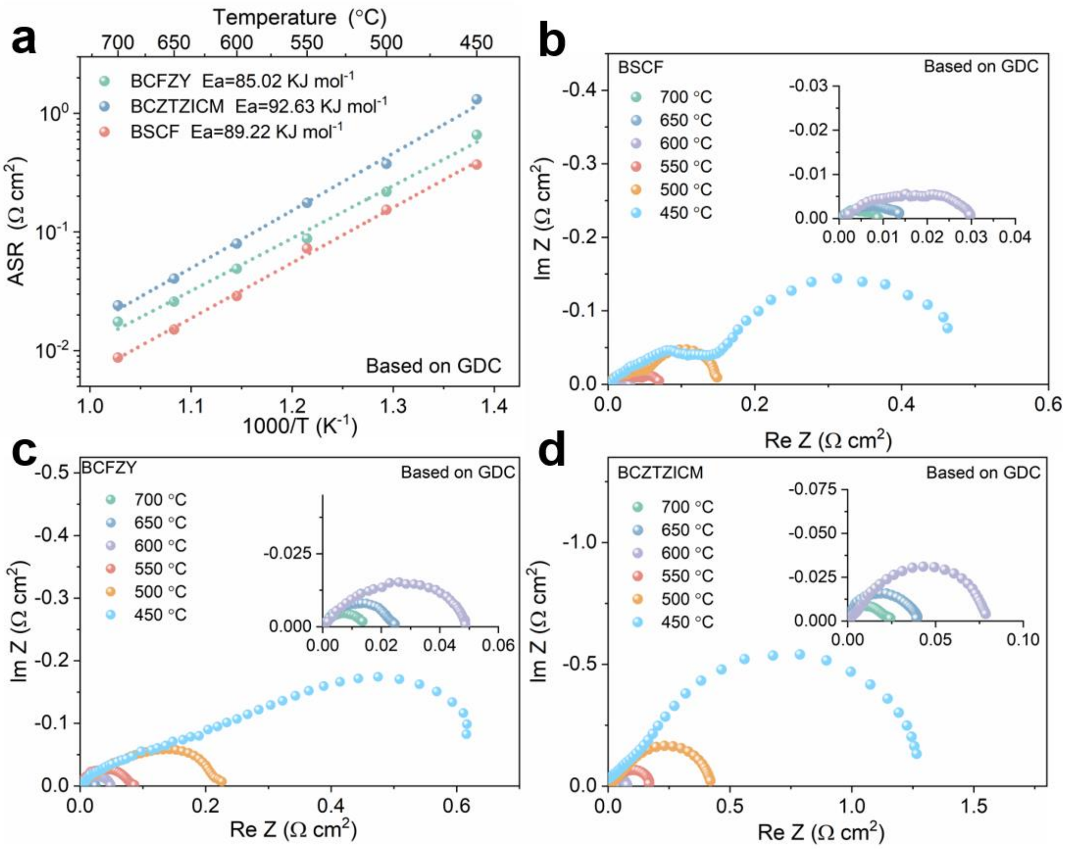

Supplementary Fig. 25.

(a) Arrhenius plots of different electrodes in dry air based on GDC symmetric cells. EIS curves of GDC based-supported symmetrical cells with (b) BSCF, (c) BCFZY and (d) BCZTZICM measured at 500, 550, 600, 650, and 700 °C.

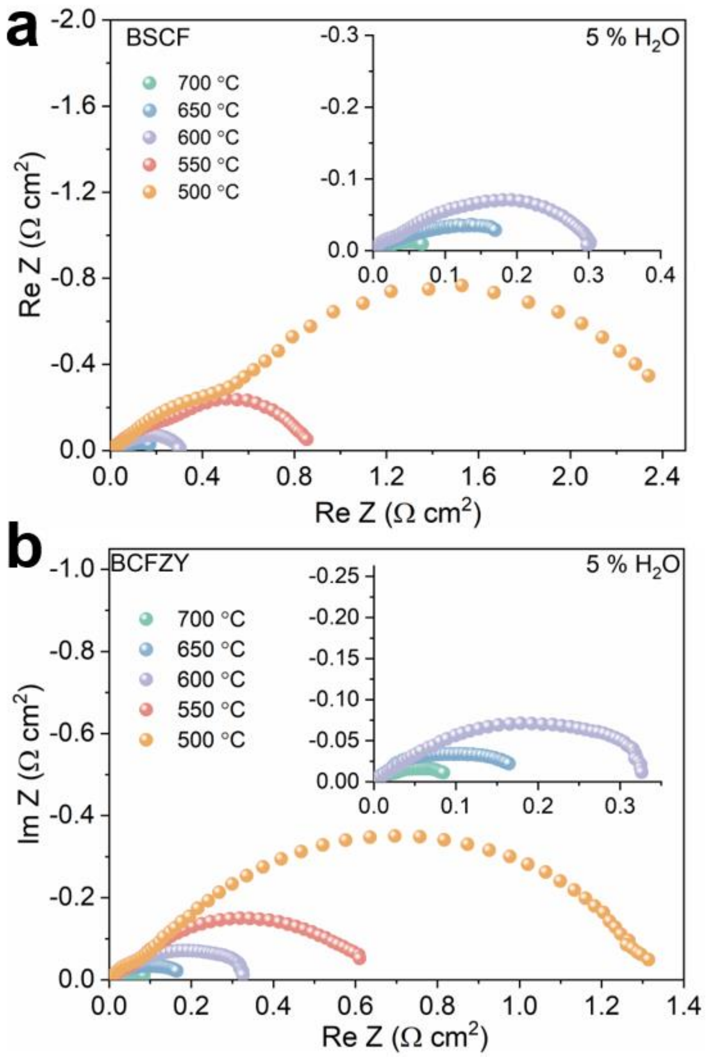

Supplementary Fig. 26.

EIS curves of BZCYYb based-supported symmetrical cell with (a) BSCF, and (b) BCFZY measured at 500, 550, 600, 650, and 700 °C at 5% H_2O atmosphere.

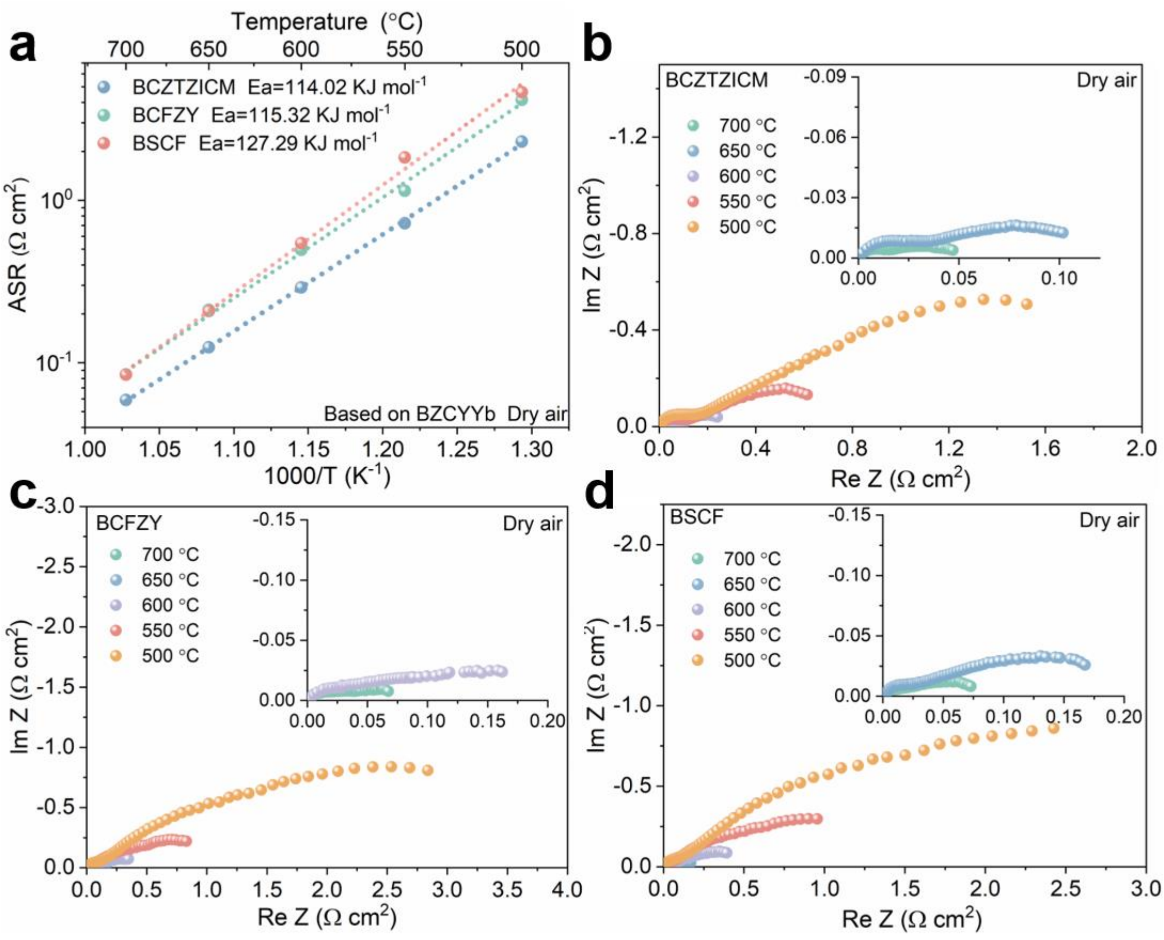

Supplementary Fig. 27.

(a) Arrhenius plots of different electrodes in dry air based on BZCYYb symmetric cells. The EIS plot of the BZCYYb symmetric cell of the (b) BCZTZICM, (c) BCFZY, and (d) BSCF at temperatures between 500 and 700 °C in a dry atmosphere.

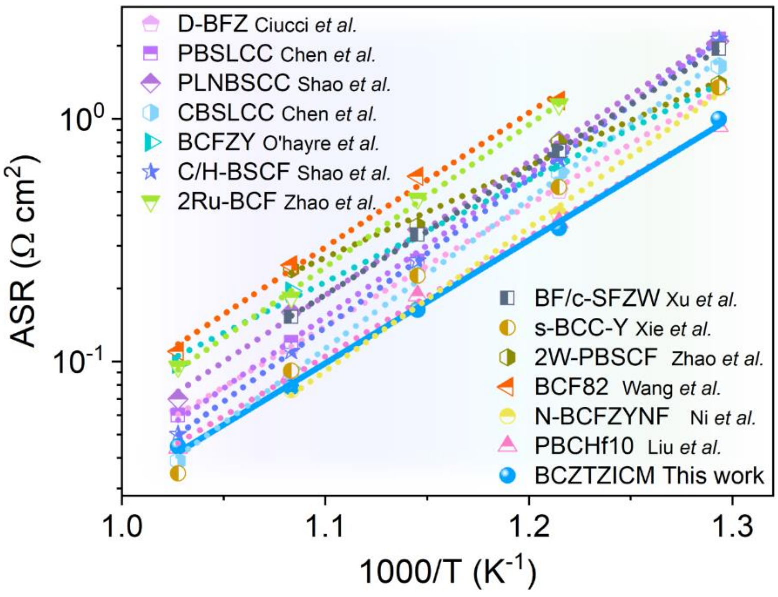

Supplementary Fig. 28.

Arrhenius plots of the ASRs of the D-BFZ, PBSLCC, PLNBSCC, CBSLCC, BCFZY, C/H-BSCF, 2Ru-BCF, BF/C-SFZW, s-BCC-Y, 2W-PBSCF, BCF82, N-BCFZYNF, PBCHf10 and BCZTZICM electrodes[1, 2, 3, 4, 5, 6, 7, 8, 9, 10, 11, 12, 13].

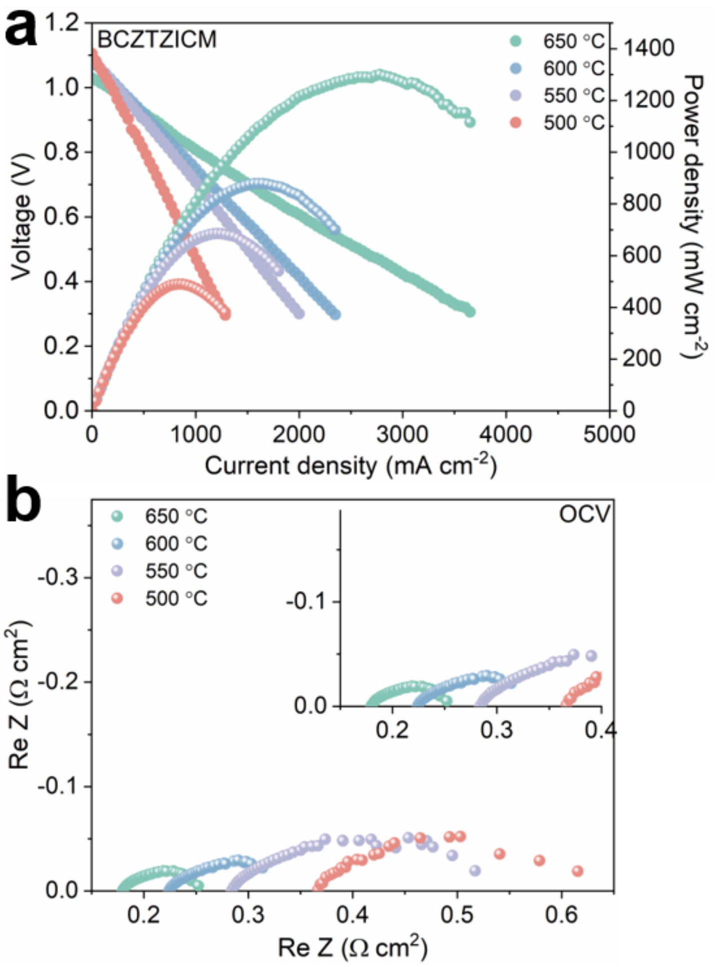

Supplementary Fig. 29.

(a) *I-V-P* curve and corresponding (b) impedance in fuel cell mode at open circuit configured as a Ni-BZCYYb|BZCYYb|BCZTZICM.

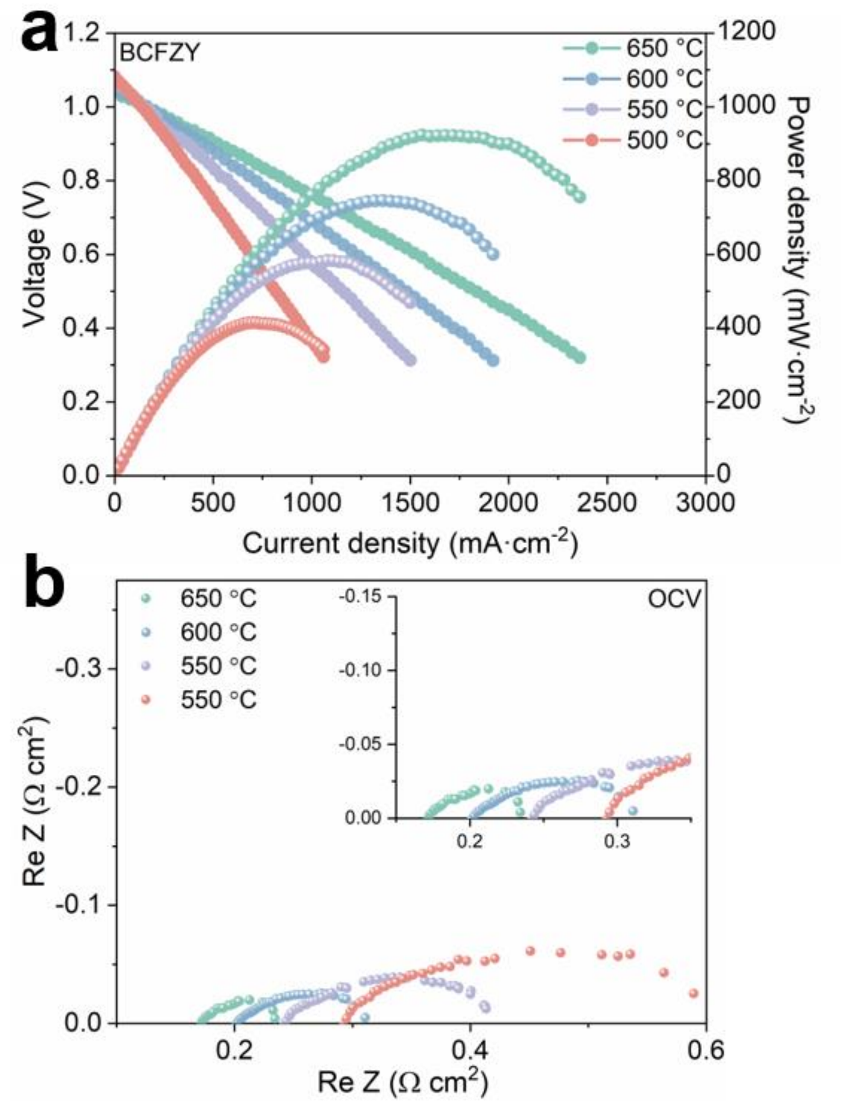

Supplementary Fig. 30.

(a) *I-V-P* curve and corresponding (b) impedance in fuel cell mode at open circuit configured as a Ni-BZCYYb|BZCYYb|BCFZY.

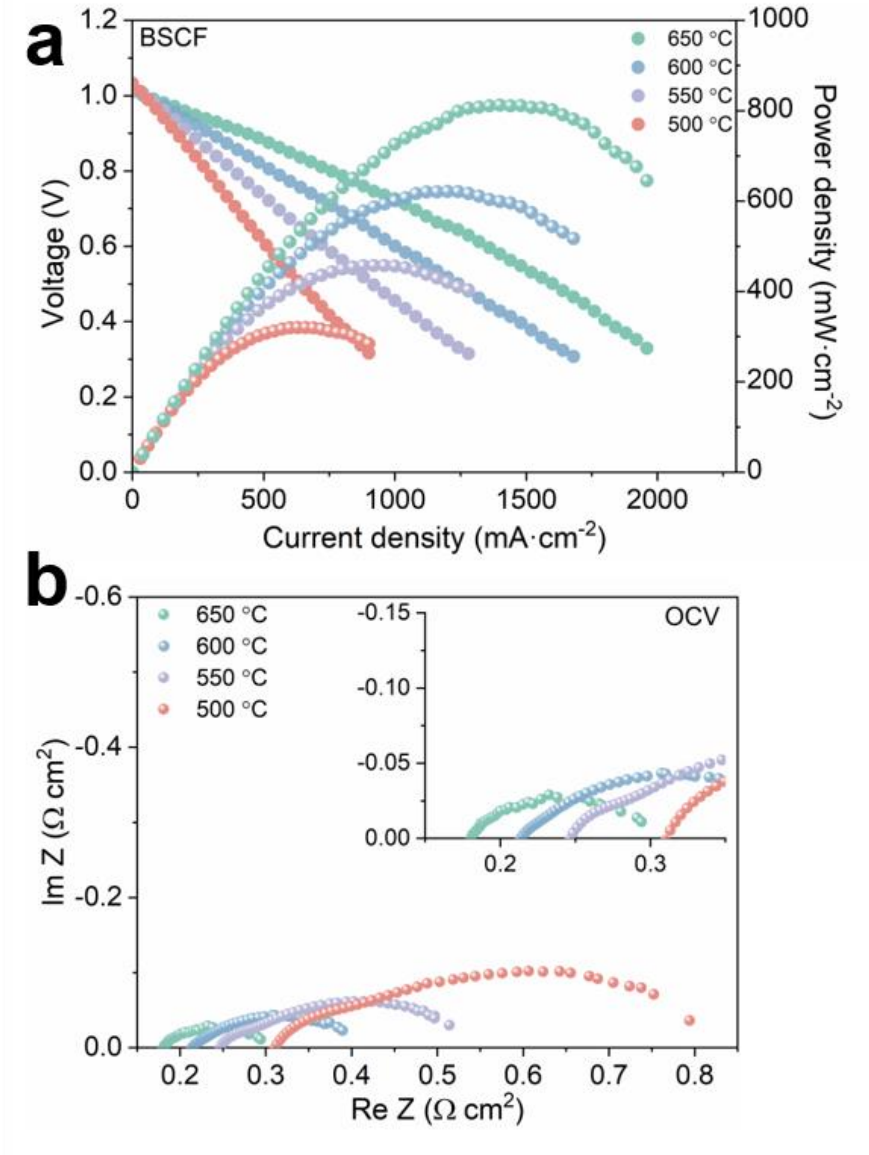

Supplementary Fig. 31.

(a) *I-V-P* curve and corresponding (b) impedance in fuel cell mode at open circuit configured as a Ni-BZCYYb|BZCYYb|BSCF.

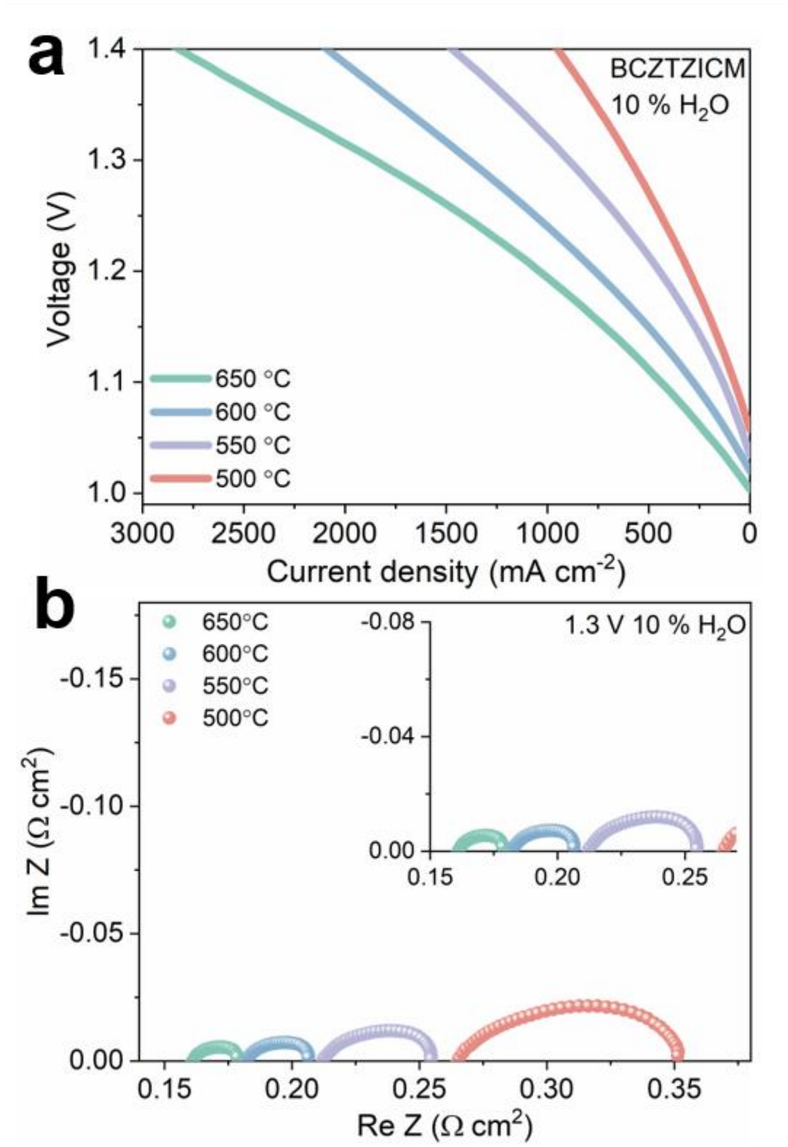

Supplementary Fig. 32.

(a) *I-V* curve and corresponding (b) impedance in electrolysis mode at 1.3V configured as a Ni-BZCYYb|BZCYYb|BCZTZICM.

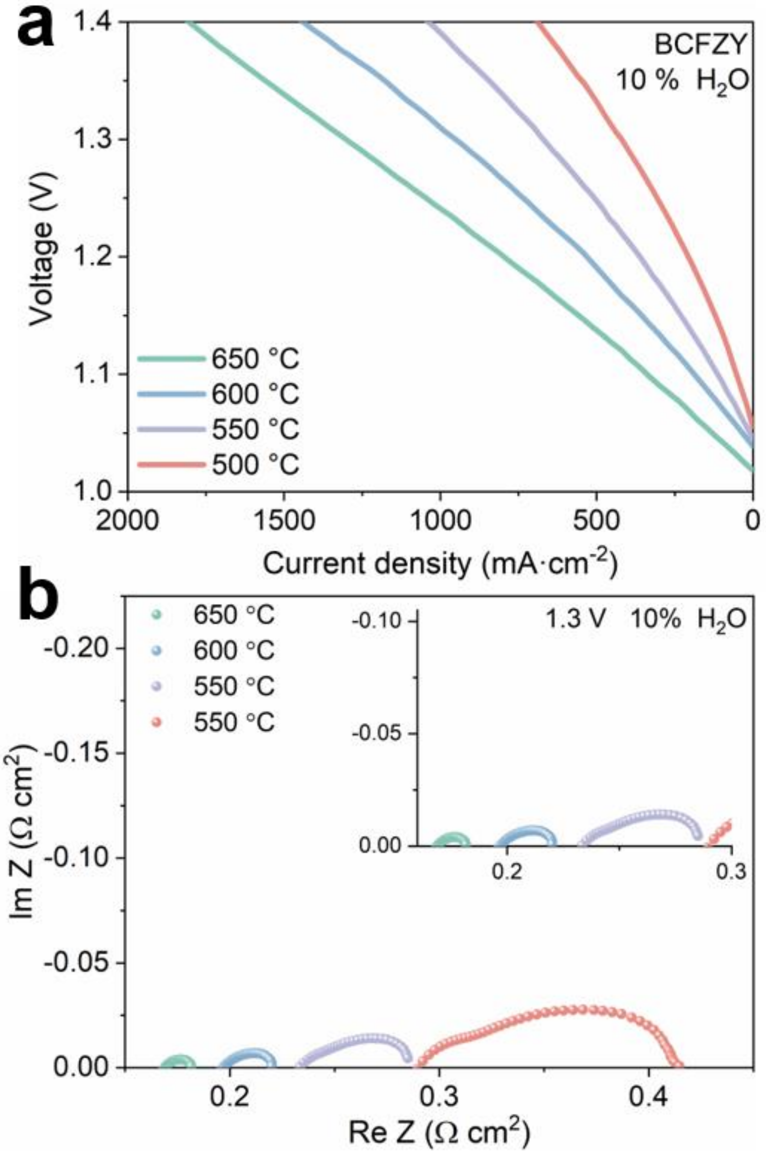

Supplementary Fig. 33.

(a) *I-V* curve and corresponding (b) impedance in electrolysis mode at 1.3V configured as a Ni-BZCYYb|BZCYYb|BCFZY.

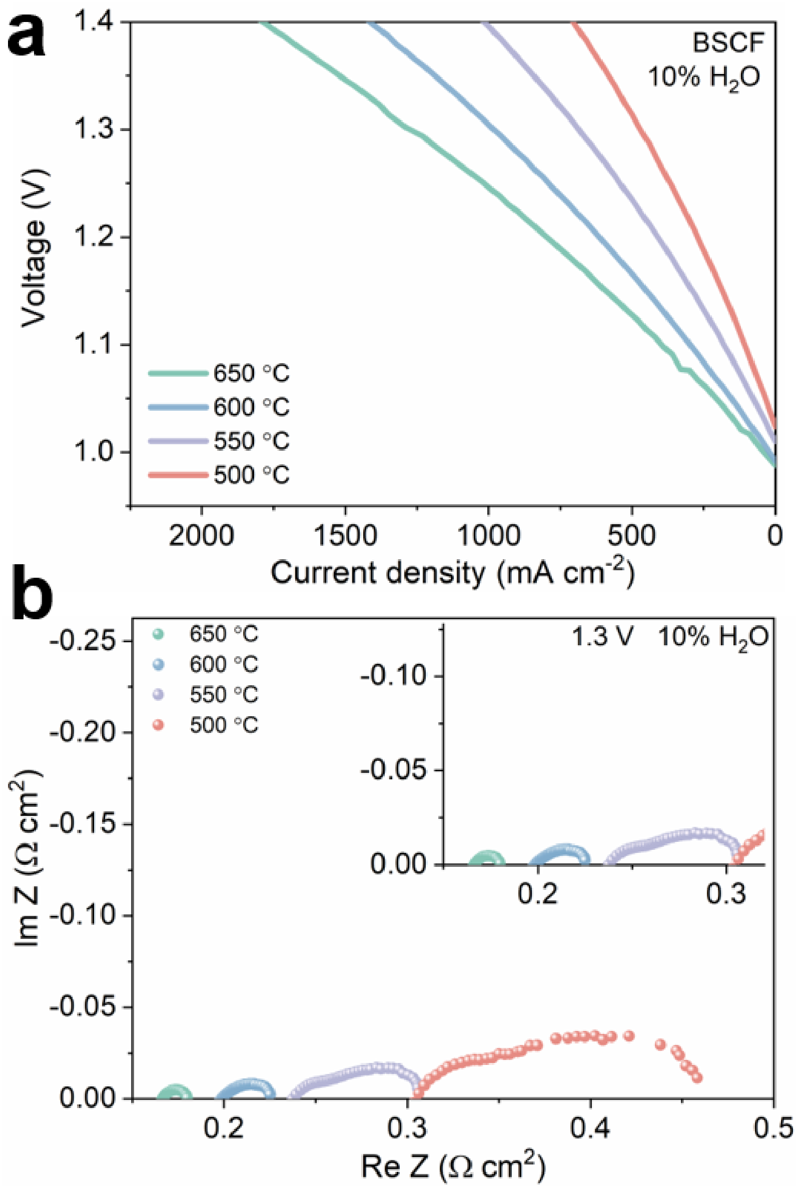

Supplementary Fig. 34.

(a)*I-V* curve and corresponding (b) impedance in electrolysis mode at 1.3V configured as a Ni-BZCYYb|BZCYYb|BSCF.

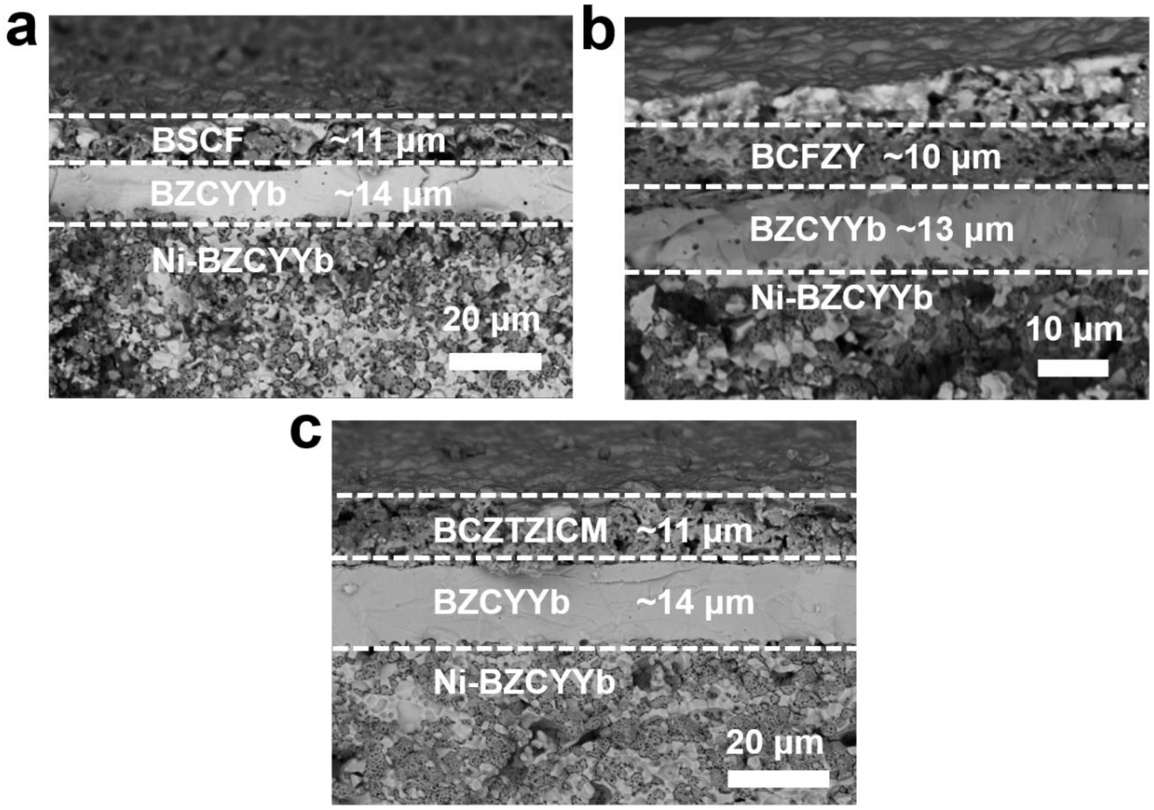

Supplementary Fig. 35.

Cross-sectional morphology of single cells prepared by co-pressing method after testing, with the air electrode configured as (a) BSCF, (b) BCFZY, and (c) BCZTZICM.

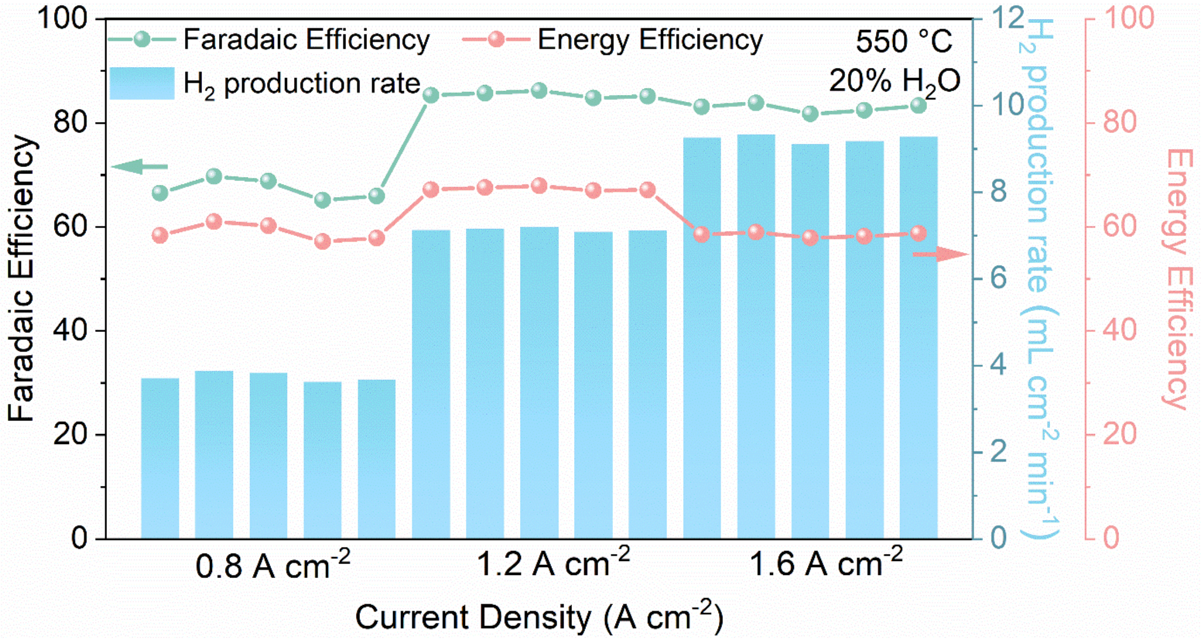

Supplementary Fig. 36.

The faraday efficiency, corresponding H_2 production rate and energy efficiency of tested hydrogen electrode-supported ultrathin BZCYYb electrolyte single cell with BCZTZICM oxygen electrode.

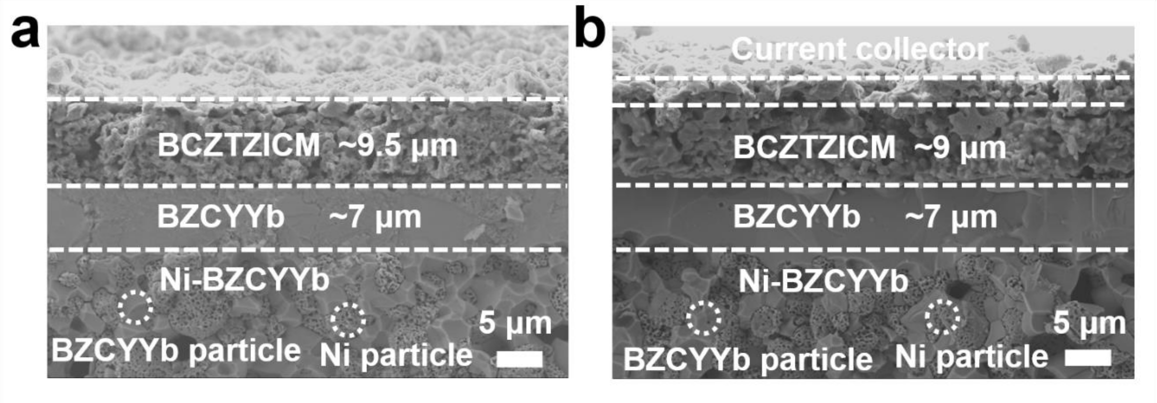

Supplementary Fig. 37.

Tested hydrogen electrode-supported ultrathin electrolyte (~7 µm) BZCYYb single cell with oxygen electrode thickness of 9-10 µm, hydrogen electrode consists of porous Ni metal particles and BZCYYb.

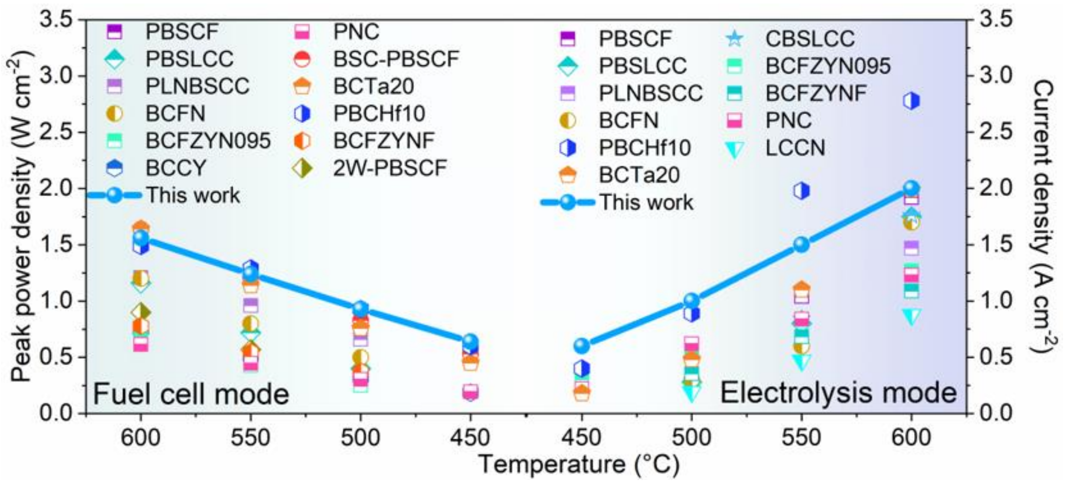

Supplementary Fig. 38.

Performance comparison with leading oxygen electrodes (peak power density in fuel cell mode; current density at 1.3 V in electrolysis mode) [2, 3, 4, 10, 12, 13, 14, 15, 16, 17, 18, 19, 20, 21, 22].

Supplementary Table 1. TEC of oxygen electrodes.

Oxygen electrode	TEC($\times10^{-6}$ K^{-1})	Temperature range (°C)	References
PBSCF [a]	21.3	250-900	[23]
PBSLCC [b]	23.8	300-800	[2]
PBCC [c]	24.5	300-800	[2]
PNC [d]	17.6	100-800	[24]
BCF82 [e]	21.77	25-800	[11]
D-SFN [f]	17.1	100-1000	[25]
BCFZY [g]	22.56	300-800	This work
BSCF [h]	26.08	300-800	This work
BCZTZICM [i]	17.68	300-800	This work
BZCYYb [j]	11.98	300-800	This work
c-SYNC [k]	12.9	100-800	[26]
BSCF-YMO [l]	16.56	25-900	[27]
s-BCC-Y [m]	17.06	300-800	[9]
BSC-PBSCF [n]	18	100-650	[19]
BCCY [o]	15.7	300-800	[17]
C/H-BSCF [p]	19.7	40-1000	[6]
PBCHf10 [q]	24.1	400-800	[12]
SCFN [r]	16.8	200-800	[28]
BB-OPS [s]	20.6	100-700	[29]
BCFZYN-095 [t]	18.7	25-800	[16]
BGCO0.1 [u]	20.46	25-800	[30]
NSTF0.3 [v]	19.9	300-700	[31]
PNCO64 [w]	18.5	25-1000	[32]

[a] $PrBa_{0.5}Sr_{0.5}Co_{1.5}Fe_{0.5}O_{5+\delta}$ (PBSCF). [b] $Pr_{0.2}Ba_{0.2}Sr_{0.2}La_{0.2}Ca_{0.2}CoO_{3-\delta}$ (PBSLCC).[c]

$Pr_{0.8}Ba_{0.8}Ca_{0.4}Co_2O_{5+\delta}$ (PBCC). [d] $PrNi_{0.5}Co_{0.5}O_{3-\delta}$ (PNC). [e] $Ba_{0.8}Ca_{0.2}FeO_{3-\delta}$ (BCF82). [f] $Sr_{2.8}Fe_{1.8}Nb_{0.2}O_{7-\delta}$ (D-SFN). [g] $BaCo_{0.4}Fe_{0.4}Zr_{0.1}Y_{0.1}O_{3-\delta}$ (BCFZY). [h] $Ba_{0.5}Sr_{0.5}Co_{0.8}Fe_{0.2}O_{3-\delta}$ (BSCF). [i] $BaCo_{0.8}(Zr_{1/6}Ti_{1/6}Zn_{1/6}In_{1/6}Cu_{1/6}Mo_{1/6})_{0.2}O_{3-\delta}$ (BCZTZICM). [j] $BaZr_{0.1}Ce_{0.7}Y_{0.1}Yb_{0.1}O_{3-\delta}$ (BZCYYb). [k] $SrNb_{0.1}Co_{0.9}O_{3-\delta}$-20 wt.% $Y_2W_3O_{12}$ (*c*-SYNC). [l] $Ba_{0.5}Sr_{0.5}Co_{0.8}Fe_{0.2}O_{3-\delta}$-30 wt.% $Y_2Mo_3O_{12}$ (BSCF-YMO). [m] $BaCo_{0.7}Ce_{0.15}Y_{0.15}O_{3-\delta}$composites (s-BCC-Y). [n] $Ba_{0.62}Sr_{0.38}CoO_{3-\delta}$-$Pr_{1.44}Ba_{0.11}Sr_{0.45}Co_{1.32}Fe_{0.68}O_{6-\delta}$ (BSC-PBSCF). [o] $BaCo_{0.7}(Ce_{0.8}Y_{0.2})_{0.3}O_{3-\delta}$ composites (BCCY). [p] $Ba_{1.5}Sr_{1.5}Co_{1.6}Fe_{0.4}O_{7-\delta}$ composites (C/H-BSCF). [q] $Pr_0BaCo_{1.9}Hf_{0.1}O_{5+\delta}$ composites (PBCHf10). [r] $Sr_{0.9}Ce_{0.1}Fe_{0.8}Ni_{0.2}O_{3-\delta}$ composites (SCFN). [s] $Ba_{0.5}Sr_{0.5}Co_{0.8}Fe_{0.2}O_{3-\delta}$- $BaZr_{0.1}Ce_{0.7}Y_{0.1}Yb_{0.1}O_{3-\delta}$ in molar ratio of 7:3 via sol-gel (BB-OPS). [t] $Ba_{0.95}(Co_{0.4}Fe_{0.4}Zr_{0.1}Y_{0.1})_{0.95}Ni_{0.05}O_{3-\delta}$ composites (BCFZYN-095). [u] $Ba_{1.1}Gd_{0.9}Co_2O_{6-\delta}$ composites (BGCO0.1). [v] $Na_{0.3}Sr_{0.7}Ti_{0.1}Fe_{0.9}O_{3-\delta}$ composites (NSTF0.3). [w] $Pr_4Ni_{1.8}Co_{1.2}O_{10-\delta}$ composites (PNCO64).

Supplementary Table 2. TEC of oxygen electrodes and crack width of electrode after thermal cycling.

Oxygen electrode materials	TEC ($\times 10^{-6}$ K^{-1})	Thermal cycle conditions			Crack width (μm)	References
		Heating rate (°C min^{-1})	Cooling rate (°C min^{-1})	Cycle number		
SNC (based on SDC symmetric cell)	20.5	30	7.5	40	~1/0.6	[26]
BSCF (based on SDC symmetric cell)	22.4	30	6	40	~0.9	[33]
BCC (based on BZCYYb symmetric cell)	25.89	10	6.7	35	~0.78/0.6	[9]
BCFZY (based on BZCYYb symmetric cell)	22.56	20	10	40	1.38/1.54	This work
BSCF (based on BZCYYb symmetric cell)	26.08	20	10	40	1.89	This work
SF (based on BZCYYb symmetric cell)	30	30	10	35	~0.82/0.75	[8]
PBCF (based on BZCYYb single cell)	18.5	1.6	1.6	5	~4.2	[34]
		10	5	15		
		20	10	50		

Supplementary Table 3. Performance and impedance comparison of oxygen electrodes in fuel cell and electrolysis modes at 600 °C.

Oxygen electrode	Peak power density (mW cm^{-2})	Polarization resistance at OCV (Ω cm^2)	Current density at 1.3 V (mA cm^{-2})	References
CBSLCC	1660	0.112	1760	[4]
PBSLCC	1160	0.112	1750	[2]
BCZTZICM	1560	0.11	2000	This work
PLNBSCC	1210	0.17	1950	[3]
BCT20 with PLD layer	1640	0.12	1600	[35]
BSC-PBSCF	1640	0.04	/	[19]
PBCHf10	1490	0.07	2780	[12]
BCCY	743	0.113	/	[17]
PNMCFC-PBC	1720	0.08	2170	[36]
N-XFN	790	0.167	1700	[37]
BCFN	1207	0.197	1511	[15]
C/H-BSCF	1670	0.157	1230	[6]
BSCF-RC_x	1100	0.13	1490	[38]
NAUP-PNC	1501	0.07	1316	[39]
PBN-BCZYYb CCS	1160	0.25	1460	[40]

Supplementary Table 4. Performance comparison of oxygen electrodes in fuel cell and electrolysis modes at 450 °C.

Oxygen electrode	Electrolyte (μm)	Peak power density ($mW\ cm^{-2}$)	Current density at 1.3 V ($mA\ cm^{-2}$)	Test condition at electrolysis	References
NCCO [a]	BZCYYb [b] (8)	180	560@1.4 V	20%H_2O-80%Air	[41]
PNC [c]	BZCYYb4411 [d] (15)	195	220	10%H_2O-Air<br>Dry H_2	[18]
BCZTZICM [e]	BZCYYb (7)	648	600	10%H_2O-Air<br>Dry H_2	This work
D-SFN [f]	BZCYYb (23)	165	170	Humidified air	[25]
3D-PNC73 [g]	BZCYYb (16)	651	640	30%H_2O-O_2<br>Dry H_2	[42]
BCT20 with PLD layer [h]	BZCYYb (10)	450	200	3%H_2O-Air<br>3%H_2O-H_2	[20]
BSC-PBSCF [i]	BZCYYb4411 (3)	530	640	40%H_2O - 60%N_2	[19]
PBCHf10 [j]	BSCYb172 [k] (7)	600	400	30%H_2O-70%Air<br>3%H_2O-97%H_2	[12]
BCFZYN [l]	BZCYYb (19)	252	300	5%H_2O-Air<br>Dry H_2	[16]
KBCFZY [m]	BZCYYb (7)	210	1100@1.4 V	20%H_2O-80%Air	[43]
BCCY [n]	BZCYYb (16.1)	178	/	/	[17]
PNMCFC-PBC [o]	BZCYYb (16.1)	640	320	3%H_2O-Air	[36]
GCCCO-BCZYYb [p]	BZCYYb (~15)	280	/	/	[44]
PBSCF With PLD layer [q]	BZCYYb (15)	370	/	/	[35]

[a] $Na_{0.15}Ca_{2.85}Co_4O_{9-\delta}$ (NCCO). [b] $BaZr_{0.1}Ce_{0.7}Y_{0.1}Yb_{0.1}O_{3-\delta}$ (BZCYYb). [c] $PrNi_{0.5}Co_{0.5}O_{3-\delta}$ (PNC). [d] $BaZr_{0.4}Ce_{0.4}Y_{0.1}Yb_{0.1}O_{3-\delta}$ (BZCYYb4411). [e] $BaCo_{0.8}(Zr_{1/6}Ti_{1/6}Zn_{1/6}In_{1/6}Cu_{1/6}Mo_{1/6})_{0.2}O_{3-\delta}$

(BCZTZICM). [f] $Sr_{2.8}Fe_{1.8}Nb_{0.2}O_{7-\delta}$ (D-SFN). [g] three-dimensional $PrNi_{0.7}Co_{0.3}O_{3-\delta}$ mesh (3D-PNC73). [h] $BaCo_{0.8}Ta_{0.2}O_{3-\delta}$ (BCT20 with PLD layer). [i] $Ba_{0.62}Sr_{0.38}CoO_{3-\delta}$-$Pr_{1.44}Ba_{0.11}Sr_{0.45}Co_{1.32}Fe_{0.68}O_{6-\delta}$ composites (BSC-PBSCF). [j] $PrBaCo_{1.9}Hf_{0.1}O_{5+\delta}$ composites (PBCHf10). [k] $BaSn_{0.1}Ce_{0.7}Yb_{0.2}O_{3-\delta}$ (BSCYb172). [l] $Ba_{0.95}(Co_{0.4}Fe_{0.4}Zr_{0.1}Y_{0.1})_{0.95}Ni_{0.05}O_{3-\delta}$ composites (BCFZYN). [m] $K_{0.05}Ba_{0.95}Co_{0.4}Fe_{0.4}Zr_{0.18}Y_{0.02}O_{3-\delta}$ (KBCFZY). [n] $BaCo_{0.7}(Ce_{0.8}Y_{0.2})_{0.3}O_{3-\delta}$ composites (BCCY). [o] $PrNi_{0.2}Mn_{0.2}Co_{0.2}Fe_{0.2}Cu_{0.2}O_{3-\delta}$ coating $PrBaCo_2O_{5+\delta}$ (PNMCFC-PBC). [p] $Gd_{0.3}Ca_{2.7}Co_{3.82}Cu_{0.18}O_{9-\delta}$-$BaZr_{0.1}Ce_{0.7}Y_{0.1}Yb_{0.1}O_{3-\delta}$ composites (GCCCO-BZCYYb). [q] $PrBa_{0.5}Sr_{0.5}Co_{1.5}Fe_{0.5}O_{5+\delta}$ with PBSCF PLD film (PBSCF With PLD layer).

Supplementary Table 5. Stability and degradation rate comparison of oxygen electrodes in fuel cell and electrolysis modes.

Oxygen electrode	Electrolyte	Degradation rate	Current density ($mA\ cm^{-2}$)	Time (h)	Test condition	Ref
BCFZY	BZCYYb	< 30 mV per 1000 h	-1385	600	20% H_2O-Air	45
			-1385	1200	10% H_2O-Air	
BSC-PBSCF	BZCYYb4411	< 0.07 mV h^{-1}	300	100	H_2-Air	19
		< 0.15 mV h^{-1}	-600	260	/	
PNC55	BZCYYb	1.98%	1.4 V	200	30%H_2O-O_2	42
BCZTZICM	BZCYYb	19.3 μV h^{-1}	300/500	780	80 mL 10% H_2O-Air 60 mL Dry H_2	This work
		16.9μV h^{-1}	-300/-500		80 mL Dry Air 60 mL Dry H_2	
PrO_x-PNC	BZCYYb4411	0.077 mA cm^{-2} h^{-1} (0.35%)	0.75 V	80	20 sccm H_2 40 sccm O_2	46
		0.134 mA cm^{-2} h^{-1} (0.57%)	1.25 V	80	Humidified O_2	
NAUP-PNC	BZCYYb4411	1.03 %	1.3 V	105	20%H_2O-O_2	39
		1.24 %	1.4 V			
PBNO-BCZYYb CCS	BZCYYb	slight activation	400	1018	10% H_2O-Air Dry H_2	40
		9 μV h^{-1}		759	20% H_2O-Air Dry H_2	
		1 μV h^{-1}	-1500	5013	40% H_2O-Air Dry H_2	
PBNO-BCZYYb4411 CCS		3 μV h^{-1}	-1000	2616		
PBNO-BCY CCS		10 μV h^{-1}		1041		

Supplementary Note 1

For $A_nB_mO_\delta$-type oxides, we calculated the change in lattice oxygen content by testing the change in its ion valence and normalizing it using the following equation.

$$n(O) = \frac{\Delta A * n + \Delta B * m}{2}$$

Where n(O) represents the change in lattice oxygen content, ΔA and ΔB are the changes in the valence state of A and B ions, respectively.

Supplementary Note 2

The proton uptake has the beneficial effect of enhancing the absorption of protons by the electrode, thereby strengthening the proton transport capacity. In general, proton defects can be generated by hydration reactions with oxygen vacancies, as illustrated by the following formula:

$$H_2O + O_O^{\times} + V_O^{\cdot\cdot} = 2OH_O^{\cdot}$$

Where $O_O^{\times}$ represents lattice oxygen, $V_O^{\cdot\cdot}$ is oxygen vacancies, and $OH_O^{\cdot}$ represents proton defects. Beyond classical hydration pathways, PCEC oxygen electrodes can generate protonic defects through transition metal oxide mediated routes[47, 48].

$$Mn(\mathrm{III})_{Mn}^{x} + O_O^{\cdot} + H_2O + V_O^{\cdot\cdot} = Mn(\mathrm{IV})_{Mn}^{\cdot} + 2OH_O^{\cdot}$$

Where $h^{\cdot}$ represents electronic holes, $Mn(\mathrm{III})_{Mn}^{x}$ is Mn^{3+} ion. The proposed mechanisms, extendable to Fe/Co/Ni systems based on their redox activity[1, 31]. This mechanism is derived from lowering of antibonding O *2p* states that hybridize with transition metal *3d* orbitals, coupled with the influence of incorporated protonic defects on the local coordination environment of transition metal ions. It also has been experimentally validated in relevant perovskite electrodes. It should be noted that this oxidative proton uptake mechanism is not universally observed across all perovskite compositions, depending on the degree of metal-oxygen covalency, the position of the Fermi level relative to the oxygen *2p* band, and the local coordination flexibility around B-site cations[49].

References

1. Wang, Z. *et al.* Rational design of perovskite ferrites as high-performance proton-conducting fuel cell cathodes. *Nat. Catal.* **5**, 777-787 (2022).

2. He, F. *et al.* An efficient high-entropy perovskite-type air electrode for reversible oxygen reduction and water splitting in protonic ceramic cells. *Adv. Mater.* **35**, 202209469 (2023).

3. Liu, Z. *et al.* High-entropy perovskite oxide: a new opportunity for developing highly active and durable air electrode for reversible protonic ceramic electrochemical cells. *Nano Micro Lett.* **14**, 217 (2022).

4. He, F. *et al.* Phase segregation of a composite air electrode unlocks the high performance of reversible protonic ceramic electrochemical cells. *Energy Environ. Sci.* **17**, 3898-3907 (2024).

5. Duan, C. *et al.* Readily processed protonic ceramic fuel cells with high performance at low temperatures. *Science* **349**, 1321-1326 (2015).

6. Liu, Z. *et al.* Synergistic dual-phase air electrode enables high and durable performance of reversible proton ceramic electrochemical cells. *Nat. Commun.* **15**, 472 (2024).

7. Zhao, S. *et al.* Single-atom enables reverse hydrogen spillover for high-performance protonic ceramic fuel cells. *Adv. Mater.* **37**, 2501387 (2025).

8. Shi, K. *et al.* A Core-shell perovskite composite air electrode with thermal-expansion offset and mechanical support functions for highly durable reversible protonic ceramic cells. *Adv. Mater.* **37**, 2419224 (2025).

9. Yang, H. *et al.* Hydration-induced stiffness enabling robust thermal cycling of high temperature fuel cells cathode. *Nat. Commun.* **16**, 3154 (2025).

10. Zhao, S. *et al.* Reverse Atom capture on perovskite surface enabling robust and efficient cathode for protonic ceramic fuel cells. *Adv. Mater.* **36**, 2405052 (2024).

11. Zhang, G. *et al.* A promising strontium and cobalt-free $Ba_{1-x}Ca_xFeO_{3-\delta}$ air electrode for reversible protonic ceramic cells. *Appl. Catal. B-Environ. Energy* **355**, 124176 (2024).

12. Hu, X. *et al.* Data-driven discovery of electrode materials for protonic ceramic cells. *Energy Environ. Sci.* **17**, 9335-9345 (2024).

13. Chen, X. *et al.* Synergistic bulk and surface engineering for expeditious and durable reversible protonic ceramic electrochemical cells air electrode. *Adv. Mater.* **36**, 2403998 (2024).

14. Choi, S. *et al.* Protonic ceramic electrochemical cells for hydrogen production and electricity generation: exceptional reversibility, stability, and demonstrated faradaic efficiency. *Energy Environ. Sci.* **12**, 206-215 (2019).

15. Pei, K. *et al.* Surface restructuring of a perovskite-type air electrode for reversible protonic ceramic electrochemical cells. *Nat. Commun.* **13**, 2207 (2022).

16. Liang, M. *et al.* High-temperature water oxidation activity of a perovskite-based nanocomposite towards application as air electrode in reversible protonic ceramic cells. *Appl. Catal. B* **331**, 122682 (2023).

17. Song, Y. *et al.* Self-assembled triple-conducting nanocomposite as a superior protonic ceramic fuel cell cathode. *Joule* **3**, 2842-2853 (2019).

18. Ding, H. *et al.* Self-sustainable protonic ceramic electrochemical cells using a triple conducting electrode for hydrogen and power production. *Nat. Commun.* **11**, 1907 (2020).

19. Liu, F. *et al.* Lowering the operating temperature of protonic ceramic electrochemical cells to <450 °C. *Nat. Energy* **8**, 1145-1157 (2023).

20. Kim, J.H. *et al.* An universal oxygen electrode for reversible solid oxide electrochemical cells at reduced temperatures. *Energy Environ. Sci.* **16**, 3803-3814 (2023).

21. Wang, N. *et al.* Machine-learning-accelerated development of efficient mixed protonic-electronic conducting oxides as the air electrodes for protonic ceramic cells. *Adv. Mater.* **34**, 2203446 (2022).

22. Liang, M. *et al.* A new durable surface nanoparticles-modified perovskite cathode for protonic ceramic fuel cells from selective cation exsolution under oxidizing atmosphere. *Adv. Mater.* **34**, e2106379 (2022).

23. Jiang, L. *et al.* Thermal and electrochemical properties of $PrBa_{0.5}Sr_{0.5}Co_{2-x}Fe_xO_{5+\delta}$ (x=0.5, 1.0, 1.5) cathode materials for solid-oxide fuel cells. *J. Power Sources* **232**, 279-285 (2013).

24. Sozal, M.S.I. *et al.* Electrical, thermal, and H_2O and CO_2 poisoning behaviors of $PrNi_{0.5}Co_{0.5}O_{3-\delta}$ electrode for intermediate temperature protonic ceramic electrochemical cells. *Int. J. Hydrogen Energy* **47**, 21817-21827 (2022).

25. Yu, N. *et al.* Rational design of ruddlesden-popper perovskite ferrites as air electrode for highly active and durable reversible protonic ceramic cells. *Nano Micro Lett.* **16**, 177 (2024).

26. Zhang, Y. *et al.* Thermal-expansion offset for high-performance fuel cell cathodes. *Nature* **591**, 246-251 (2021).

27. Lin, P. *et al.* $Y_2Mo_3O_{12}$-$Ba_{0.5}Sr_{0.5}Co_{0.8}Fe_{0.2}O_{3-\delta}$ cathode catalyst for proton-conducting solid oxide fuel cells. *J. Power Sources* **551**, 232073 (2022).

28. Song, Y. *et al.* A cobalt-free multi-phase nanocomposite as near-ideal cathode of intermediate-temperature solid oxide fuel cells developed by smart self-assembly. *Adv. Mater.* **32**, 1906979 (2020).

29. Liu, Z. *et al.* One-pot derived thermodynamically quasi-stable triple conducting nanocomposite as robust bifunctional air electrode for reversible protonic ceramic cells. *Appl. Catal. B* **319**, 121929 (2022).

30. Li, X. *et al.* Spontaneous growth of perovskite-derived oxide over double perovskite surface for enhancing cathodic performance in protonic ceramic fuel cells. *Adv. Energy Mater.* **14**, 2400319 (2024).

31. Zhou, C. *et al.* New strategy for boosting cathodic performance of protonic ceramic fuel cells through incorporating a superior hydronation second phase. *Energy Environ. Mater.* **7**, 12660 (2023).

32. Xia, J. *et al.* Regulation of Ni/Co ratio and gas transport in high-order ruddlesden-popper perovskite air electrodes for protonic ceramic electrochemical cells. *Adv. Funct. Mater.* **34**, 202403493 (2024).

33. Zhang, Y. *et al.* Interfacial oxide wedging for mechanical-robust electrode in high-temperature ceramic cells. *Nat. Commun.* **16**, 4146 (2025).

34. Gao, Y. *et al.* Self-assembled composite cathodes with TEC gradient for proton-conducting solid oxide fuel cells. *Adv. Funct. Mater.* **35**, 202416625 (2024).

35. Choi, S. *et al.* Exceptional power density and stability at intermediate temperatures in protonic ceramic fuel cells. *Nat. Energy* **3**, 202-210 (2018).

36. He, F. *et al.* Conformal high-entropy oxide coatings enable fast and durable surface oxygen reactions. *Joule* **9**, 101957 (2025).

37. Hu, A. *et al.* High-entropy driven self-assembled dual-phase composite air electrodes with enhanced performance and stability for reversible protonic ceramic cells. *Adv. Energy Mater.* **15**, 2405466 (2025).

38. Liu, Z. *et al.* Strategic atomic trapping at heterointerfaces for protonic ceramic cells. *Nat. Commun.* **16**, 10405 (2025).

39. Zheng, S. *et al.* Enhancing surface activity and durability in triple conducting electrode for protonic ceramic electrochemical cells. *Nat. Commun.* **16**, 4146 (2025).

40. Tian, H. *et al.* Conformally coated scaffold design using water-tolerant $Pr_{1.8}Ba_{0.2}NiO_{4.1}$ for protonic ceramic electrochemical cells with 5,000-h electrolysis stability. *Nat. Energy*, 890-903 (2025).

41. Park, K. *et al.* Understanding the highly electrocatalytic active mixed triple conducting $Na_xCa_{3-x}Co_4O_{9-\delta}$ oxygen electrode materials. *Adv. Energy Mater.* **13**, 2202999 (2022).

42. Bian, W. *et al.* Revitalizing interface in protonic ceramic cells by acid etch. *Nature* **604**, 479-485 (2022).

43. Park, K. *et al.* Water-mediated exsolution of nanoparticles in alkali metal-doped perovskite structured triple-conducting oxygen electrocatalysts for reversible cells. *Energy Environ. Sci.* **17**, 1175-1188 (2024).

44. Saqib, M. *et al.* Transition from perovskite to misfit-layered structure materials: a highly oxygen deficient and stable oxygen electrode catalyst. *Energy Environ. Sci.* **14**, 2472-2484 (2021).

45. Duan, C. *et al.* Highly efficient reversible protonic ceramic electrochemical cells for power generation and fuel production. *Nat. Energy* **4**, 230-240 (2019).

46. Zheng, S. *et al.* Hybridizing electrode interface structures in protonic ceramic cells for durable, reversible hydrogen and power generation. *Adv. Mater.* **37**, 2503649 (2025).

47. Wang, N. *et al.* Incorporation of bulk proton carriers in cubic perovskite manganite driven by interplays of oxygen and manganese redox. *Chem. Mater.* **31**, 8383-8393 (2019).

48. Liu, Z. *et al.* Advanced electrode materials for efficient hydrogen production in protonic ceramic electrolysis cells. *Adv. Mater.* **37**, 250369 (2025).

49. Wang, N. *et al.* $La_{0.8}Sr_{0.2}Co_{1-x}Ni_xO_{3-\delta}$ as the efficient triple conductor air electrode for protonic ceramic cells. *ACS Appl. Energy Mater.* **4**, 554-563 (2020).
