# Supplementary material for: Highly ionic-dispersed oxygen electrode for reversible proton ceramic electrochemical cells
Source: Nat Commun. 2026 Mar 14;17:3989. doi: 10.1038/s41467-026-70738-z (PMC13136414; doi:10.1038/s41467-026-70738-z)
Supplement: Supplementary file 2 — Description of Additional Supplementary Files [file 41467_2026_70738_MOESM2_ESM.pdf]

Description of Additional Supplementary Files

File Name: Supplementary Data 1

Description: Lattice parameter and atomic coordinate information of the unit cell model used in theoretical calculations for BCZTZICM, BSCF, and BCFZY.
