# Supplementary material for: Highly ionic-dispersed oxygen electrode for reversible proton ceramic electrochemical cells
Source: Nat Commun. 2026 Mar 14;17:3989. doi: 10.1038/s41467-026-70738-z (PMC13136414; doi:10.1038/s41467-026-70738-z)
Supplement: Supplementary file 3 — Supplementary Data 1 [file 41467_2026_70738_MOESM3_ESM.pdf]

Supplementary Data 1

Highly ionic-dispersion oxygen electrode for proton ceramic electrochemical cells

Xiaoyu Wang[1,2], Zhaohui Cai[2], Zeping Chen[1,2], Donliang Liu[1,2], Wanqing Chen[1,2], Jianqiu Zhu[3], Wenhuai Li[1], Xixi Wang[4], Linjuan Zhan[3], Wei Wang[1,2], Chuan Zhou[1,2], Wei Zhou[1,2]*, Zongping Shao[5]**

[1]State Key Laboratory of Materials-Oriented Chemical Engineering, College of Chemical Engineering, Nanjing Tech University, Nanjing 211816, China.

[2]Suzhou Laboratory, Suzhou 215000, China

[3]Key Laboratory of Interfacial Physics and Technology, Shanghai Institute of Applied Physics, Chinese Academy of Sciences, Shanghai 201800, China

[4]School of Environmental Science and Engineering, Nanjing Tech University, Nanjing 211816, China

[5]Curtin Centre for Advanced Energy Materials and Technologies (CAEMT), Western Australian School of Mines (WASM), Curtin University, Perth, Western Australia 6102, Australia

*Corresponding author E-mail: zhouc@szlab.ac.cn (C. Zhou), zhouwei1982@njtech.edu.cn (W. Zhou), Zongping.Shao@curtin.edu.au (Z. Shao).

Atomic coordinates of $BaCo_{0.8}(Zr_{1/6}Ti_{1/6}Zn_{1/6}In_{1/6}Cu_{1/6}Mo_{1/6})_{0.2}O_{3-\delta}$ for optimization calculation.

1.0

11.8719997406	0.0000000000	0.0000000000
0.0000000000	11.8719997406	0.0000000000
0.0000000000	0.0000000000	25.8934001923

Ba	Co	In	Zr	Zn	Ti	Mo	Cu	O
27	21	1	1	1	1	1	1	81

Cartesian

1.967404053	1.962239733	1.010826557
1.963806837	1.990043957	4.899005423
1.892491735	1.984665941	8.991560897
5.939169694	1.989177301	1.063234799
5.946696542	2.020899284	4.913712874
6.075887643	2.073670323	8.920069219
9.921406439	1.977067861	0.997905750
9.898410376	1.970383925	4.908223473
9.844190953	1.958108277	8.963259411
1.991219284	5.910035807	0.951608350
1.943256406	5.925516895	4.875442429
1.858941463	5.941734046	9.021053480
5.963649758	5.914238495	1.039568231
5.970321822	5.891931007	4.870678043
5.994516957	5.899149183	8.885760464
9.844772681	5.924282207	0.963208594
9.885695464	5.968244222	4.887508753
9.884603240	5.969312702	8.838401435
1.998971700	9.934940519	0.987470710
1.951709269	9.879557640	4.847632917
1.971214965	9.918592775	9.319837425
5.960064414	9.901354632	1.048760388
5.972660605	9.901425864	4.853225891
5.920186367	9.890693576	8.963621918
9.827665129	9.905034952	0.988532339
9.869478312	9.854519593	4.845639125
9.861987081	9.895133704	8.896661585
11.861872925	0.016917600	2.855912574
11.821650590	11.723576000	6.738161852
11.470025701	11.827954622	10.948118002
3.942299338	0.032018783	2.868729807
4.191920004	0.451598998	10.887889953
7.919585459	0.022782368	2.906275238
7.938699379	11.843079549	6.851160650
7.849718740	11.720156864	10.844958696
0.051227679	3.958991369	2.835793402

0.006304032	3.894395819	6.690776929
3.906327179	3.963015977	2.796746155
3.954622474	3.973819497	6.909265440
3.781018221	4.042629608	10.807827560
8.104147567	3.914281418	2.879760396
8.003603601	4.359315201	10.892007004
11.836704285	7.928750643	6.860042087
11.414381639	7.781929622	10.883798796
3.953874538	7.902240467	2.740350329
4.063678663	7.729680951	10.942809855
8.008946001	7.953206962	6.865945782
7.801708374	7.985771858	10.818107240
3.926058442	0.036731967	6.922730008
11.772536127	3.923600938	11.239496435
7.969614066	3.972715401	6.848027549
11.851912317	7.909791059	2.738926192
3.917154442	7.861970644	6.911129765
7.896173875	7.902311699	2.853556275
11.862810813	11.864081117	0.961654990
0.001578976	11.865671965	4.827617319
0.042228703	0.008927744	9.193607099
1.944229910	11.860543261	2.855757214
1.758088826	0.007574336	6.995775290
2.650020294	0.107382238	11.365312466
0.012465600	2.004694004	2.853271447
11.838936221	2.085019954	6.956831616
11.728680960	1.728871834	11.282608946
3.962873513	0.011598944	0.997180735
3.925096810	11.841892349	4.757912285
3.966482601	0.029739359	9.093813934
5.930206334	0.055145439	2.898429537
6.067707835	11.860448285	6.889405202
5.876473664	11.839387357	11.028983091
3.889789483	1.999897716	2.896254492
3.884233387	2.143347089	6.983009844
4.171559525	2.292768078	10.920334384
7.912628467	0.057519839	1.014218592
7.949550386	0.029395071	4.841651542
7.909636723	11.809458046	8.927966706
9.932269319	0.021951328	2.873701340
9.974248710	11.821864286	6.894480309
9.698212844	11.628991778	11.030588482
7.949787826	2.037603187	2.890842771
7.998356177	1.857623671	6.942124165

7.892303604	1.500027167	11.139392550
0.045161087	3.959858025	0.927164981
0.011812640	3.971433225	4.796648812
11.764249471	3.937787978	9.349200540
1.993391860	3.963134697	2.877896071
1.799486489	4.031648008	6.983501819
2.018607988	3.967230537	11.180718416
0.036625119	5.932794430	2.837217539
0.013759648	6.079888507	6.953258327
11.762563647	6.073857531	11.288253707
3.921262154	3.941622634	0.933275823
3.869630827	3.994001897	5.068167006
3.900711723	4.050215816	8.866288627
5.690035780	3.943878314	2.901484959
5.826836833	3.978473321	6.917266501
6.370431957	4.023848104	11.083618165
3.902563755	5.950519326	2.877973751
3.913687818	5.948417982	6.910430643
4.154618181	5.746641474	11.016321218
7.931766131	3.911467755	0.983871527
7.946570514	3.937787978	4.763738300
8.044502640	3.998620105	9.059453392
9.993433862	3.959466249	2.849102610
10.081251044	4.026638024	6.920813897
9.708446508	3.963194057	11.319429361
7.899889811	5.749431394	2.853711635
7.934817235	6.074700443	6.906935034
8.125920814	6.158944153	10.982711585
0.026142143	7.908805683	0.868361069
0.025192383	7.919419251	4.965784502
11.871477373	7.981509810	9.128070903
2.036392244	7.908746323	2.849335651
1.984618453	7.899035027	6.981378560
2.374257484	7.620138010	11.137787159
0.007871136	9.854341513	2.854255397
0.021440832	9.795848170	6.958359327
0.014424480	10.158300322	11.184032771
3.959656201	7.904353683	0.911344113
3.908155467	7.915026611	5.014049800
3.962232425	7.799654518	8.870353891
5.812115553	7.881606932	2.924789019
5.819820481	7.923313267	6.918794212
6.032341148	7.739902743	10.944441139
3.958480874	9.822880713	2.847108818

3.882001451	9.791823562	7.001730772
4.148539717	9.416727730	11.109925860
7.870827156	7.902335443	0.865202074
7.893110900	7.931504947	4.962625507
7.919561715	7.978434962	8.845185506
9.948985095	7.883114676	2.842344433
9.949448103	7.950986898	6.919726374
9.682589292	8.114333743	11.035793055
7.921342515	10.026307429	2.866632442
7.943650002	9.897068840	6.880290725
7.720717591	9.866225384	11.010961285

Atomic coordinates of $Ba_{0.5}Sr_{0.5}Co_{0.8}Fe_{0.2}O_{3-\delta}$ for optimization calculation.

1.0

11.8719997406	0.0000000000	0.0000000000
0.0000000000	11.8719997406	0.0000000000
0.0000000000	0.0000000000	25.8934001923

Sr	Ba	Co	Fe	O
13	14	22	5	81

Cartesian

1.991148052	1.965373941	1.510025419
1.961847957	2.013871060	5.068296473
5.929541502	1.954238005	4.988467121
5.961026046	1.897549207	8.947568010
5.956324734	5.991750781	8.662507567
9.929218215	5.961762110	1.460543131
9.858342377	5.991620189	4.957990589
9.816529194	5.999538813	8.553496353
1.931099478	9.886918280	4.969720299
5.919545279	9.923828327	1.448166086
9.903657800	9.874476424	1.426001335
9.863328616	9.860681161	5.016639140
9.892961128	9.804206058	8.691844790
1.928262070	2.066760819	8.982213379
5.893011359	1.925923286	1.175612156
9.948830759	1.970775701	1.208030693
9.894979368	1.986874133	4.989994831
9.984363654	1.953418837	8.941949142
1.919987286	5.891527359	1.209739657
1.973185717	5.898531839	4.992247557
1.976592981	5.987476861	8.914424458
5.942007102	5.933043742	1.185270394
5.936189822	5.914891455	4.956618238
1.959865333	9.925941543	1.215695139
1.957847093	9.802389642	8.731150971
5.958711006	9.926095879	5.013531932
5.893794911	9.924398183	8.747101306
11.854987165	11.823217694	3.091905024
11.843388221	11.826256926	6.837048747
11.838033949	11.362738440	10.651716250
3.906612107	11.870408893	3.100708780
3.886584043	0.052296159	6.823610072
3.545952627	11.706634656	10.655056499
7.965185810	0.021559552	6.860534061
7.999104113	11.384369223	10.673751534
11.867678333	3.922318762	3.090221953

11.744328255	3.976051433	6.845723036
11.406439271	4.361499649	10.696175218
3.967942857	3.923589066	2.998714676
7.918113331	3.893137387	3.032971645
7.865603476	3.893315467	6.846240904
7.871860020	4.135029382	10.613135084
11.852031037	7.930234643	3.042319162
11.463614822	7.834629429	10.628671124
3.876231659	7.927812755	3.043846873
3.956901898	7.969815890	6.807374911
4.114918214	8.115271631	10.676185513
7.904021267	7.924037459	6.824516341
7.799915702	7.759135382	10.596718668
7.926174419	0.000237440	3.011272975
3.964903625	3.931531434	6.826484240
4.217848452	4.312598882	10.603088445
11.845038429	7.892564788	6.800435479
7.922185427	7.923230163	2.990843083
0.004926880	11.864627229	1.228926667
11.838781885	11.857325949	4.964852340
11.862240957	11.809659870	8.791042406
1.964436053	0.020051808	3.141879286
1.922005526	0.012952352	6.825552077
1.740767578	11.727208832	10.876678111
0.031567647	1.980866901	3.087736186
0.022307488	2.084236402	6.970710479
11.730010624	2.783699011	11.132142397
3.924396362	11.862573373	1.226751621
3.974804873	0.040578495	4.963557670
3.963407753	0.000997248	8.766029381
5.994350749	0.001151584	3.030019797
6.070367163	0.084018142	6.937489247
6.423274228	11.836775517	11.009666615
3.963621449	1.952860853	3.093510414
3.993158985	1.978136341	6.860197447
3.977701641	1.409206369	10.976523062
7.925568947	11.756105279	1.145990106
7.938117651	0.045552863	4.901750123
8.113205903	11.866740445	8.936563315
9.867008936	11.824428638	3.036855655
9.898137320	11.844884093	6.910378857
10.133024835	11.633313186	10.893819542
7.919205555	1.929271190	2.965985418
7.934662899	1.966489909	7.040182472

8.188201325	2.490876138	10.831830742
11.864247325	3.954444394	1.227424849
0.017879232	3.937942314	4.969901553
11.809458046	4.156731397	8.848033780
1.970051509	3.908001131	3.063758898
2.093116658	3.912987371	6.941062536
2.734750756	3.766546254	11.059174796
11.826589342	5.997579933	3.087321892
11.770494143	5.975118109	6.815557225
0.016430848	6.102077275	10.896745496
3.887984939	3.868372395	1.119475264
3.967978473	3.918828394	4.929223021
4.130969158	3.828292524	8.881410373
5.929624606	3.911123467	2.977922276
5.934373406	3.859646476	6.845930183
5.823477057	3.696762639	10.840505031
3.947902922	5.928045630	2.950967246
3.995818313	5.784952418	6.996034224
4.128523526	5.997413725	10.612280602
7.972629554	4.004710440	1.190966942
7.872251796	3.891558411	4.912159270
7.753923575	4.144099589	8.729157179
9.891500872	4.035743848	3.089781765
9.841044873	4.007334152	6.914910202
9.666538349	4.614586939	10.726341030
7.969602194	6.038657052	3.043458472
7.954227954	5.985185565	6.805898987
7.550995483	5.920661247	10.760882826
11.759678751	7.931196275	1.173307643
11.848564413	7.963084466	4.954236046
11.781665695	7.847284981	8.751684438
1.932630966	7.968486226	3.024996477
1.944265526	7.886035188	6.868379761
2.460911210	7.946523026	10.947574241
11.844397341	9.877159496	3.095348846
11.818528254	9.827914441	6.845748930
0.045647839	9.537952720	10.831675382
4.001303177	7.987564529	1.198501921
3.913782794	7.869901140	4.924717569
4.039447912	7.914967251	8.751114783
6.052191132	7.939067411	3.013577488
5.897130943	7.883007828	6.886064954
5.910534431	8.079477551	10.856895553
3.937586154	9.902518088	3.087943333

3.898159243	9.900511720	6.808229393
3.933169770	10.011847333	10.792006693
7.989891441	7.965838770	1.141588228
7.959249810	7.963998610	4.980103552
7.886331988	7.875575956	8.685293759
9.870321224	7.943293842	3.117151089
9.905058696	7.957718322	6.869415497
9.710286668	7.737611447	10.728645542
7.955806930	9.868599784	3.059072192
8.000528753	9.936602599	6.896059806
8.053513488	9.668829645	10.804383738

Atomic coordinates of $BaCo_{0.4}Fe_{0.4}Zr_{0.1}Y_{0.1}O_{3-\delta}$ for optimization calculation.

1.0

11.8719997406	0.0000000000	0.0000000000
0.0000000000	11.8719997406	0.0000000000
0.0000000000	0.0000000000	25.8934001923

Ba	Y	Fe	Zr	Co	O
27	3	10	3	11	81

Cartesian

1.963165749	2.000443828	0.822866365
1.905883350	2.027322036	4.941625960
1.909729878	2.018251828	9.085372686
5.993258525	1.973055125	0.923177397
6.028375900	1.961384949	4.906902910
6.024458140	1.957728373	9.092804092
9.851634697	2.006427316	0.938195569
9.863257384	2.010487540	4.979585684
9.850886761	2.013977908	9.026775921
2.000147028	5.950922974	0.922348808
1.921720598	5.989530717	4.894914266
1.908162774	5.930253822	9.004248663
5.975723581	5.956039806	0.918257651
5.980223069	5.988296029	4.922024656
5.978644093	6.014165117	9.043710205
9.824614025	5.949854494	0.978226766
9.889530120	5.961370334	4.939891102
9.875271848	5.939205310	8.965175522
2.038683539	9.868837224	0.943426036
1.964234229	9.819710889	4.917337950
1.954285493	9.883142984	9.020276678
5.969680734	9.864325864	0.985891212
5.974346429	9.844760809	4.918684407
5.911911583	9.774858474	9.008857688
9.822298985	9.876067272	0.958444208
9.870713000	9.845793673	4.954236046
9.901271528	9.821574793	8.962275461
0.006731424	0.022319360	2.839366691
3.956949386	3.958385898	2.779060962
3.970020457	4.008794408	6.993652032
11.835920733	11.865956893	6.914728948
4.002514121	11.811511902	2.813550971
3.995355305	0.122898941	10.960287900
7.896957427	0.022817984	2.905912730
11.830471486	4.092693831	6.946085855
7.948018898	3.934083914	6.853827670

7.913174579	4.065815623	10.869661000
11.853989917	7.863585236	2.656248565
3.847299596	7.913471379	6.891865075
7.912580979	7.868262804	6.922419288
11.786343262	11.853348829	11.051536243
3.967764777	11.842533437	6.919596907
3.945113002	4.040635112	11.190842736
7.956958514	11.733132960	6.979048154
7.871373268	11.531534532	10.814948245
11.785666558	4.001599977	2.752960415
11.787257406	4.025070920	10.943845591
7.991767217	4.044564744	2.851044615
11.802679134	7.958715570	6.973455179
11.854524157	7.836730773	10.875176294
4.035435176	7.951390546	2.827818235
3.672781200	7.909173715	10.898868755
7.849077653	7.913851283	2.885223903
8.025661777	7.833928981	10.882555913
0.009010848	11.863261949	0.649043969
11.869079229	11.866657341	5.078161859
11.866028125	0.006315904	9.095341645
2.175733904	11.833332637	2.810573230
1.877580503	11.839791005	6.968613114
2.176944848	11.823550110	11.132375438
11.816984894	2.172196049	2.821914540
11.847104157	1.782200857	7.002145067
11.836846749	2.112669842	11.076678734
4.036800456	11.789513086	0.927113194
3.953019754	0.005852896	4.818502842
3.955869034	11.813826942	9.051581799
5.958770366	11.810063518	2.895762517
6.058851324	11.870195197	6.933760597
5.936094846	11.803118398	10.991618915
4.016321256	1.767147161	2.768884856
3.942382442	1.916176374	7.056857821
4.029689128	1.911332598	11.255886957
7.876751284	11.834875997	0.996248572
7.926625555	11.861279325	4.827643212
7.927919603	11.871358653	8.953186878
9.718573324	11.825247806	2.861013574
9.861262889	0.002706816	6.965997880
9.725945835	0.004903136	11.092888003
7.919383635	1.881296439	2.863085046
7.946439922	2.115732818	6.958100393

7.973021330	2.336599501	11.048662075
11.789109438	4.018968712	0.896843809
11.838390109	3.935366090	5.097115828
11.832418493	3.958172202	8.872813764
1.744519130	3.996055753	2.787217383
1.775528793	3.937040042	6.984770595
1.847663064	4.040599496	11.138175560
11.846000061	6.001438333	2.906093984
11.855283965	5.920364447	6.969830103
11.844207389	5.987073213	10.949956434
3.933229130	3.926212778	0.558261708
3.938120394	3.951120234	4.896933951
3.981583785	3.993610121	9.259790630
6.164725817	3.967135561	2.802080195
6.140305114	3.991686857	6.970373865
6.099774107	4.084537767	11.164172533
3.956818794	6.129204794	2.790946033
3.987507913	6.152794458	6.971124773
3.982201129	6.141326106	11.185482802
7.987232113	3.981192009	0.908340479
7.930662035	3.978556425	4.888855210
7.884313748	4.026246248	9.047723682
9.878809704	4.009839144	2.893458004
9.881243464	3.961080841	6.964884464
9.934596231	4.085297575	11.051277309
7.922363507	6.018771452	2.877300523
7.941881074	5.958212382	6.917887943
7.969483474	5.943645438	10.996176153
11.854464797	7.873854516	0.852100014
0.016324000	7.933143283	5.198876891
11.849371709	7.906051379	8.847153404
1.863132279	7.902050515	2.876316574
2.021932148	7.925141555	6.978918687
1.882198711	7.865924020	11.005109376
0.006577088	9.693060396	2.841981925
11.845608285	9.994597318	7.003336163
11.832335389	9.769136171	11.071733095
3.972335497	7.960365778	0.937703595
3.955726570	7.910455891	4.964489832
3.947914794	7.881120180	8.961136152
5.908266879	7.914243059	2.860340346
6.076730555	7.919597331	6.923739851
6.320557686	7.879731156	11.067046389
3.989680489	9.894278920	2.891490106

3.971789385	9.753951883	6.948131434
3.981951817	9.576607951	11.113110748
7.913578227	7.921318771	0.956087909
7.928062067	7.914824787	4.901465296
7.948054514	7.919015603	8.856397348
10.021060005	7.904531763	2.897316121
9.827273353	7.920998227	6.936660658
9.839335305	7.891389460	11.003245051
7.909280563	9.798080106	2.887269482
7.932062931	9.850020105	6.946137642
7.946831698	9.763152683	10.964586205

Atomic coordinates of $BaCo_{0.8}(Zr_{1/6}Ti_{1/6}Zn_{1/6}In_{1/6}Cu_{1/6}Mo_{1/6})_{0.2}O_{3-\delta}$ for hydration calculation:

```
1.0
    11.8719997406     0.0000000000     0.0000000000
     0.0000000000    11.8719997406     0.0000000000
     0.0000000000     0.0000000000    25.8934001923
  Ba  Co  In  Zr  Zn  Ti  Mo  Cu  O  H
  27  21   1   1   1   1   1   1  81  2
Direct
   0.165720000    0.165280000    0.039040000
   0.165420000    0.167620000    0.189200000
   0.159410000    0.167170000    0.347250000
   0.500270000    0.167550000    0.041060000
   0.500900000    0.170220000    0.189770000
   0.511780000    0.174670000    0.344490000
   0.835700000    0.166530000    0.038540000
   0.833760000    0.165970000    0.189560000
   0.829190000    0.164930000    0.346160000
   0.167720000    0.497810000    0.036750000
   0.163680000    0.499120000    0.188290000
   0.156580000    0.500480000    0.348390000
   0.502330000    0.498170000    0.040150000
   0.502890000    0.496290000    0.188100000
   0.504930000    0.496900000    0.343170000
   0.829240000    0.499010000    0.037200000
   0.832690000    0.502720000    0.188760000
   0.832600000    0.502810000    0.341340000
   0.168380000    0.836840000    0.038140000
   0.164400000    0.832170000    0.187210000
   0.166040000    0.835460000    0.359930000
   0.502030000    0.834010000    0.040500000
   0.503090000    0.834010000    0.187430000
   0.498670000    0.833110000    0.346170000
   0.827800000    0.834320000    0.038180000
   0.831320000    0.830060000    0.187140000
   0.830690000    0.833490000    0.343590000
   0.999150000    0.001430000    0.110290000
   0.995760000    0.987500000    0.260230000
   0.966140000    0.996290000    0.422810000
   0.332070000    0.002700000    0.110790000
   0.353090000    0.038040000    0.420490000
   0.667080000    0.001920000    0.112240000
   0.668690000    0.997560000    0.264590000
   0.661200000    0.987210000    0.418830000
   0.004320000    0.333470000    0.109520000
```

0.000530000	0.328030000	0.258400000
0.329040000	0.333810000	0.108010000
0.333100000	0.334720000	0.266830000
0.318480000	0.340520000	0.417400000
0.682630000	0.329710000	0.111220000
0.674160000	0.367190000	0.420650000
0.997030000	0.667850000	0.264930000
0.961450000	0.655490000	0.420330000
0.333040000	0.665620000	0.105830000
0.342290000	0.651090000	0.422610000
0.674610000	0.669910000	0.265160000
0.657150000	0.672660000	0.417790000
0.330700000	0.003090000	0.267360000
0.991620000	0.330490000	0.434070000
0.671290000	0.334630000	0.264470000
0.998310000	0.666260000	0.105780000
0.329950000	0.662230000	0.266910000
0.665110000	0.665630000	0.110200000
0.999230000	0.999330000	0.037140000
0.000130000	0.999470000	0.186440000
0.003560000	0.000750000	0.355060000
0.163770000	0.999040000	0.110290000
0.148090000	0.000640000	0.270180000
0.223220000	0.009040000	0.438930000
0.001050000	0.168860000	0.110190000
0.997220000	0.175630000	0.268670000
0.987930000	0.145630000	0.435730000
0.333800000	0.000980000	0.038510000
0.330620000	0.997460000	0.183750000
0.334100000	0.002500000	0.351200000
0.499510000	0.004640000	0.111940000
0.511090000	0.999030000	0.266070000
0.494990000	0.997250000	0.425940000
0.327640000	0.168450000	0.111850000
0.327180000	0.180540000	0.269680000
0.351380000	0.193120000	0.421740000
0.666490000	0.004850000	0.039170000
0.669600000	0.002480000	0.186980000
0.666240000	0.994730000	0.344800000
0.836610000	0.001850000	0.110980000
0.840150000	0.995780000	0.266260000
0.816900000	0.979530000	0.426000000
0.669620000	0.171630000	0.111640000
0.673720000	0.156470000	0.268100000

0.664780000	0.126350000	0.430200000
0.003800000	0.333550000	0.035810000
0.001000000	0.334520000	0.185250000
0.990920000	0.331690000	0.361070000
0.167910000	0.333820000	0.111140000
0.151570000	0.339590000	0.269700000
0.170030000	0.334170000	0.431800000
0.003080000	0.499730000	0.109570000
0.001160000	0.512120000	0.268530000
0.990780000	0.511610000	0.435950000
0.330300000	0.332010000	0.036040000
0.325950000	0.336420000	0.195730000
0.328560000	0.341160000	0.342420000
0.479280000	0.332200000	0.112060000
0.490800000	0.335110000	0.267140000
0.536590000	0.338940000	0.428050000
0.328720000	0.501220000	0.111150000
0.329660000	0.501050000	0.266880000
0.349950000	0.484050000	0.425450000
0.668110000	0.329470000	0.038000000
0.669350000	0.331690000	0.183970000
0.677600000	0.336810000	0.349870000
0.841760000	0.333510000	0.110030000
0.849160000	0.339170000	0.267280000
0.817760000	0.333830000	0.437160000
0.665420000	0.484280000	0.110210000
0.668360000	0.511680000	0.266750000
0.684460000	0.518780000	0.424150000
0.002200000	0.666170000	0.033540000
0.002120000	0.667070000	0.191780000
0.999960000	0.672300000	0.352520000
0.171530000	0.666170000	0.110040000
0.167170000	0.665350000	0.269620000
0.199990000	0.642700000	0.430140000
0.000660000	0.830050000	0.110230000
0.001810000	0.825120000	0.268730000
0.001210000	0.855650000	0.431930000
0.333530000	0.665800000	0.035200000
0.329190000	0.666700000	0.193640000
0.333750000	0.656980000	0.342570000
0.489560000	0.663880000	0.112950000
0.490210000	0.667390000	0.267200000
0.508110000	0.651950000	0.422670000
0.333430000	0.827400000	0.109950000

0.326990000	0.824780000	0.270410000
0.348600000	0.793190000	0.429060000
0.662970000	0.665630000	0.033410000
0.664850000	0.668090000	0.191660000
0.667080000	0.672040000	0.341600000
0.838020000	0.664010000	0.109770000
0.838060000	0.669730000	0.267240000
0.815580000	0.683490000	0.426200000
0.667230000	0.844530000	0.110710000
0.669110000	0.833650000	0.265720000
0.650330000	0.831050000	0.425240000
0.504490000	0.735270000	0.422670000
0.567790000	0.831900000	0.425240000

Atomic coordinates of $Ba_{0.5}Sr_{0.5}Co_{0.8}Fe_{0.2}O_{3-\delta}$ for hydration calculation:
1.0

11.8719997406	0.0000000000	0.0000000000
0.0000000000	11.8719997406	0.0000000000
0.0000000000	0.0000000000	25.8934001923

Sr Ba Co Fe O H
13 14 22 5 81 2
Direct

0.167720000	0.165550000	0.058320000
0.165250000	0.169630000	0.195740000
0.499460000	0.164610000	0.192650000
0.502110000	0.159830000	0.345550000
0.501710000	0.504700000	0.334540000
0.836360000	0.502170000	0.056410000
0.830390000	0.504690000	0.191480000
0.826860000	0.505350000	0.330330000
0.162660000	0.832790000	0.191930000
0.498610000	0.835900000	0.055930000
0.834200000	0.831740000	0.055070000
0.830810000	0.830580000	0.193740000
0.833300000	0.825830000	0.335680000
0.162420000	0.174090000	0.346890000
0.496380000	0.162220000	0.045400000
0.838010000	0.166000000	0.046650000
0.833470000	0.167360000	0.192710000
0.841000000	0.164540000	0.345340000
0.161720000	0.496250000	0.046720000
0.166200000	0.496840000	0.192800000
0.166490000	0.504340000	0.344270000
0.500510000	0.499750000	0.045780000
0.500020000	0.498220000	0.191420000
0.165080000	0.836080000	0.046950000
0.164910000	0.825670000	0.337200000
0.501910000	0.836090000	0.193620000
0.496450000	0.835950000	0.337810000
0.998570000	0.995890000	0.119410000
0.997590000	0.996150000	0.264050000
0.997140000	0.957100000	0.411370000
0.329060000	0.999870000	0.119750000
0.327370000	0.004400000	0.263530000
0.298680000	0.986070000	0.411500000
0.670920000	0.001820000	0.264950000
0.673780000	0.958930000	0.412220000
0.999640000	0.330380000	0.119340000

0.989250000	0.334910000	0.264380000
0.960790000	0.367380000	0.413080000
0.334230000	0.330490000	0.115810000
0.666960000	0.327930000	0.117130000
0.662530000	0.327940000	0.264400000
0.663060000	0.348300000	0.409880000
0.998320000	0.667980000	0.117490000
0.965600000	0.659920000	0.410480000
0.326500000	0.667770000	0.117550000
0.333300000	0.671310000	0.262900000
0.346610000	0.683560000	0.412310000
0.665770000	0.667460000	0.263560000
0.657000000	0.653570000	0.409240000
0.667640000	0.000020000	0.116290000
0.333970000	0.331160000	0.263640000
0.355280000	0.363260000	0.409490000
0.997730000	0.664800000	0.262630000
0.667300000	0.667390000	0.115510000
0.000410000	0.999380000	0.047460000
0.997200000	0.998760000	0.191740000
0.999180000	0.994750000	0.339510000
0.165470000	0.001690000	0.121340000
0.161890000	0.001090000	0.263600000
0.146630000	0.987800000	0.420060000
0.002660000	0.166850000	0.119250000
0.001880000	0.175560000	0.269210000
0.988040000	0.234480000	0.429920000
0.330560000	0.999210000	0.047380000
0.334810000	0.003420000	0.191690000
0.333850000	0.000080000	0.338540000
0.504920000	0.000100000	0.117020000
0.511320000	0.007080000	0.267930000
0.541040000	0.997030000	0.425190000
0.333860000	0.164490000	0.119470000
0.336350000	0.166620000	0.264940000
0.335050000	0.118700000	0.423910000
0.667580000	0.990240000	0.044260000
0.668640000	0.003840000	0.189300000
0.683390000	0.999560000	0.345130000
0.831120000	0.995990000	0.117280000
0.833740000	0.997720000	0.266880000
0.853520000	0.979890000	0.420720000
0.667050000	0.162510000	0.114550000
0.668350000	0.165640000	0.271890000

0.689710000	0.209810000	0.418320000
0.999350000	0.333090000	0.047400000
0.001510000	0.331700000	0.191940000
0.994730000	0.350130000	0.341710000
0.165940000	0.329180000	0.118320000
0.176310000	0.329600000	0.268060000
0.230350000	0.317260000	0.427100000
0.996180000	0.505190000	0.119230000
0.991450000	0.503300000	0.263220000
0.001380000	0.513990000	0.420830000
0.327490000	0.325840000	0.043230000
0.334230000	0.330090000	0.190370000
0.347960000	0.322460000	0.343000000
0.499460000	0.329440000	0.115010000
0.499860000	0.325100000	0.264390000
0.490520000	0.311390000	0.418660000
0.332540000	0.499330000	0.113970000
0.336580000	0.487280000	0.270190000
0.347750000	0.505170000	0.409850000
0.671550000	0.337320000	0.046000000
0.663090000	0.327790000	0.189710000
0.653130000	0.349070000	0.337120000
0.833180000	0.339940000	0.119330000
0.828930000	0.337540000	0.267050000
0.814230000	0.388690000	0.414250000
0.671290000	0.508650000	0.117540000
0.670000000	0.504140000	0.262840000
0.636030000	0.498710000	0.415580000
0.990540000	0.668060000	0.045310000
0.998030000	0.670740000	0.191330000
0.992390000	0.660990000	0.337990000
0.162790000	0.671200000	0.116820000
0.163770000	0.664260000	0.265260000
0.207290000	0.669350000	0.422790000
0.997680000	0.831970000	0.119540000
0.995500000	0.827820000	0.264380000
0.003840000	0.803400000	0.418320000
0.337040000	0.672810000	0.046290000
0.329660000	0.662900000	0.190190000
0.340250000	0.666690000	0.337970000
0.509790000	0.668720000	0.116380000
0.496730000	0.664000000	0.265940000
0.497850000	0.680550000	0.419290000
0.331670000	0.834110000	0.119260000

0.328350000	0.833940000	0.262930000
0.331300000	0.843320000	0.416790000
0.673000000	0.670980000	0.044090000
0.670420000	0.670820000	0.192330000
0.664280000	0.663370000	0.335420000
0.831390000	0.669080000	0.120380000
0.834320000	0.670290000	0.265300000
0.817910000	0.651750000	0.414340000
0.670130000	0.831250000	0.118140000
0.673900000	0.836980000	0.266320000
0.678360000	0.814420000	0.417260000
0.824180000	0.735120000	0.422790000
0.761430000	0.815280000	0.409850000

Atomic coordinates of $BaCo_{0.4}Fe_{0.4}Zr_{0.1}Y_{0.1}O_{3-\delta}$for hydration calculation:

1.0

11.8719997406	0.0000000000	0.0000000000
0.0000000000	11.8719997406	0.0000000000
0.0000000000	0.0000000000	25.8934001923

Ba	Y	Fe	Zr	Co	O	H
27	3	10	3	11	81	2

Cartesian

1.963153877	2.000431956	0.822892258
1.905930838	2.027262676	4.941496493
1.909729878	2.018239956	9.085476259
5.993222909	1.973007637	0.923099717
6.028364028	1.961373077	4.906799336
6.024446268	1.957692757	9.092726412
9.851622825	2.006367956	0.938117889
9.863257384	2.010523156	4.979559791
9.850910505	2.013966036	9.026698241
2.000194516	5.950958590	0.922322915
1.921720598	5.989542589	4.894888372
1.908186518	5.930301310	9.004170983
5.975771069	5.956063550	0.918179971
5.980163709	5.988236669	4.922076443
5.978620349	6.014117629	9.043787885
9.824554665	5.949890110	0.978252659
9.889494504	5.961405950	4.939942889
9.875248104	5.939205310	8.965071949
2.038659795	9.868837224	0.943555503
1.964222357	9.819687145	4.917415631
1.954249877	9.883083624	9.020224891
5.969716350	9.864325864	0.985761745
5.974346429	9.844737065	4.918710301
5.911899711	9.774810986	9.008831795
9.822298985	9.876079144	0.958314741
9.870736744	9.845805545	4.954184259
9.901247784	9.821586665	8.962223675
0.006767040	0.022319360	2.839470265
3.956937514	3.958362154	2.779138643
3.969996713	4.008818152	6.993548458
11.835908861	11.865945021	6.914832521
4.002525993	11.811571262	2.813576865
3.995402793	0.122875197	10.960417367
7.896898067	0.022794240	2.906016304
11.830447742	4.092753191	6.946163536
7.948066386	3.934024554	6.853724097

7.913162707	4.065803751	10.869790467
11.853954301	7.863537748	2.656144992
3.847240236	7.913518867	6.891787395
7.912569107	7.868286548	6.922341607
11.786402622	11.853360701	11.051562136
3.967741033	11.842557181	6.919493333
3.945065514	4.040635112	11.190868629
7.956970386	11.733097344	6.979048154
7.871373268	11.531510788	10.814896458
11.785690302	4.001576233	2.752986308
11.787233662	4.025082792	10.943845591
7.991755345	4.044552872	2.851122295
11.802667262	7.958751186	6.973351606
11.854547901	7.836707029	10.875228081
4.035411432	7.951390546	2.827818235
3.672840560	7.909126227	10.898791075
7.849053909	7.913875027	2.885301583
8.025709265	7.833976469	10.882478233
0.009022720	11.863214461	0.649147543
11.869031741	11.866657341	5.078213646
11.866063741	0.006292160	9.095315752
2.175781392	11.833297021	2.810469657
1.877556759	11.839826621	6.968690794
2.176968592	11.823561982	11.132349545
11.817032382	2.172219793	2.821862753
11.847068541	1.782224601	7.002093280
11.836858621	2.112622354	11.076678734
4.036836072	11.789489342	0.926983727
3.953019754	0.005817280	4.818502842
3.955869034	11.813826942	9.051555905
5.958794110	11.810027902	2.895658944
6.058875068	11.870218941	6.933734703
5.936118590	11.803142142	10.991748382
4.016297512	1.767147161	2.768781283
3.942334954	1.916140758	7.056987288
4.029712872	1.911273238	11.255861064
7.876715668	11.834840381	0.996119105
7.926578067	11.861314941	4.827565532
7.927883987	11.871406141	8.953160984
9.718537708	11.825224062	2.860961787
9.861239145	0.002730560	6.966101454
9.725898347	0.004867520	11.092991576
7.919336147	1.881237079	2.863033259
7.946404306	2.115709074	6.958074500

7.972997586	2.336646989	11.048713862
11.789133182	4.019028072	0.896947383
11.838401981	3.935330474	5.097115828
11.832465981	3.958124714	8.872891444
1.744471642	3.995996393	2.787165597
1.775576281	3.936992554	6.984744702
1.847639320	4.040635112	11.138305027
11.846000061	6.001414589	2.906016304
11.855260221	5.920328831	6.969726530
11.844219261	5.987049469	10.950060007
3.933193514	3.926189034	0.558261708
3.938061034	3.951120234	4.896959844
3.981631273	3.993621993	9.259738843
6.164773305	3.967147433	2.802183769
6.140316986	3.991722473	6.970244398
6.099833467	4.084561511	11.164198427
3.956818794	6.129157306	2.791049607
3.987448553	6.152782586	6.971021200
3.982224873	6.141266746	11.185431015
7.987243985	3.981156393	0.908340479
7.930614547	3.978544553	4.888932890
7.884313748	4.026269992	9.047671895
9.878809704	4.009886632	2.893328537
9.881302824	3.961092713	6.964806784
9.934608103	4.085273831	11.051303202
7.922304147	6.018747708	2.877274629
7.941892946	5.958200510	6.917939729
7.969435986	5.943597950	10.996150260
11.854429181	7.873866388	0.852151800
0.016264640	7.933107667	5.198876891
11.849324221	7.906039507	8.847256978
1.863072919	7.902003027	2.876238893
2.021920276	7.925153427	6.979048154
1.882186839	7.865912148	11.005212950
0.006529600	9.693012908	2.842059605
11.845643901	9.994561702	7.003387950
11.832347261	9.769112427	11.071758988
3.972371113	7.960413266	0.937600021
3.955750314	7.910432147	4.964541619
3.947914794	7.881108308	8.961187939
5.908219391	7.914231187	2.860443919
6.076683067	7.919573587	6.923636277
6.320533942	7.879683668	11.067098176
3.989704233	9.894243304	2.891515999

3.971777513	9.753916267	6.948235008
3.981987433	9.576548591	11.113188429
7.913637587	7.921354387	0.955984335
7.928121427	7.914824787	4.901361722
7.948066386	7.918979987	8.856319668
10.021036261	7.904496147	2.897212548
9.827285225	7.920998227	6.936582978
9.839275945	7.891436948	11.003141478
7.909244947	9.798080106	2.887373055
7.932039187	9.850079465	6.946163536
7.946879186	9.763176427	10.964560311
6.345583861	8.871470526	11.067098176
6.980498407	9.806984106	11.113188429

Atomic coordinates of $BaCo_{0.4}Fe_{0.4}Zr_{0.1}Y_{0.1}O_{3-\delta}$ for proton diffusion calculation:

1.0

11.8719997406	0.0000000000	0.0000000000
0.0000000000	11.8719997406	0.0000000000
0.0000000000	0.0000000000	25.8934001923

Ba	Y	Fe	Zr	Co	O	H
27	3	10	3	11	81	16

Cartesian

1.963153877	2.000431956	0.822892258
1.905930838	2.027262676	4.941496493
1.909729878	2.018239956	9.085476259
5.993222909	1.973007637	0.923099717
6.028364028	1.961373077	4.906799336
6.024446268	1.957692757	9.092726412
9.851622825	2.006367956	0.938117889
9.863257384	2.010523156	4.979559791
9.850910505	2.013966036	9.026698241
2.000194516	5.950958590	0.922322915
1.921720598	5.989542589	4.894888372
1.908186518	5.930301310	9.004170983
5.975771069	5.956063550	0.918179971
5.980163709	5.988236669	4.922076443
5.978620349	6.014117629	9.043787885
9.824554665	5.949890110	0.978252659
9.889494504	5.961405950	4.939942889
9.875248104	5.939205310	8.965071949
2.038659795	9.868837224	0.943555503
1.964222357	9.819687145	4.917415631
1.954249877	9.883083624	9.020224891
5.969716350	9.864325864	0.985761745
5.974346429	9.844737065	4.918710301
5.911899711	9.774810986	9.008831795
9.822298985	9.876079144	0.958314741
9.870736744	9.845805545	4.954184259
9.901247784	9.821586665	8.962223675
0.006767040	0.022319360	2.839470265
3.956937514	3.958362154	2.779138643
3.969996713	4.008818152	6.993548458
11.835908861	11.865945021	6.914832521
4.002525993	11.811571262	2.813576865
3.995402793	0.122875197	10.960417367
7.896898067	0.022794240	2.906016304
11.830447742	4.092753191	6.946163536
7.948066386	3.934024554	6.853724097

7.913162707	4.065803751	10.869790467
11.853954301	7.863537748	2.656144992
3.847240236	7.913518867	6.891787395
7.912569107	7.868286548	6.922341607
11.786402622	11.853360701	11.051562136
3.967741033	11.842557181	6.919493333
3.945065514	4.040635112	11.190868629
7.956970386	11.733097344	6.979048154
7.871373268	11.531510788	10.814896458
11.785690302	4.001576233	2.752986308
11.787233662	4.025082792	10.943845591
7.991755345	4.044552872	2.851122295
11.802667262	7.958751186	6.973351606
11.854547901	7.836707029	10.875228081
4.035411432	7.951390546	2.827818235
3.672840560	7.909126227	10.898791075
7.849053909	7.913875027	2.885301583
8.025709265	7.833976469	10.882478233
0.009022720	11.863214461	0.649147543
11.869031741	11.866657341	5.078213646
11.866063741	0.006292160	9.095315752
2.175781392	11.833297021	2.810469657
1.877556759	11.839826621	6.968690794
2.176968592	11.823561982	11.132349545
11.817032382	2.172219793	2.821862753
11.847068541	1.782224601	7.002093280
11.836858621	2.112622354	11.076678734
4.036836072	11.789489342	0.926983727
3.953019754	0.005817280	4.818502842
3.955869034	11.813826942	9.051555905
5.958794110	11.810027902	2.895658944
6.058875068	11.870218941	6.933734703
5.936118590	11.803142142	10.991748382
4.016297512	1.767147161	2.768781283
3.942334954	1.916140758	7.056987288
4.029712872	1.911273238	11.255861064
7.876715668	11.834840381	0.996119105
7.926578067	11.861314941	4.827565532
7.927883987	11.871406141	8.953160984
9.718537708	11.825224062	2.860961787
9.861239145	0.002730560	6.966101454
9.725898347	0.004867520	11.092991576
7.919336147	1.881237079	2.863033259
7.946404306	2.115709074	6.958074500

7.972997586	2.336646989	11.048713862
11.789133182	4.019028072	0.896947383
11.838401981	3.935330474	5.097115828
11.832465981	3.958124714	8.872891444
1.744471642	3.995996393	2.787165597
1.775576281	3.936992554	6.984744702
1.847639320	4.040635112	11.138305027
11.846000061	6.001414589	2.906016304
11.855260221	5.920328831	6.969726530
11.844219261	5.987049469	10.950060007
3.933193514	3.926189034	0.558261708
3.938061034	3.951120234	4.896959844
3.981631273	3.993621993	9.259738843
6.164773305	3.967147433	2.802183769
6.140316986	3.991722473	6.970244398
6.099833467	4.084561511	11.164198427
3.956818794	6.129157306	2.791049607
3.987448553	6.152782586	6.971021200
3.982224873	6.141266746	11.185431015
7.987243985	3.981156393	0.908340479
7.930614547	3.978544553	4.888932890
7.884313748	4.026269992	9.047671895
9.878809704	4.009886632	2.893328537
9.881302824	3.961092713	6.964806784
9.934608103	4.085273831	11.051303202
7.922304147	6.018747708	2.877274629
7.941892946	5.958200510	6.917939729
7.969435986	5.943597950	10.996150260
11.854429181	7.873866388	0.852151800
0.016264640	7.933107667	5.198876891
11.849324221	7.906039507	8.847256978
1.863072919	7.902003027	2.876238893
2.021920276	7.925153427	6.979048154
1.882186839	7.865912148	11.005212950
0.006529600	9.693012908	2.842059605
11.845643901	9.994561702	7.003387950
11.832347261	9.769112427	11.071758988
3.972371113	7.960413266	0.937600021
3.955750314	7.910432147	4.964541619
3.947914794	7.881108308	8.961187939
5.908219391	7.914231187	2.860443919
6.076683067	7.919573587	6.923636277
6.320533942	7.879683668	11.067098176
3.989704233	9.894243304	2.891515999

3.971777513	9.753916267	6.948235008
3.981987433	9.576548591	11.113188429
7.913637587	7.921354387	0.955984335
7.928121427	7.914824787	4.901361722
7.948066386	7.918979987	8.856319668
10.021036261	7.904496147	2.897212548
9.827285225	7.920998227	6.936582978
9.839275945	7.891436948	11.003141478
7.909244947	9.798080106	2.887373055
7.932039187	9.850079465	6.946163536
7.946879186	9.763176427	10.964560311
7.025968166	5.989661309	6.906805567
6.113130106	4.998942931	6.917162927
4.992294611	6.088317627	6.910430643
6.049377468	6.994032487	6.918716531
6.976461928	5.623172677	6.906546633
6.787815852	5.307140044	6.906287699
6.475938419	5.078604049	6.906805567
5.699509635	5.044175250	6.910430643
5.043462930	5.657126596	6.910430643
5.292893644	5.276272845	6.910430643
6.765852652	6.715396653	6.918457597
6.983347687	6.373126901	6.917680795
6.436285939	6.935859688	6.919234399
5.680158276	6.946900648	6.909912775
5.334208203	6.759679212	6.909394907
5.099142609	6.468221619	6.908618105

Atomic coordinates of $Ba_{0.5}Sr_{0.5}Co_{0.8}Fe_{0.2}O_{3-\delta}$ for proton diffusion calculation:

1.0

11.8719997406	0.0000000000	0.0000000000
0.0000000000	11.8719997406	0.0000000000
0.0000000000	0.0000000000	25.8934001923

Sr	Ba	Co	Fe	O	H
13	14	22	5	81	16

Cartesian

1.991171796	1.965409557	1.510103099
1.961847957	2.013847316	5.068374154
5.929588990	1.954249877	4.988363547
5.961049790	1.897501719	8.947464436
5.956300990	5.991798269	8.662378100
9.929265703	5.961762110	1.460646705
9.858389865	5.991679549	4.958068269
9.816481706	5.999515069	8.553366886
1.931099478	9.886882664	4.969720299
5.919497791	9.923804583	1.448217873
9.903622184	9.874417064	1.425949549
9.863376104	9.860645545	5.016587353
9.892937384	9.804253546	8.691896577
1.928250198	2.066796435	8.982161593
5.893023231	1.925875798	1.175560369
9.948854503	1.970751957	1.207927119
9.894955624	1.986897877	4.989917151
9.984351782	1.953418837	8.942026822
1.919939798	5.891479871	1.209739657
1.973126357	5.898484351	4.992247557
1.976569237	5.987524349	8.914320884
5.942054590	5.933031870	1.185399861
5.936237310	5.914867711	4.956514665
1.959829717	9.925941543	1.215695139
1.957811477	9.802354026	8.731254545
5.958675390	9.926060263	5.013480145
5.893854271	9.924398183	8.747049519
11.855022781	11.823205822	3.091930917
11.843388221	11.826292542	6.837152321
11.838045821	11.362690952	10.651768037
3.906600235	11.870456381	3.100734673
3.886536555	0.052236799	6.823687753
3.545928883	11.706622784	10.655134179
7.965162066	0.021607040	6.860456381
7.999115985	11.384416711	10.673777427
11.867725821	3.922271274	3.090118379

11.744375743	3.976051433	6.845697143
11.406498631	4.361535265	10.696045751
3.967978473	3.923577194	2.998714676
7.918148947	3.893184875	3.032893965
7.865555988	3.893303595	6.846215011
7.871848148	4.135017510	10.613186871
11.852054781	7.930258387	3.042215589
11.463602950	7.834570069	10.628722911
3.876207915	7.927765267	3.043769193
3.956937514	7.969792146	6.807374911
4.114953830	8.115224143	10.676107833
7.904021267	7.924084947	6.824464555
7.799903830	7.759182870	10.596615095
7.926221907	0.000237440	3.011143508
3.964891753	3.931531434	6.826536027
4.217884068	4.312622626	10.603088445
11.845050301	7.892505428	6.800383692
7.922185427	7.923253907	2.990946656
0.004867520	11.864639101	1.228900773
11.838758141	11.857278461	4.964800553
11.862264701	11.809671742	8.791068299
1.964459797	0.020063680	3.141905179
1.921958038	0.012940480	6.825500291
1.740791322	11.727161344	10.876781685
0.031579519	1.980843157	3.087787973
0.022319360	2.084248274	6.970762266
11.730010624	2.783746499	11.132090611
3.924408234	11.862620861	1.226829301
3.974864233	0.040602239	4.963505883
3.963467113	0.000949760	8.765951701
5.994410109	0.001187200	3.030045690
6.070390907	0.084053758	6.937618714
6.423226740	11.836739901	11.009614828
3.963585833	1.952825237	3.093484521
3.993147113	1.978112597	6.860197447
3.977713513	1.409206369	10.976471276
7.925509587	11.756129023	1.146041893
7.938093907	0.045588479	4.901620656
8.113205903	11.866776061	8.936589208
9.867056424	11.824393022	3.036777975
9.898161064	11.844931581	6.910430643
10.132989219	11.633253826	10.893871329
7.919217427	1.929318678	2.966088992
7.934651027	1.966478037	7.040156578

8.188236941	2.490864266	10.831727168
11.864282941	3.954444394	1.227347169
0.017926720	3.937942314	4.969979233
11.809434302	4.156743269	8.848033780
1.970039637	3.908024875	3.063707111
2.093152274	3.913011115	6.940984856
2.734715140	3.766510638	11.059071222
11.826648702	5.997615549	3.087270105
11.770494143	5.975177469	6.815660799
0.016383360	6.102089147	10.896719603
3.887961195	3.868372395	1.119371690
3.967978473	3.918828394	4.929326595
4.130981030	3.828245036	8.881436266
5.929588990	3.911111595	2.977999956
5.934337790	3.859587116	6.845956077
5.823453313	3.696821999	10.840530924
3.947914794	5.928045630	2.951070820
3.995877673	5.784988034	6.996137798
4.128487910	5.997378109	10.612410069
7.972641426	4.004662952	1.191096409
7.872204308	3.891522795	4.912236950
7.753959191	4.144158949	8.729183073
9.891512744	4.035767592	3.089859445
9.841056745	4.007274792	6.914832521
9.666538349	4.614527579	10.726341030
7.969554706	6.038692668	3.043510259
7.954239826	5.985149949	6.805821307
7.550947995	5.920684991	10.760779252
11.759690623	7.931208147	1.173229963
11.848611901	7.963025106	4.954184259
11.781653823	7.847273109	8.751710331
1.932642838	7.968486226	3.024867010
1.944277398	7.886094548	6.868483335
2.460946826	7.946523026	10.947470667
11.844456701	9.877147624	3.095297059
11.818575742	9.827878825	6.845697143
0.045588479	9.537964592	10.831727168
4.001338793	7.987600145	1.198605495
3.913723434	7.869948628	4.924665783
4.039447912	7.914943507	8.751192463
6.052226748	7.939043667	3.013473914
5.897178431	7.883007828	6.886090847
5.910475071	8.079489423	10.856843767
3.937586154	9.902553704	3.088046907

3.898171115	9.900535464	6.808151713
3.933193514	10.011894821	10.792110266
7.989855825	7.965874386	1.141640014
7.959226066	7.963974866	4.980077659
7.886331988	7.875528468	8.685164292
9.870261864	7.943317586	3.117047515
9.905046824	7.957682706	6.869519071
9.710227308	7.737575831	10.728671436
7.955783186	9.868599784	3.059046299
8.000540625	9.936626343	6.895930339
8.053489744	9.668794029	10.804280164
6.946188328	5.949296510	6.907064501
5.933388030	4.938158292	6.917162927
4.893519573	5.797809793	6.910430643
5.869635392	6.933247849	6.918716531
6.890864809	5.550872199	6.906546633
6.690346734	5.204922126	6.906287699
6.336679862	4.988139411	6.906805567
5.563575238	5.005116371	6.910430643
5.009152851	5.457320841	6.910430643
5.245999245	5.173223887	6.910430643
6.627068975	6.699013294	6.918457597
6.888609129	6.364341621	6.917680795
6.260699063	6.889915049	6.919234399
5.491037320	6.846107370	6.909912775
5.147699088	6.592165296	6.909394907
4.936140052	6.225795384	6.908618105

Atomic coordinates of $BaCo_{0.8}(Zr_{1/6}Ti_{1/6}Zn_{1/6}In_{1/6}Cu_{1/6}Mo_{1/6})_{0.2}O_{3-\delta}$ for proton diffusion calculation:

1.0

11.8719997406	0.0000000000	0.0000000000
0.0000000000	11.8719997406	0.0000000000
0.0000000000	0.0000000000	25.8934001923

Ba	Co	In	Zr	Zn	Ti	Mo	Cu	O	H
27	21	1	1	1	1	1	1	81	16

Cartesian

1.967427797	1.962204117	1.010878344
1.963866197	1.989984597	4.899031316
1.892515479	1.984642197	8.991483217
5.939205310	1.989153557	1.063183012
5.946684670	2.020851796	4.913790554
6.075852027	2.073682195	8.920017432
9.921430183	1.977044117	0.997931643
9.898398504	1.970395797	4.908352940
9.844143465	1.958048917	8.963259411
1.991171796	5.910000191	0.951582457
1.943208918	5.925552511	4.875468322
1.858917719	5.941698430	9.021001693
5.963661630	5.914274111	1.039620018
5.970309950	5.891954751	4.870548576
5.994528829	5.899196671	8.885838144
9.844737065	5.924246591	0.963234487
9.885695464	5.968291710	4.887638220
9.884626984	5.969360190	8.838453222
1.999007316	9.934964263	0.987574283
1.951756757	9.879522024	4.847503450
1.971226837	9.918580903	9.319811531
5.960100030	9.901366504	1.048682708
5.972684349	9.901366504	4.853199998
5.920210111	9.890681704	8.963518345
9.827641385	9.905046824	0.988610019
9.869430824	9.854472105	4.845690912
9.861951465	9.895193064	8.896713372
11.861908541	0.016976960	2.855783107
11.821662462	11.723599744	6.738239532
11.470013829	11.827954622	10.947988535
3.942334954	0.032054399	2.868729807
4.191884388	0.451610870	10.887915847
7.919573587	0.022794240	2.906275238
7.938687507	11.843032061	6.851134757
7.849766228	11.720156864	10.844932803
0.051287039	3.958955753	2.835845189

0.006292160	3.894372075	6.690854610
3.906362795	3.962992233	2.796746155
3.954563114	3.973795753	6.909135973
3.780994477	4.042653352	10.807905240
8.104183183	3.914317034	2.879863969
8.003627345	4.359279585	10.892058791
11.836739901	7.928715027	6.859938513
11.414334151	7.781977110	10.883772903
3.953850794	7.902240467	2.740298542
4.063666791	7.729740311	10.942809855
8.008969745	7.953171346	6.865893995
7.801684630	7.985819346	10.818003666
3.926070314	0.036684479	6.922859475
11.772512383	3.923577194	11.239548221
7.969554706	3.972727273	6.848027549
11.851936061	7.909838547	2.739003872
3.917166314	7.861994388	6.911207445
7.896185747	7.902359187	2.853452701
11.862858301	11.864045501	0.961680883
0.001543360	11.865707581	4.827565532
0.042264319	0.008904000	9.193710672
1.944277398	11.860602621	2.855783107
1.758124442	0.007598080	6.995878864
2.650067782	0.107322878	11.365390146
0.012465600	2.004705876	2.853193767
11.838995581	2.085079314	6.956779830
11.728704704	1.728919322	11.282531266
3.962873513	0.011634560	0.997154841
3.925120554	11.841844861	4.757912285
3.966435113	0.029679999	9.093762148
5.930182590	0.055086079	2.898507218
6.067660347	11.860483901	6.889456989
5.876521152	11.839351741	11.029034878
3.889741995	1.999838356	2.896176812
3.884280875	2.143370833	6.982932164
4.171583269	2.292720590	10.920282597
7.912569107	0.057579199	1.014244486
7.949491026	0.029442559	4.841547968
7.909601107	11.809434302	8.928044386
9.932233703	0.021963200	2.873649553
9.974260582	11.821899902	6.894376735
9.698236588	11.628979906	11.030588482
7.949728466	2.037591315	2.890739197
7.998403665	1.857611799	6.942020592

7.892267988	1.500027167	11.139340763
0.045113599	3.959905513	0.927242661
0.011872000	3.971421353	4.796752386
11.764201983	3.937823594	9.349330007
1.993427476	3.963110953	2.877792497
1.799439001	4.031612392	6.983450032
2.018596116	3.967266153	11.180770203
0.036565759	5.932794430	2.837139859
0.013771520	6.079888507	6.953154754
11.762539903	6.073833787	11.288227814
3.921321514	3.941622634	0.933198143
3.869678315	3.993978153	5.068115220
3.900664235	4.050251432	8.866418094
5.690012036	3.943878314	2.901614426
5.826777473	3.978425833	6.917162927
6.370396341	4.023895592	11.083669952
3.902563755	5.950483710	2.878051431
3.913723434	5.948465470	6.910430643
4.154606309	5.746641474	11.016347112
7.931801747	3.911467755	0.983949207
7.946523026	3.937823594	4.763608833
8.044467024	3.998608233	9.059323925
9.993374502	3.959430633	2.849050823
10.081227300	4.026626152	6.920788003
9.708446508	3.963229673	11.319558828
7.899866067	5.749372034	2.853711635
7.934769747	6.074664827	6.907064501
8.125908942	6.158956025	10.982685692
0.026118399	7.908770067	0.868464642
0.025168639	7.919454867	4.965836289
11.871524861	7.981545426	9.127941436
2.036404116	7.908770067	2.849309757
1.984642197	7.899035027	6.981378560
2.374281228	7.630134233	11.137787159
0.007835520	9.854353385	2.854229503
0.021488320	9.795824426	6.958333434
0.014365120	10.158276578	11.184136345
3.959668073	7.904377427	0.911447687
3.908143595	7.915062227	5.013998013
3.962279913	7.799666390	8.870302104
5.812056193	7.881583188	2.924659552
5.819772993	7.923253907	6.918716531
6.032281788	7.739950231	10.944363459
3.958480874	9.822892585	2.846979351

3.882025195	9.791787946	7.001834346
4.138579110	9.416751474	11.109822286
7.870779668	7.902359187	0.865098500
7.893099028	7.931564307	4.962729081
7.919573587	7.978458706	8.845185506
9.948973223	7.883126548	2.842318539
9.949448103	7.951034386	6.919752267
9.682565548	8.114393103	11.035767162
7.921354387	10.026259941	2.866658335
7.943673746	9.897092584	6.880394299
7.720717591	9.866225384	11.010909498
6.946188328	6.049258748	6.906805567
5.873315712	4.968194451	6.917162927
4.893519573	5.957844350	6.910430643
5.819654273	6.933366569	6.918716531
6.901193449	5.625190917	6.907064501
6.699369454	5.222017806	6.907064501
6.307356022	5.001673491	6.907064501
5.503977800	5.017225810	6.910430643
4.973299411	5.558588999	6.910430643
5.183789967	5.217506446	6.910430643
6.568777456	6.716702573	6.918716531
6.849431530	6.426313460	6.918716531
6.204188344	6.868545450	6.918716531
5.443905481	6.869613930	6.910430643
5.159333647	6.653068655	6.910430643
4.980778771	6.322077302	6.910430643

Atomic coordinates of $BaCo_{0.8}(Zr_{1/6}Ti_{1/6}Zn_{1/6}In_{1/6}Cu_{1/6}Mo_{1/6})_{0.2}O_{3-\delta}$ for oxygen vacancy formation energy calculation:

Site 1

11.8719997406	0.0000000000	0.0000000000
0.0000000000	11.8719997406	0.0000000000
0.0000000000	0.0000000000	25.8934001923

Ba	Co	In	Zr	Zn	Ti	Mo	Cu	O
27	21	1	1	1	1	1	1	80

Cartesian

1.967404053	1.962239733	1.010826557
1.963806837	1.990043957	4.899005423
1.892491735	1.984665941	8.991560897
5.939169694	1.989177301	1.063234799
5.946696542	2.020899284	4.913712874
6.075887643	2.073670323	8.920069219
9.921406439	1.977067861	0.997905750
9.898410376	1.970383925	4.908223473
9.844190953	1.958108277	8.963259411
1.991219284	5.910035807	0.951608350
1.943256406	5.925516895	4.875442429
1.858941463	5.941734046	9.021053480
5.963649758	5.914238495	1.039568231
5.970321822	5.891931007	4.870678043
5.994516957	5.899149183	8.885760464
9.844772681	5.924282207	0.963208594
9.885695464	5.968244222	4.887508753
9.884603240	5.969312702	8.838401435
1.998971700	9.934940519	0.987470710
1.951709269	9.879557640	4.847632917
1.971214965	9.918592775	9.319837425
5.960064414	9.901354632	1.048760388
5.972660605	9.901425864	4.853225891
5.920186367	9.890693576	8.963621918
9.827665129	9.905034952	0.988532339
9.869478312	9.854519593	4.845639125
9.861987081	9.895133704	8.896661585
11.861872925	0.016917600	2.855912574
11.821650590	11.723576000	6.738161852
11.470025701	11.827954622	10.948118002
3.942299338	0.032018783	2.868729807
4.191920004	0.451598998	10.887889953
7.919585459	0.022782368	2.906275238
7.938699379	11.843079549	6.851160650
7.849718740	11.720156864	10.844958696

0.051227679	3.958991369	2.835793402
0.006304032	3.894395819	6.690776929
3.906327179	3.963015977	2.796746155
3.954622474	3.973819497	6.909265440
3.781018221	4.042629608	10.807827560
8.104147567	3.914281418	2.879760396
8.003603601	4.359315201	10.892007004
11.836704285	7.928750643	6.860042087
11.414381639	7.781929622	10.883798796
3.953874538	7.902240467	2.740350329
4.063678663	7.729680951	10.942809855
8.008946001	7.953206962	6.865945782
7.801708374	7.985771858	10.818107240
3.926058442	0.036731967	6.922730008
11.772536127	3.923600938	11.239496435
7.969614066	3.972715401	6.848027549
11.851912317	7.909791059	2.738926192
3.917154442	7.861970644	6.911129765
7.896173875	7.902311699	2.853556275
11.862810813	11.864081117	0.961654990
0.001578976	11.865671965	4.827617319
0.042228703	0.008927744	9.193607099
1.944229910	11.860543261	2.855757214
1.758088826	0.007574336	6.995775290
2.650020294	0.107382238	11.365312466
0.012465600	2.004694004	2.853271447
11.838936221	2.085019954	6.956831616
11.728680960	1.728871834	11.282608946
3.962873513	0.011598944	0.997180735
3.925096810	11.841892349	4.757912285
3.966482601	0.029739359	9.093813934
5.930206334	0.055145439	2.898429537
6.067707835	11.860448285	6.889405202
5.876473664	11.839387357	11.028983091
3.889789483	1.999897716	2.896254492
3.884233387	2.143347089	6.983009844
4.171559525	2.292768078	10.920334384
7.912628467	0.057519839	1.014218592
7.949550386	0.029395071	4.841651542
7.909636723	11.809458046	8.927966706
9.932269319	0.021951328	2.873701340
9.974248710	11.821864286	6.894480309
9.698212844	11.628991778	11.030588482
7.949787826	2.037603187	2.890842771

7.998356177	1.857623671	6.942124165
7.892303604	1.500027167	11.139392550
0.045161087	3.959858025	0.927164981
0.011812640	3.971433225	4.796648812
11.764249471	3.937787978	9.349200540
1.993391860	3.963134697	2.877896071
1.799486489	4.031648008	6.983501819
2.018607988	3.967230537	11.180718416
0.036625119	5.932794430	2.837217539
0.013759648	6.079888507	6.953258327
11.762563647	6.073857531	11.288253707
3.921262154	3.941622634	0.933275823
3.869630827	3.994001897	5.068167006
3.900711723	4.050215816	8.866288627
5.690035780	3.943878314	2.901484959
5.826836833	3.978473321	6.917266501
6.370431957	4.023848104	11.083618165
3.902563755	5.950519326	2.877973751
3.913687818	5.948417982	6.910430643
4.154618181	5.746641474	11.016321218
7.931766131	3.911467755	0.983871527
7.946570514	3.937787978	4.763738300
8.044502640	3.998620105	9.059453392
9.993433862	3.959466249	2.849102610
10.081251044	4.026638024	6.920813897
9.708446508	3.963194057	11.319429361
7.899889811	5.749431394	2.853711635
7.934817235	6.074700443	6.906935034
0.026142143	7.908805683	0.868361069
0.025192383	7.919419251	4.965784502
11.871477373	7.981509810	9.128070903
2.036392244	7.908746323	2.849335651
1.984618453	7.899035027	6.981378560
2.374257484	7.620138010	11.137787159
0.007871136	9.854341513	2.854255397
0.021440832	9.795848170	6.958359327
0.014424480	10.158300322	11.184032771
3.959656201	7.904353683	0.911344113
3.908155467	7.915026611	5.014049800
3.962232425	7.799654518	8.870353891
5.812115553	7.881606932	2.924789019
5.819820481	7.923313267	6.918794212
6.032341148	7.739902743	10.944441139
3.958480874	9.822880713	2.847108818

3.882001451	9.791823562	7.001730772
4.148539717	9.416727730	11.109925860
7.870827156	7.902335443	0.865202074
7.893110900	7.931504947	4.962625507
7.919561715	7.978434962	8.845185506
9.948985095	7.883114676	2.842344433
9.949448103	7.950986898	6.919726374
9.682589292	8.114333743	11.035793055
7.921342515	10.026307429	2.866632442
7.943650002	9.897068840	6.880290725
7.720717591	9.866225384	11.010961285

Site 2

11.8719997406	0.0000000000	0.0000000000
0.0000000000	11.8719997406	0.0000000000
0.0000000000	0.0000000000	25.8934001923

Ba	Co	In	Zr	Zn	Ti	Mo	Cu	O
27	21	1	1	1	1	1	1	80

Cartesian

1.967404053	1.962239733	1.010826557
1.963806837	1.990043957	4.899005423
1.892491735	1.984665941	8.991560897
5.939169694	1.989177301	1.063234799
5.946696542	2.020899284	4.913712874
6.075887643	2.073670323	8.920069219
9.921406439	1.977067861	0.997905750
9.898410376	1.970383925	4.908223473
9.844190953	1.958108277	8.963259411
1.991219284	5.910035807	0.951608350
1.943256406	5.925516895	4.875442429
1.858941463	5.941734046	9.021053480
5.963649758	5.914238495	1.039568231
5.970321822	5.891931007	4.870678043
5.994516957	5.899149183	8.885760464
9.844772681	5.924282207	0.963208594
9.885695464	5.968244222	4.887508753
9.884603240	5.969312702	8.838401435
1.998971700	9.934940519	0.987470710
1.951709269	9.879557640	4.847632917
1.971214965	9.918592775	9.319837425
5.960064414	9.901354632	1.048760388
5.972660605	9.901425864	4.853225891
5.920186367	9.890693576	8.963621918
9.827665129	9.905034952	0.988532339

9.869478312	9.854519593	4.845639125
9.861987081	9.895133704	8.896661585
11.861872925	0.016917600	2.855912574
11.821650590	11.723576000	6.738161852
11.470025701	11.827954622	10.948118002
3.942299338	0.032018783	2.868729807
4.191920004	0.451598998	10.887889953
7.919585459	0.022782368	2.906275238
7.938699379	11.843079549	6.851160650
7.849718740	11.720156864	10.844958696
0.051227679	3.958991369	2.835793402
0.006304032	3.894395819	6.690776929
3.906327179	3.963015977	2.796746155
3.954622474	3.973819497	6.909265440
3.781018221	4.042629608	10.807827560
8.104147567	3.914281418	2.879760396
8.003603601	4.359315201	10.892007004
11.836704285	7.928750643	6.860042087
11.414381639	7.781929622	10.883798796
3.953874538	7.902240467	2.740350329
4.063678663	7.729680951	10.942809855
8.008946001	7.953206962	6.865945782
7.801708374	7.985771858	10.818107240
3.926058442	0.036731967	6.922730008
11.772536127	3.923600938	11.239496435
7.969614066	3.972715401	6.848027549
11.851912317	7.909791059	2.738926192
3.917154442	7.861970644	6.911129765
7.896173875	7.902311699	2.853556275
11.862810813	11.864081117	0.961654990
0.001578976	11.865671965	4.827617319
0.042228703	0.008927744	9.193607099
1.944229910	11.860543261	2.855757214
1.758088826	0.007574336	6.995775290
2.650020294	0.107382238	11.365312466
0.012465600	2.004694004	2.853271447
11.838936221	2.085019954	6.956831616
11.728680960	1.728871834	11.282608946
3.962873513	0.011598944	0.997180735
3.925096810	11.841892349	4.757912285
3.966482601	0.029739359	9.093813934
5.930206334	0.055145439	2.898429537
6.067707835	11.860448285	6.889405202
5.876473664	11.839387357	11.028983091

3.889789483	1.999897716	2.896254492
3.884233387	2.143347089	6.983009844
4.171559525	2.292768078	10.920334384
7.912628467	0.057519839	1.014218592
7.949550386	0.029395071	4.841651542
7.909636723	11.809458046	8.927966706
9.932269319	0.021951328	2.873701340
9.974248710	11.821864286	6.894480309
9.698212844	11.628991778	11.030588482
7.949787826	2.037603187	2.890842771
7.998356177	1.857623671	6.942124165
7.892303604	1.500027167	11.139392550
0.045161087	3.959858025	0.927164981
0.011812640	3.971433225	4.796648812
11.764249471	3.937787978	9.349200540
1.993391860	3.963134697	2.877896071
1.799486489	4.031648008	6.983501819
2.018607988	3.967230537	11.180718416
0.036625119	5.932794430	2.837217539
0.013759648	6.079888507	6.953258327
11.762563647	6.073857531	11.288253707
3.921262154	3.941622634	0.933275823
3.869630827	3.994001897	5.068167006
3.900711723	4.050215816	8.866288627
5.690035780	3.943878314	2.901484959
5.826836833	3.978473321	6.917266501
6.370431957	4.023848104	11.083618165
3.902563755	5.950519326	2.877973751
3.913687818	5.948417982	6.910430643
4.154618181	5.746641474	11.016321218
7.931766131	3.911467755	0.983871527
7.946570514	3.937787978	4.763738300
8.044502640	3.998620105	9.059453392
9.993433862	3.959466249	2.849102610
10.081251044	4.026638024	6.920813897
9.708446508	3.963194057	11.319429361
7.899889811	5.749431394	2.853711635
7.934817235	6.074700443	6.906935034
8.125920814	6.158944153	10.982711585
0.026142143	7.908805683	0.868361069
0.025192383	7.919419251	4.965784502
11.871477373	7.981509810	9.128070903
2.036392244	7.908746323	2.849335651
1.984618453	7.899035027	6.981378560

2.374257484	7.620138010	11.137787159
0.007871136	9.854341513	2.854255397
0.021440832	9.795848170	6.958359327
0.014424480	10.158300322	11.184032771
3.959656201	7.904353683	0.911344113
3.908155467	7.915026611	5.014049800
3.962232425	7.799654518	8.870353891
5.812115553	7.881606932	2.924789019
5.819820481	7.923313267	6.918794212
6.032341148	7.739902743	10.944441139
3.958480874	9.822880713	2.847108818
3.882001451	9.791823562	7.001730772
4.148539717	9.416727730	11.109925860
7.870827156	7.902335443	0.865202074
7.893110900	7.931504947	4.962625507
7.919561715	7.978434962	8.845185506
9.948985095	7.883114676	2.842344433
9.949448103	7.950986898	6.919726374
9.682589292	8.114333743	11.035793055
7.921342515	10.026307429	2.866632442
7.943650002	9.897068840	6.880290725

Site 3

11.8719997406	0.0000000000	0.0000000000
0.0000000000	11.8719997406	0.0000000000
0.0000000000	0.0000000000	25.8934001923

Ba	Co	In	Zr	Zn	Ti	Mo	Cu	O
27	21	1	1	1	1	1	1	80

Cartesian

1.967404053	1.962239733	1.010826557
1.963806837	1.990043957	4.899005423
1.892491735	1.984665941	8.991560897
5.939169694	1.989177301	1.063234799
5.946696542	2.020899284	4.913712874
6.075887643	2.073670323	8.920069219
9.921406439	1.977067861	0.997905750
9.898410376	1.970383925	4.908223473
9.844190953	1.958108277	8.963259411
1.991219284	5.910035807	0.951608350
1.943256406	5.925516895	4.875442429
1.858941463	5.941734046	9.021053480
5.963649758	5.914238495	1.039568231
5.970321822	5.891931007	4.870678043
5.994516957	5.899149183	8.885760464

9.844772681	5.924282207	0.963208594
9.885695464	5.968244222	4.887508753
9.884603240	5.969312702	8.838401435
1.998971700	9.934940519	0.987470710
1.951709269	9.879557640	4.847632917
1.971214965	9.918592775	9.319837425
5.960064414	9.901354632	1.048760388
5.972660605	9.901425864	4.853225891
5.920186367	9.890693576	8.963621918
9.827665129	9.905034952	0.988532339
9.869478312	9.854519593	4.845639125
9.861987081	9.895133704	8.896661585
11.861872925	0.016917600	2.855912574
11.821650590	11.723576000	6.738161852
11.470025701	11.827954622	10.948118002
3.942299338	0.032018783	2.868729807
4.191920004	0.451598998	10.887889953
7.919585459	0.022782368	2.906275238
7.938699379	11.843079549	6.851160650
7.849718740	11.720156864	10.844958696
0.051227679	3.958991369	2.835793402
0.006304032	3.894395819	6.690776929
3.906327179	3.963015977	2.796746155
3.954622474	3.973819497	6.909265440
3.781018221	4.042629608	10.807827560
8.104147567	3.914281418	2.879760396
8.003603601	4.359315201	10.892007004
11.836704285	7.928750643	6.860042087
11.414381639	7.781929622	10.883798796
3.953874538	7.902240467	2.740350329
4.063678663	7.729680951	10.942809855
8.008946001	7.953206962	6.865945782
7.801708374	7.985771858	10.818107240
3.926058442	0.036731967	6.922730008
11.772536127	3.923600938	11.239496435
7.969614066	3.972715401	6.848027549
11.851912317	7.909791059	2.738926192
3.917154442	7.861970644	6.911129765
7.896173875	7.902311699	2.853556275
11.862810813	11.864081117	0.961654990
0.001578976	11.865671965	4.827617319
0.042228703	0.008927744	9.193607099
1.944229910	11.860543261	2.855757214
1.758088826	0.007574336	6.995775290

2.650020294	0.107382238	11.365312466
0.012465600	2.004694004	2.853271447
11.838936221	2.085019954	6.956831616
11.728680960	1.728871834	11.282608946
3.962873513	0.011598944	0.997180735
3.925096810	11.841892349	4.757912285
3.966482601	0.029739359	9.093813934
5.930206334	0.055145439	2.898429537
6.067707835	11.860448285	6.889405202
5.876473664	11.839387357	11.028983091
3.889789483	1.999897716	2.896254492
3.884233387	2.143347089	6.983009844
4.171559525	2.292768078	10.920334384
7.912628467	0.057519839	1.014218592
7.949550386	0.029395071	4.841651542
7.909636723	11.809458046	8.927966706
9.932269319	0.021951328	2.873701340
9.974248710	11.821864286	6.894480309
9.698212844	11.628991778	11.030588482
7.949787826	2.037603187	2.890842771
7.998356177	1.857623671	6.942124165
7.892303604	1.500027167	11.139392550
0.045161087	3.959858025	0.927164981
0.011812640	3.971433225	4.796648812
11.764249471	3.937787978	9.349200540
1.993391860	3.963134697	2.877896071
1.799486489	4.031648008	6.983501819
2.018607988	3.967230537	11.180718416
0.036625119	5.932794430	2.837217539
0.013759648	6.079888507	6.953258327
11.762563647	6.073857531	11.288253707
3.921262154	3.941622634	0.933275823
3.869630827	3.994001897	5.068167006
3.900711723	4.050215816	8.866288627
5.690035780	3.943878314	2.901484959
5.826836833	3.978473321	6.917266501
6.370431957	4.023848104	11.083618165
3.902563755	5.950519326	2.877973751
3.913687818	5.948417982	6.910430643
4.154618181	5.746641474	11.016321218
7.931766131	3.911467755	0.983871527
7.946570514	3.937787978	4.763738300
8.044502640	3.998620105	9.059453392
9.993433862	3.959466249	2.849102610

10.081251044	4.026638024	6.920813897
9.708446508	3.963194057	11.319429361
7.899889811	5.749431394	2.853711635
7.934817235	6.074700443	6.906935034
8.125920814	6.158944153	10.982711585
0.026142143	7.908805683	0.868361069
0.025192383	7.919419251	4.965784502
11.871477373	7.981509810	9.128070903
2.036392244	7.908746323	2.849335651
1.984618453	7.899035027	6.981378560
2.374257484	7.620138010	11.137787159
0.007871136	9.854341513	2.854255397
0.021440832	9.795848170	6.958359327
0.014424480	10.158300322	11.184032771
3.959656201	7.904353683	0.911344113
3.908155467	7.915026611	5.014049800
3.962232425	7.799654518	8.870353891
5.812115553	7.881606932	2.924789019
5.819820481	7.923313267	6.918794212
6.032341148	7.739902743	10.944441139
3.958480874	9.822880713	2.847108818
3.882001451	9.791823562	7.001730772
4.148539717	9.416727730	11.109925860
7.870827156	7.902335443	0.865202074
7.893110900	7.931504947	4.962625507
7.919561715	7.978434962	8.845185506
9.948985095	7.883114676	2.842344433
9.949448103	7.950986898	6.919726374
9.682589292	8.114333743	11.035793055
7.921342515	10.026307429	2.866632442
7.943650002	9.897068840	6.880290725

Site 4

11.8719997406	0.0000000000	0.0000000000
0.0000000000	11.8719997406	0.0000000000
0.0000000000	0.0000000000	25.8934001923

Ba Co In Zr Zn Ti Mo Cu O
27 21 1 1 1 1 1 1 80

Cartesian

1.967404053	1.962239733	1.010826557
1.963806837	1.990043957	4.899005423
1.892491735	1.984665941	8.991560897
5.939169694	1.989177301	1.063234799
5.946696542	2.020899284	4.913712874

6.075887643	2.073670323	8.920069219
9.921406439	1.977067861	0.997905750
9.898410376	1.970383925	4.908223473
9.844190953	1.958108277	8.963259411
1.991219284	5.910035807	0.951608350
1.943256406	5.925516895	4.875442429
1.858941463	5.941734046	9.021053480
5.963649758	5.914238495	1.039568231
5.970321822	5.891931007	4.870678043
5.994516957	5.899149183	8.885760464
9.844772681	5.924282207	0.963208594
9.885695464	5.968244222	4.887508753
9.884603240	5.969312702	8.838401435
1.998971700	9.934940519	0.987470710
1.951709269	9.879557640	4.847632917
1.971214965	9.918592775	9.319837425
5.960064414	9.901354632	1.048760388
5.972660605	9.901425864	4.853225891
5.920186367	9.890693576	8.963621918
9.827665129	9.905034952	0.988532339
9.869478312	9.854519593	4.845639125
9.861987081	9.895133704	8.896661585
11.861872925	0.016917600	2.855912574
11.821650590	11.723576000	6.738161852
11.470025701	11.827954622	10.948118002
3.942299338	0.032018783	2.868729807
4.191920004	0.451598998	10.887889953
7.919585459	0.022782368	2.906275238
7.938699379	11.843079549	6.851160650
7.849718740	11.720156864	10.844958696
0.051227679	3.958991369	2.835793402
0.006304032	3.894395819	6.690776929
3.906327179	3.963015977	2.796746155
3.954622474	3.973819497	6.909265440
3.781018221	4.042629608	10.807827560
8.104147567	3.914281418	2.879760396
8.003603601	4.359315201	10.892007004
11.836704285	7.928750643	6.860042087
11.414381639	7.781929622	10.883798796
3.953874538	7.902240467	2.740350329
4.063678663	7.729680951	10.942809855
8.008946001	7.953206962	6.865945782
7.801708374	7.985771858	10.818107240
3.926058442	0.036731967	6.922730008

11.772536127	3.923600938	11.239496435
7.969614066	3.972715401	6.848027549
11.851912317	7.909791059	2.738926192
3.917154442	7.861970644	6.911129765
7.896173875	7.902311699	2.853556275
11.862810813	11.864081117	0.961654990
0.001578976	11.865671965	4.827617319
0.042228703	0.008927744	9.193607099
1.944229910	11.860543261	2.855757214
1.758088826	0.007574336	6.995775290
2.650020294	0.107382238	11.365312466
0.012465600	2.004694004	2.853271447
11.838936221	2.085019954	6.956831616
11.728680960	1.728871834	11.282608946
3.962873513	0.011598944	0.997180735
3.925096810	11.841892349	4.757912285
3.966482601	0.029739359	9.093813934
5.930206334	0.055145439	2.898429537
6.067707835	11.860448285	6.889405202
5.876473664	11.839387357	11.028983091
3.889789483	1.999897716	2.896254492
3.884233387	2.143347089	6.983009844
4.171559525	2.292768078	10.920334384
7.912628467	0.057519839	1.014218592
7.949550386	0.029395071	4.841651542
7.909636723	11.809458046	8.927966706
9.932269319	0.021951328	2.873701340
9.974248710	11.821864286	6.894480309
9.698212844	11.628991778	11.030588482
7.949787826	2.037603187	2.890842771
7.998356177	1.857623671	6.942124165
7.892303604	1.500027167	11.139392550
0.045161087	3.959858025	0.927164981
0.011812640	3.971433225	4.796648812
11.764249471	3.937787978	9.349200540
1.993391860	3.963134697	2.877896071
1.799486489	4.031648008	6.983501819
2.018607988	3.967230537	11.180718416
0.036625119	5.932794430	2.837217539
0.013759648	6.079888507	6.953258327
11.762563647	6.073857531	11.288253707
3.921262154	3.941622634	0.933275823
3.869630827	3.994001897	5.068167006
3.900711723	4.050215816	8.866288627

5.690035780	3.943878314	2.901484959
5.826836833	3.978473321	6.917266501
6.370431957	4.023848104	11.083618165
3.902563755	5.950519326	2.877973751
3.913687818	5.948417982	6.910430643
4.154618181	5.746641474	11.016321218
7.931766131	3.911467755	0.983871527
7.946570514	3.937787978	4.763738300
8.044502640	3.998620105	9.059453392
9.993433862	3.959466249	2.849102610
10.081251044	4.026638024	6.920813897
9.708446508	3.963194057	11.319429361
7.899889811	5.749431394	2.853711635
7.934817235	6.074700443	6.906935034
8.125920814	6.158944153	10.982711585
0.026142143	7.908805683	0.868361069
0.025192383	7.919419251	4.965784502
11.871477373	7.981509810	9.128070903
2.036392244	7.908746323	2.849335651
1.984618453	7.899035027	6.981378560
2.374257484	7.620138010	11.137787159
0.007871136	9.854341513	2.854255397
0.021440832	9.795848170	6.958359327
0.014424480	10.158300322	11.184032771
3.959656201	7.904353683	0.911344113
3.908155467	7.915026611	5.014049800
3.962232425	7.799654518	8.870353891
5.812115553	7.881606932	2.924789019
5.819820481	7.923313267	6.918794212
6.032341148	7.739902743	10.944441139
3.958480874	9.822880713	2.847108818
3.882001451	9.791823562	7.001730772
4.148539717	9.416727730	11.109925860
7.870827156	7.902335443	0.865202074
7.893110900	7.931504947	4.962625507
7.919561715	7.978434962	8.845185506
9.948985095	7.883114676	2.842344433
9.949448103	7.950986898	6.919726374
7.921342515	10.026307429	2.866632442
7.943650002	9.897068840	6.880290725
7.720717591	9.866225384	11.010961285

Site 5

11.8719997406	0.0000000000	0.0000000000

0.0000000000	11.8719997406	0.0000000000
0.0000000000	0.0000000000	25.8934001923

Ba Co In Zr Zn Ti Mo Cu O
27 21 1 1 1 1 1 1 80
Cartesian

1.967404053	1.962239733	1.010826557
1.963806837	1.990043957	4.899005423
1.892491735	1.984665941	8.991560897
5.939169694	1.989177301	1.063234799
5.946696542	2.020899284	4.913712874
6.075887643	2.073670323	8.920069219
9.921406439	1.977067861	0.997905750
9.898410376	1.970383925	4.908223473
9.844190953	1.958108277	8.963259411
1.991219284	5.910035807	0.951608350
1.943256406	5.925516895	4.875442429
1.858941463	5.941734046	9.021053480
5.963649758	5.914238495	1.039568231
5.970321822	5.891931007	4.870678043
5.994516957	5.899149183	8.885760464
9.844772681	5.924282207	0.963208594
9.885695464	5.968244222	4.887508753
9.884603240	5.969312702	8.838401435
1.998971700	9.934940519	0.987470710
1.951709269	9.879557640	4.847632917
1.971214965	9.918592775	9.319837425
5.960064414	9.901354632	1.048760388
5.972660605	9.901425864	4.853225891
5.920186367	9.890693576	8.963621918
9.827665129	9.905034952	0.988532339
9.869478312	9.854519593	4.845639125
9.861987081	9.895133704	8.896661585
11.861872925	0.016917600	2.855912574
11.821650590	11.723576000	6.738161852
11.470025701	11.827954622	10.948118002
3.942299338	0.032018783	2.868729807
4.191920004	0.451598998	10.887889953
7.919585459	0.022782368	2.906275238
7.938699379	11.843079549	6.851160650
7.849718740	11.720156864	10.844958696
0.051227679	3.958991369	2.835793402
0.006304032	3.894395819	6.690776929
3.906327179	3.963015977	2.796746155
3.954622474	3.973819497	6.909265440

3.781018221	4.042629608	10.807827560
8.104147567	3.914281418	2.879760396
8.003603601	4.359315201	10.892007004
11.836704285	7.928750643	6.860042087
11.414381639	7.781929622	10.883798796
3.953874538	7.902240467	2.740350329
4.063678663	7.729680951	10.942809855
8.008946001	7.953206962	6.865945782
7.801708374	7.985771858	10.818107240
3.926058442	0.036731967	6.922730008
11.772536127	3.923600938	11.239496435
7.969614066	3.972715401	6.848027549
11.851912317	7.909791059	2.738926192
3.917154442	7.861970644	6.911129765
7.896173875	7.902311699	2.853556275
11.862810813	11.864081117	0.961654990
0.001578976	11.865671965	4.827617319
0.042228703	0.008927744	9.193607099
1.944229910	11.860543261	2.855757214
1.758088826	0.007574336	6.995775290
2.650020294	0.107382238	11.365312466
0.012465600	2.004694004	2.853271447
11.838936221	2.085019954	6.956831616
11.728680960	1.728871834	11.282608946
3.962873513	0.011598944	0.997180735
3.925096810	11.841892349	4.757912285
3.966482601	0.029739359	9.093813934
5.930206334	0.055145439	2.898429537
6.067707835	11.860448285	6.889405202
5.876473664	11.839387357	11.028983091
3.889789483	1.999897716	2.896254492
3.884233387	2.143347089	6.983009844
4.171559525	2.292768078	10.920334384
7.912628467	0.057519839	1.014218592
7.949550386	0.029395071	4.841651542
7.909636723	11.809458046	8.927966706
9.932269319	0.021951328	2.873701340
9.974248710	11.821864286	6.894480309
9.698212844	11.628991778	11.030588482
7.949787826	2.037603187	2.890842771
7.998356177	1.857623671	6.942124165
7.892303604	1.500027167	11.139392550
0.045161087	3.959858025	0.927164981
0.011812640	3.971433225	4.796648812

11.764249471	3.937787978	9.349200540
1.993391860	3.963134697	2.877896071
1.799486489	4.031648008	6.983501819
2.018607988	3.967230537	11.180718416
0.036625119	5.932794430	2.837217539
0.013759648	6.079888507	6.953258327
11.762563647	6.073857531	11.288253707
3.921262154	3.941622634	0.933275823
3.869630827	3.994001897	5.068167006
3.900711723	4.050215816	8.866288627
5.690035780	3.943878314	2.901484959
5.826836833	3.978473321	6.917266501
6.370431957	4.023848104	11.083618165
3.902563755	5.950519326	2.877973751
3.913687818	5.948417982	6.910430643
4.154618181	5.746641474	11.016321218
7.931766131	3.911467755	0.983871527
7.946570514	3.937787978	4.763738300
8.044502640	3.998620105	9.059453392
9.993433862	3.959466249	2.849102610
10.081251044	4.026638024	6.920813897
9.708446508	3.963194057	11.319429361
7.899889811	5.749431394	2.853711635
7.934817235	6.074700443	6.906935034
8.125920814	6.158944153	10.982711585
0.026142143	7.908805683	0.868361069
0.025192383	7.919419251	4.965784502
11.871477373	7.981509810	9.128070903
2.036392244	7.908746323	2.849335651
1.984618453	7.899035027	6.981378560
2.374257484	7.620138010	11.137787159
0.007871136	9.854341513	2.854255397
0.021440832	9.795848170	6.958359327
0.014424480	10.158300322	11.184032771
3.959656201	7.904353683	0.911344113
3.908155467	7.915026611	5.014049800
3.962232425	7.799654518	8.870353891
5.812115553	7.881606932	2.924789019
5.819820481	7.923313267	6.918794212
6.032341148	7.739902743	10.944441139
3.958480874	9.822880713	2.847108818
3.882001451	9.791823562	7.001730772
4.148539717	9.416727730	11.109925860
7.870827156	7.902335443	0.865202074

7.893110900	7.931504947	4.962625507
9.948985095	7.883114676	2.842344433
9.949448103	7.950986898	6.919726374
9.682589292	8.114333743	11.035793055
7.921342515	10.026307429	2.866632442
7.943650002	9.897068840	6.880290725
7.720717591	9.866225384	11.010961285

Atomic coordinates of $Ba_{0.5}Sr_{0.5}Co_{0.8}Fe_{0.2}O_{3-\delta}$ for oxygen vacancy formation energy calculation:
Site 1:

11.8719997406	0.0000000000	0.0000000000
0.0000000000	11.8719997406	0.0000000000
0.0000000000	0.0000000000	25.8934001923

Sr	Ba	Co	Fe	O
13	14	22	5	80

Cartesian

1.991148052	1.965373941	1.510025419
1.961847957	2.013871060	5.068296473
5.929541502	1.954238005	4.988467121
5.961026046	1.897549207	8.947568010
5.956324734	5.991750781	8.662507567
9.929218215	5.961762110	1.460543131
9.858342377	5.991620189	4.957990589
9.816529194	5.999538813	8.553496353
1.931099478	9.886918280	4.969720299
5.919545279	9.923828327	1.448166086
9.903657800	9.874476424	1.426001335
9.863328616	9.860681161	5.016639140
9.892961128	9.804206058	8.691844790
1.928262070	2.066760819	8.982213379
5.893011359	1.925923286	1.175612156
9.948830759	1.970775701	1.208030693
9.894979368	1.986874133	4.989994831
9.984363654	1.953418837	8.941949142
1.919987286	5.891527359	1.209739657
1.973185717	5.898531839	4.992247557
1.976592981	5.987476861	8.914424458
5.942007102	5.933043742	1.185270394
5.936189822	5.914891455	4.956618238
1.959865333	9.925941543	1.215695139
1.957847093	9.802389642	8.731150971
5.958711006	9.926095879	5.013531932
5.893794911	9.924398183	8.747101306
11.854987165	11.823217694	3.091905024

11.843388221	11.826256926	6.837048747
11.838033949	11.362738440	10.651716250
3.906612107	11.870408893	3.100708780
3.886584043	0.052296159	6.823610072
3.545952627	11.706634656	10.655056499
7.965185810	0.021559552	6.860534061
7.999104113	11.384369223	10.673751534
11.867678333	3.922318762	3.090221953
11.744328255	3.976051433	6.845723036
11.406439271	4.361499649	10.696175218
3.967942857	3.923589066	2.998714676
7.918113331	3.893137387	3.032971645
7.865603476	3.893315467	6.846240904
7.871860020	4.135029382	10.613135084
11.852031037	7.930234643	3.042319162
11.463614822	7.834629429	10.628671124
3.876231659	7.927812755	3.043846873
3.956901898	7.969815890	6.807374911
4.114918214	8.115271631	10.676185513
7.904021267	7.924037459	6.824516341
7.799915702	7.759135382	10.596718668
7.926174419	0.000237440	3.011272975
3.964903625	3.931531434	6.826484240
4.217848452	4.312598882	10.603088445
11.845038429	7.892564788	6.800435479
7.922185427	7.923230163	2.990843083
0.004926880	11.864627229	1.228926667
11.838781885	11.857325949	4.964852340
11.862240957	11.809659870	8.791042406
1.964436053	0.020051808	3.141879286
1.922005526	0.012952352	6.825552077
1.740767578	11.727208832	10.876678111
0.031567647	1.980866901	3.087736186
0.022307488	2.084236402	6.970710479
11.730010624	2.783699011	11.132142397
3.924396362	11.862573373	1.226751621
3.974804873	0.040578495	4.963557670
3.963407753	0.000997248	8.766029381
5.994350749	0.001151584	3.030019797
6.070367163	0.084018142	6.937489247
6.423274228	11.836775517	11.009666615
3.963621449	1.952860853	3.093510414
3.993158985	1.978136341	6.860197447
3.977701641	1.409206369	10.976523062

7.925568947	11.756105279	1.145990106
7.938117651	0.045552863	4.901750123
8.113205903	11.866740445	8.936563315
9.867008936	11.824428638	3.036855655
9.898137320	11.844884093	6.910378857
10.133024835	11.633313186	10.893819542
7.919205555	1.929271190	2.965985418
7.934662899	1.966489909	7.040182472
8.188201325	2.490876138	10.831830742
11.864247325	3.954444394	1.227424849
0.017879232	3.937942314	4.969901553
11.809458046	4.156731397	8.848033780
1.970051509	3.908001131	3.063758898
2.093116658	3.912987371	6.941062536
2.734750756	3.766546254	11.059174796
11.826589342	5.997579933	3.087321892
11.770494143	5.975118109	6.815557225
0.016430848	6.102077275	10.896745496
3.887984939	3.868372395	1.119475264
3.967978473	3.918828394	4.929223021
4.130969158	3.828292524	8.881410373
5.929624606	3.911123467	2.977922276
5.934373406	3.859646476	6.845930183
5.823477057	3.696762639	10.840505031
3.947902922	5.928045630	2.950967246
3.995818313	5.784952418	6.996034224
4.128523526	5.997413725	10.612280602
7.972629554	4.004710440	1.190966942
7.872251796	3.891558411	4.912159270
7.753923575	4.144099589	8.729157179
9.891500872	4.035743848	3.089781765
9.841044873	4.007334152	6.914910202
9.666538349	4.614586939	10.726341030
7.969602194	6.038657052	3.043458472
7.954227954	5.985185565	6.805898987
11.759678751	7.931196275	1.173307643
11.848564413	7.963084466	4.954236046
11.781665695	7.847284981	8.751684438
1.932630966	7.968486226	3.024996477
1.944265526	7.886035188	6.868379761
2.460911210	7.946523026	10.947574241
11.844397341	9.877159496	3.095348846
11.818528254	9.827914441	6.845748930
0.045647839	9.537952720	10.831675382

4.001303177	7.987564529	1.198501921
3.913782794	7.869901140	4.924717569
4.039447912	7.914967251	8.751114783
6.052191132	7.939067411	3.013577488
5.897130943	7.883007828	6.886064954
5.910534431	8.079477551	10.856895553
3.937586154	9.902518088	3.087943333
3.898159243	9.900511720	6.808229393
3.933169770	10.011847333	10.792006693
7.989891441	7.965838770	1.141588228
7.959249810	7.963998610	4.980103552
7.886331988	7.875575956	8.685293759
9.870321224	7.943293842	3.117151089
9.905058696	7.957718322	6.869415497
9.710286668	7.737611447	10.728645542
7.955806930	9.868599784	3.059072192
8.000528753	9.936602599	6.896059806
8.053513488	9.668829645	10.804383738

Site 2:

11.8719997406	0.0000000000	0.0000000000
0.0000000000	11.8719997406	0.0000000000
0.0000000000	0.0000000000	25.8934001923

Sr	Ba	Co	Fe	O
13	14	22	5	80

Cartesian

1.991148052	1.965373941	1.510025419
1.961847957	2.013871060	5.068296473
5.929541502	1.954238005	4.988467121
5.961026046	1.897549207	8.947568010
5.956324734	5.991750781	8.662507567
9.929218215	5.961762110	1.460543131
9.858342377	5.991620189	4.957990589
9.816529194	5.999538813	8.553496353
1.931099478	9.886918280	4.969720299
5.919545279	9.923828327	1.448166086
9.903657800	9.874476424	1.426001335
9.863328616	9.860681161	5.016639140
9.892961128	9.804206058	8.691844790
1.928262070	2.066760819	8.982213379
5.893011359	1.925923286	1.175612156
9.948830759	1.970775701	1.208030693
9.894979368	1.986874133	4.989994831
9.984363654	1.953418837	8.941949142

1.919987286	5.891527359	1.209739657
1.973185717	5.898531839	4.992247557
1.976592981	5.987476861	8.914424458
5.942007102	5.933043742	1.185270394
5.936189822	5.914891455	4.956618238
1.959865333	9.925941543	1.215695139
1.957847093	9.802389642	8.731150971
5.958711006	9.926095879	5.013531932
5.893794911	9.924398183	8.747101306
11.854987165	11.823217694	3.091905024
11.843388221	11.826256926	6.837048747
11.838033949	11.362738440	10.651716250
3.906612107	11.870408893	3.100708780
3.886584043	0.052296159	6.823610072
3.545952627	11.706634656	10.655056499
7.965185810	0.021559552	6.860534061
7.999104113	11.384369223	10.673751534
11.867678333	3.922318762	3.090221953
11.744328255	3.976051433	6.845723036
11.406439271	4.361499649	10.696175218
3.967942857	3.923589066	2.998714676
7.918113331	3.893137387	3.032971645
7.865603476	3.893315467	6.846240904
7.871860020	4.135029382	10.613135084
11.852031037	7.930234643	3.042319162
11.463614822	7.834629429	10.628671124
3.876231659	7.927812755	3.043846873
3.956901898	7.969815890	6.807374911
4.114918214	8.115271631	10.676185513
7.904021267	7.924037459	6.824516341
7.799915702	7.759135382	10.596718668
7.926174419	0.000237440	3.011272975
3.964903625	3.931531434	6.826484240
4.217848452	4.312598882	10.603088445
11.845038429	7.892564788	6.800435479
7.922185427	7.923230163	2.990843083
0.004926880	11.864627229	1.228926667
11.838781885	11.857325949	4.964852340
11.862240957	11.809659870	8.791042406
1.964436053	0.020051808	3.141879286
1.922005526	0.012952352	6.825552077
1.740767578	11.727208832	10.876678111
0.031567647	1.980866901	3.087736186
0.022307488	2.084236402	6.970710479

11.730010624	2.783699011	11.132142397
3.924396362	11.862573373	1.226751621
3.974804873	0.040578495	4.963557670
3.963407753	0.000997248	8.766029381
5.994350749	0.001151584	3.030019797
6.070367163	0.084018142	6.937489247
6.423274228	11.836775517	11.009666615
3.963621449	1.952860853	3.093510414
3.993158985	1.978136341	6.860197447
3.977701641	1.409206369	10.976523062
7.925568947	11.756105279	1.145990106
7.938117651	0.045552863	4.901750123
8.113205903	11.866740445	8.936563315
9.867008936	11.824428638	3.036855655
9.898137320	11.844884093	6.910378857
10.133024835	11.633313186	10.893819542
7.919205555	1.929271190	2.965985418
7.934662899	1.966489909	7.040182472
8.188201325	2.490876138	10.831830742
11.864247325	3.954444394	1.227424849
0.017879232	3.937942314	4.969901553
11.809458046	4.156731397	8.848033780
1.970051509	3.908001131	3.063758898
2.093116658	3.912987371	6.941062536
2.734750756	3.766546254	11.059174796
11.826589342	5.997579933	3.087321892
11.770494143	5.975118109	6.815557225
0.016430848	6.102077275	10.896745496
3.887984939	3.868372395	1.119475264
3.967978473	3.918828394	4.929223021
4.130969158	3.828292524	8.881410373
5.929624606	3.911123467	2.977922276
5.934373406	3.859646476	6.845930183
5.823477057	3.696762639	10.840505031
3.947902922	5.928045630	2.950967246
3.995818313	5.784952418	6.996034224
4.128523526	5.997413725	10.612280602
7.972629554	4.004710440	1.190966942
7.872251796	3.891558411	4.912159270
7.753923575	4.144099589	8.729157179
9.891500872	4.035743848	3.089781765
9.841044873	4.007334152	6.914910202
9.666538349	4.614586939	10.726341030
7.969602194	6.038657052	3.043458472

7.954227954	5.985185565	6.805898987
7.550995483	5.920661247	10.760882826
11.759678751	7.931196275	1.173307643
11.848564413	7.963084466	4.954236046
11.781665695	7.847284981	8.751684438
1.932630966	7.968486226	3.024996477
1.944265526	7.886035188	6.868379761
2.460911210	7.946523026	10.947574241
11.844397341	9.877159496	3.095348846
11.818528254	9.827914441	6.845748930
0.045647839	9.537952720	10.831675382
4.001303177	7.987564529	1.198501921
3.913782794	7.869901140	4.924717569
4.039447912	7.914967251	8.751114783
6.052191132	7.939067411	3.013577488
5.897130943	7.883007828	6.886064954
3.937586154	9.902518088	3.087943333
3.898159243	9.900511720	6.808229393
3.933169770	10.011847333	10.792006693
7.989891441	7.965838770	1.141588228
7.959249810	7.963998610	4.980103552
7.886331988	7.875575956	8.685293759
9.870321224	7.943293842	3.117151089
9.905058696	7.957718322	6.869415497
9.710286668	7.737611447	10.728645542
7.955806930	9.868599784	3.059072192
8.000528753	9.936602599	6.896059806
8.053513488	9.668829645	10.804383738

Site 3:

11.8719997406	0.0000000000	0.0000000000
0.0000000000	11.8719997406	0.0000000000
0.0000000000	0.0000000000	25.8934001923

Sr	Ba	Co	Fe	O
13	14	22	5	80

Cartesian

1.991148052	1.965373941	1.510025419
1.961847957	2.013871060	5.068296473
5.929541502	1.954238005	4.988467121
5.961026046	1.897549207	8.947568010
5.956324734	5.991750781	8.662507567
9.929218215	5.961762110	1.460543131
9.858342377	5.991620189	4.957990589
9.816529194	5.999538813	8.553496353

1.931099478	9.886918280	4.969720299
5.919545279	9.923828327	1.448166086
9.903657800	9.874476424	1.426001335
9.863328616	9.860681161	5.016639140
9.892961128	9.804206058	8.691844790
1.928262070	2.066760819	8.982213379
5.893011359	1.925923286	1.175612156
9.948830759	1.970775701	1.208030693
9.894979368	1.986874133	4.989994831
9.984363654	1.953418837	8.941949142
1.919987286	5.891527359	1.209739657
1.973185717	5.898531839	4.992247557
1.976592981	5.987476861	8.914424458
5.942007102	5.933043742	1.185270394
5.936189822	5.914891455	4.956618238
1.959865333	9.925941543	1.215695139
1.957847093	9.802389642	8.731150971
5.958711006	9.926095879	5.013531932
5.893794911	9.924398183	8.747101306
11.854987165	11.823217694	3.091905024
11.843388221	11.826256926	6.837048747
11.838033949	11.362738440	10.651716250
3.906612107	11.870408893	3.100708780
3.886584043	0.052296159	6.823610072
3.545952627	11.706634656	10.655056499
7.965185810	0.021559552	6.860534061
7.999104113	11.384369223	10.673751534
11.867678333	3.922318762	3.090221953
11.744328255	3.976051433	6.845723036
11.406439271	4.361499649	10.696175218
3.967942857	3.923589066	2.998714676
7.918113331	3.893137387	3.032971645
7.865603476	3.893315467	6.846240904
7.871860020	4.135029382	10.613135084
11.852031037	7.930234643	3.042319162
11.463614822	7.834629429	10.628671124
3.876231659	7.927812755	3.043846873
3.956901898	7.969815890	6.807374911
4.114918214	8.115271631	10.676185513
7.904021267	7.924037459	6.824516341
7.799915702	7.759135382	10.596718668
7.926174419	0.000237440	3.011272975
3.964903625	3.931531434	6.826484240
4.217848452	4.312598882	10.603088445

11.845038429	7.892564788	6.800435479
7.922185427	7.923230163	2.990843083
0.004926880	11.864627229	1.228926667
11.838781885	11.857325949	4.964852340
11.862240957	11.809659870	8.791042406
1.964436053	0.020051808	3.141879286
1.922005526	0.012952352	6.825552077
1.740767578	11.727208832	10.876678111
0.031567647	1.980866901	3.087736186
0.022307488	2.084236402	6.970710479
11.730010624	2.783699011	11.132142397
3.924396362	11.862573373	1.226751621
3.974804873	0.040578495	4.963557670
3.963407753	0.000997248	8.766029381
5.994350749	0.001151584	3.030019797
6.070367163	0.084018142	6.937489247
6.423274228	11.836775517	11.009666615
3.963621449	1.952860853	3.093510414
3.993158985	1.978136341	6.860197447
3.977701641	1.409206369	10.976523062
7.925568947	11.756105279	1.145990106
7.938117651	0.045552863	4.901750123
8.113205903	11.866740445	8.936563315
9.867008936	11.824428638	3.036855655
9.898137320	11.844884093	6.910378857
10.133024835	11.633313186	10.893819542
7.919205555	1.929271190	2.965985418
7.934662899	1.966489909	7.040182472
8.188201325	2.490876138	10.831830742
11.864247325	3.954444394	1.227424849
0.017879232	3.937942314	4.969901553
11.809458046	4.156731397	8.848033780
1.970051509	3.908001131	3.063758898
2.093116658	3.912987371	6.941062536
2.734750756	3.766546254	11.059174796
11.826589342	5.997579933	3.087321892
11.770494143	5.975118109	6.815557225
0.016430848	6.102077275	10.896745496
3.887984939	3.868372395	1.119475264
3.967978473	3.918828394	4.929223021
4.130969158	3.828292524	8.881410373
5.929624606	3.911123467	2.977922276
5.934373406	3.859646476	6.845930183
5.823477057	3.696762639	10.840505031

3.947902922	5.928045630	2.950967246
3.995818313	5.784952418	6.996034224
4.128523526	5.997413725	10.612280602
7.972629554	4.004710440	1.190966942
7.872251796	3.891558411	4.912159270
7.753923575	4.144099589	8.729157179
9.891500872	4.035743848	3.089781765
9.841044873	4.007334152	6.914910202
9.666538349	4.614586939	10.726341030
7.969602194	6.038657052	3.043458472
7.954227954	5.985185565	6.805898987
7.550995483	5.920661247	10.760882826
11.759678751	7.931196275	1.173307643
11.848564413	7.963084466	4.954236046
11.781665695	7.847284981	8.751684438
1.932630966	7.968486226	3.024996477
1.944265526	7.886035188	6.868379761
2.460911210	7.946523026	10.947574241
11.844397341	9.877159496	3.095348846
11.818528254	9.827914441	6.845748930
0.045647839	9.537952720	10.831675382
4.001303177	7.987564529	1.198501921
3.913782794	7.869901140	4.924717569
4.039447912	7.914967251	8.751114783
6.052191132	7.939067411	3.013577488
5.897130943	7.883007828	6.886064954
5.910534431	8.079477551	10.856895553
3.937586154	9.902518088	3.087943333
3.898159243	9.900511720	6.808229393
3.933169770	10.011847333	10.792006693
7.989891441	7.965838770	1.141588228
7.959249810	7.963998610	4.980103552
7.886331988	7.875575956	8.685293759
9.870321224	7.943293842	3.117151089
9.905058696	7.957718322	6.869415497
9.710286668	7.737611447	10.728645542
7.955806930	9.868599784	3.059072192
8.000528753	9.936602599	6.896059806

Site 4:

11.8719997406	0.0000000000	0.0000000000
0.0000000000	11.8719997406	0.0000000000
0.0000000000	0.0000000000	25.8934001923

Sr Ba Co Fe O

13 14 22 5 80
Cartesian

1.991148052	1.965373941	1.510025419
1.961847957	2.013871060	5.068296473
5.929541502	1.954238005	4.988467121
5.961026046	1.897549207	8.947568010
5.956324734	5.991750781	8.662507567
9.929218215	5.961762110	1.460543131
9.858342377	5.991620189	4.957990589
9.816529194	5.999538813	8.553496353
1.931099478	9.886918280	4.969720299
5.919545279	9.923828327	1.448166086
9.903657800	9.874476424	1.426001335
9.863328616	9.860681161	5.016639140
9.892961128	9.804206058	8.691844790
1.928262070	2.066760819	8.982213379
5.893011359	1.925923286	1.175612156
9.948830759	1.970775701	1.208030693
9.894979368	1.986874133	4.989994831
9.984363654	1.953418837	8.941949142
1.919987286	5.891527359	1.209739657
1.973185717	5.898531839	4.992247557
1.976592981	5.987476861	8.914424458
5.942007102	5.933043742	1.185270394
5.936189822	5.914891455	4.956618238
1.959865333	9.925941543	1.215695139
1.957847093	9.802389642	8.731150971
5.958711006	9.926095879	5.013531932
5.893794911	9.924398183	8.747101306
11.854987165	11.823217694	3.091905024
11.843388221	11.826256926	6.837048747
11.838033949	11.362738440	10.651716250
3.906612107	11.870408893	3.100708780
3.886584043	0.052296159	6.823610072
3.545952627	11.706634656	10.655056499
7.965185810	0.021559552	6.860534061
7.999104113	11.384369223	10.673751534
11.867678333	3.922318762	3.090221953
11.744328255	3.976051433	6.845723036
11.406439271	4.361499649	10.696175218
3.967942857	3.923589066	2.998714676
7.918113331	3.893137387	3.032971645
7.865603476	3.893315467	6.846240904
7.871860020	4.135029382	10.613135084

11.852031037	7.930234643	3.042319162
11.463614822	7.834629429	10.628671124
3.876231659	7.927812755	3.043846873
3.956901898	7.969815890	6.807374911
4.114918214	8.115271631	10.676185513
7.904021267	7.924037459	6.824516341
7.799915702	7.759135382	10.596718668
7.926174419	0.000237440	3.011272975
3.964903625	3.931531434	6.826484240
4.217848452	4.312598882	10.603088445
11.845038429	7.892564788	6.800435479
7.922185427	7.923230163	2.990843083
0.004926880	11.864627229	1.228926667
11.838781885	11.857325949	4.964852340
11.862240957	11.809659870	8.791042406
1.964436053	0.020051808	3.141879286
1.922005526	0.012952352	6.825552077
1.740767578	11.727208832	10.876678111
0.031567647	1.980866901	3.087736186
0.022307488	2.084236402	6.970710479
11.730010624	2.783699011	11.132142397
3.924396362	11.862573373	1.226751621
3.974804873	0.040578495	4.963557670
3.963407753	0.000997248	8.766029381
5.994350749	0.001151584	3.030019797
6.070367163	0.084018142	6.937489247
6.423274228	11.836775517	11.009666615
3.963621449	1.952860853	3.093510414
3.993158985	1.978136341	6.860197447
3.977701641	1.409206369	10.976523062
7.925568947	11.756105279	1.145990106
7.938117651	0.045552863	4.901750123
8.113205903	11.866740445	8.936563315
9.867008936	11.824428638	3.036855655
9.898137320	11.844884093	6.910378857
10.133024835	11.633313186	10.893819542
7.919205555	1.929271190	2.965985418
7.934662899	1.966489909	7.040182472
8.188201325	2.490876138	10.831830742
11.864247325	3.954444394	1.227424849
0.017879232	3.937942314	4.969901553
11.809458046	4.156731397	8.848033780
1.970051509	3.908001131	3.063758898
2.093116658	3.912987371	6.941062536

2.734750756	3.766546254	11.059174796
11.826589342	5.997579933	3.087321892
11.770494143	5.975118109	6.815557225
0.016430848	6.102077275	10.896745496
3.887984939	3.868372395	1.119475264
3.967978473	3.918828394	4.929223021
4.130969158	3.828292524	8.881410373
5.929624606	3.911123467	2.977922276
5.934373406	3.859646476	6.845930183
5.823477057	3.696762639	10.840505031
3.947902922	5.928045630	2.950967246
3.995818313	5.784952418	6.996034224
4.128523526	5.997413725	10.612280602
7.972629554	4.004710440	1.190966942
7.872251796	3.891558411	4.912159270
7.753923575	4.144099589	8.729157179
9.891500872	4.035743848	3.089781765
9.841044873	4.007334152	6.914910202
9.666538349	4.614586939	10.726341030
7.969602194	6.038657052	3.043458472
7.954227954	5.985185565	6.805898987
7.550995483	5.920661247	10.760882826
11.759678751	7.931196275	1.173307643
11.848564413	7.963084466	4.954236046
11.781665695	7.847284981	8.751684438
1.932630966	7.968486226	3.024996477
1.944265526	7.886035188	6.868379761
2.460911210	7.946523026	10.947574241
11.844397341	9.877159496	3.095348846
11.818528254	9.827914441	6.845748930
0.045647839	9.537952720	10.831675382
4.001303177	7.987564529	1.198501921
3.913782794	7.869901140	4.924717569
4.039447912	7.914967251	8.751114783
6.052191132	7.939067411	3.013577488
5.897130943	7.883007828	6.886064954
5.910534431	8.079477551	10.856895553
3.937586154	9.902518088	3.087943333
3.898159243	9.900511720	6.808229393
3.933169770	10.011847333	10.792006693
7.989891441	7.965838770	1.141588228
7.959249810	7.963998610	4.980103552
7.886331988	7.875575956	8.685293759
9.870321224	7.943293842	3.117151089

9.905058696	7.957718322	6.869415497
7.955806930	9.868599784	3.059072192
8.000528753	9.936602599	6.896059806
8.053513488	9.668829645	10.804383738

Site 5:

11.8719997406	0.0000000000	0.0000000000
0.0000000000	11.8719997406	0.0000000000
0.0000000000	0.0000000000	25.8934001923

Sr	Ba	Co	Fe	O
13	14	22	5	80

Cartesian

1.991148052	1.965373941	1.510025419
1.961847957	2.013871060	5.068296473
5.929541502	1.954238005	4.988467121
5.961026046	1.897549207	8.947568010
5.956324734	5.991750781	8.662507567
9.929218215	5.961762110	1.460543131
9.858342377	5.991620189	4.957990589
9.816529194	5.999538813	8.553496353
1.931099478	9.886918280	4.969720299
5.919545279	9.923828327	1.448166086
9.903657800	9.874476424	1.426001335
9.863328616	9.860681161	5.016639140
9.892961128	9.804206058	8.691844790
1.928262070	2.066760819	8.982213379
5.893011359	1.925923286	1.175612156
9.948830759	1.970775701	1.208030693
9.894979368	1.986874133	4.989994831
9.984363654	1.953418837	8.941949142
1.919987286	5.891527359	1.209739657
1.973185717	5.898531839	4.992247557
1.976592981	5.987476861	8.914424458
5.942007102	5.933043742	1.185270394
5.936189822	5.914891455	4.956618238
1.959865333	9.925941543	1.215695139
1.957847093	9.802389642	8.731150971
5.958711006	9.926095879	5.013531932
5.893794911	9.924398183	8.747101306
11.854987165	11.823217694	3.091905024
11.843388221	11.826256926	6.837048747
11.838033949	11.362738440	10.651716250
3.906612107	11.870408893	3.100708780
3.886584043	0.052296159	6.823610072

3.545952627	11.706634656	10.655056499
7.965185810	0.021559552	6.860534061
7.999104113	11.384369223	10.673751534
11.867678333	3.922318762	3.090221953
11.744328255	3.976051433	6.845723036
11.406439271	4.361499649	10.696175218
3.967942857	3.923589066	2.998714676
7.918113331	3.893137387	3.032971645
7.865603476	3.893315467	6.846240904
7.871860020	4.135029382	10.613135084
11.852031037	7.930234643	3.042319162
11.463614822	7.834629429	10.628671124
3.876231659	7.927812755	3.043846873
3.956901898	7.969815890	6.807374911
4.114918214	8.115271631	10.676185513
7.904021267	7.924037459	6.824516341
7.799915702	7.759135382	10.596718668
7.926174419	0.000237440	3.011272975
3.964903625	3.931531434	6.826484240
4.217848452	4.312598882	10.603088445
11.845038429	7.892564788	6.800435479
7.922185427	7.923230163	2.990843083
0.004926880	11.864627229	1.228926667
11.838781885	11.857325949	4.964852340
11.862240957	11.809659870	8.791042406
1.964436053	0.020051808	3.141879286
1.922005526	0.012952352	6.825552077
1.740767578	11.727208832	10.876678111
0.031567647	1.980866901	3.087736186
0.022307488	2.084236402	6.970710479
11.730010624	2.783699011	11.132142397
3.924396362	11.862573373	1.226751621
3.974804873	0.040578495	4.963557670
3.963407753	0.000997248	8.766029381
5.994350749	0.001151584	3.030019797
6.070367163	0.084018142	6.937489247
6.423274228	11.836775517	11.009666615
3.963621449	1.952860853	3.093510414
3.993158985	1.978136341	6.860197447
3.977701641	1.409206369	10.976523062
7.925568947	11.756105279	1.145990106
7.938117651	0.045552863	4.901750123
8.113205903	11.866740445	8.936563315
9.867008936	11.824428638	3.036855655

9.898137320	11.844884093	6.910378857
10.133024835	11.633313186	10.893819542
7.919205555	1.929271190	2.965985418
7.934662899	1.966489909	7.040182472
8.188201325	2.490876138	10.831830742
11.864247325	3.954444394	1.227424849
0.017879232	3.937942314	4.969901553
11.809458046	4.156731397	8.848033780
1.970051509	3.908001131	3.063758898
2.093116658	3.912987371	6.941062536
2.734750756	3.766546254	11.059174796
11.826589342	5.997579933	3.087321892
11.770494143	5.975118109	6.815557225
0.016430848	6.102077275	10.896745496
3.887984939	3.868372395	1.119475264
3.967978473	3.918828394	4.929223021
4.130969158	3.828292524	8.881410373
5.929624606	3.911123467	2.977922276
5.934373406	3.859646476	6.845930183
5.823477057	3.696762639	10.840505031
3.947902922	5.928045630	2.950967246
3.995818313	5.784952418	6.996034224
4.128523526	5.997413725	10.612280602
7.972629554	4.004710440	1.190966942
7.872251796	3.891558411	4.912159270
7.753923575	4.144099589	8.729157179
9.891500872	4.035743848	3.089781765
9.841044873	4.007334152	6.914910202
9.666538349	4.614586939	10.726341030
7.969602194	6.038657052	3.043458472
7.954227954	5.985185565	6.805898987
7.550995483	5.920661247	10.760882826
11.759678751	7.931196275	1.173307643
11.848564413	7.963084466	4.954236046
11.781665695	7.847284981	8.751684438
1.932630966	7.968486226	3.024996477
1.944265526	7.886035188	6.868379761
2.460911210	7.946523026	10.947574241
11.844397341	9.877159496	3.095348846
11.818528254	9.827914441	6.845748930
0.045647839	9.537952720	10.831675382
4.001303177	7.987564529	1.198501921
3.913782794	7.869901140	4.924717569
4.039447912	7.914967251	8.751114783

6.052191132	7.939067411	3.013577488
5.897130943	7.883007828	6.886064954
5.910534431	8.079477551	10.856895553
3.937586154	9.902518088	3.087943333
3.898159243	9.900511720	6.808229393
3.933169770	10.011847333	10.792006693
7.989891441	7.965838770	1.141588228
7.959249810	7.963998610	4.980103552
9.870321224	7.943293842	3.117151089
9.905058696	7.957718322	6.869415497
9.710286668	7.737611447	10.728645542
7.955806930	9.868599784	3.059072192
8.000528753	9.936602599	6.896059806
8.053513488	9.668829645	10.804383738

Atomic coordinates of $BaCo_{0.4}Fe_{0.4}Zr_{0.1}Y_{0.1}O_{3-\delta}$ for oxygen vacancy formation energy calculation:
Site 1:

11.8719997406	0.0000000000	0.0000000000
0.0000000000	11.8719997406	0.0000000000
0.0000000000	0.0000000000	25.8934001923

Ba	Y	Fe	Zr	Co	O
27	3	10	3	11	80

Cartesian

1.963165749	2.000443828	0.822866365
1.905883350	2.027322036	4.941625960
1.909729878	2.018251828	9.085372686
5.993258525	1.973055125	0.923177397
6.028375900	1.961384949	4.906902910
6.024458140	1.957728373	9.092804092
9.851634697	2.006427316	0.938195569
9.863257384	2.010487540	4.979585684
9.850886761	2.013977908	9.026775921
2.000147028	5.950922974	0.922348808
1.921720598	5.989530717	4.894914266
1.908162774	5.930253822	9.004248663
5.975723581	5.956039806	0.918257651
5.980223069	5.988296029	4.922024656
5.978644093	6.014165117	9.043710205
9.824614025	5.949854494	0.978226766
9.889530120	5.961370334	4.939891102
9.875271848	5.939205310	8.965175522
2.038683539	9.868837224	0.943426036
1.964234229	9.819710889	4.917337950
1.954285493	9.883142984	9.020276678

5.969680734	9.864325864	0.985891212
5.974346429	9.844760809	4.918684407
5.911911583	9.774858474	9.008857688
9.822298985	9.876067272	0.958444208
9.870713000	9.845793673	4.954236046
9.901271528	9.821574793	8.962275461
0.006731424	0.022319360	2.839366691
3.956949386	3.958385898	2.779060962
3.970020457	4.008794408	6.993652032
11.835920733	11.865956893	6.914728948
4.002514121	11.811511902	2.813550971
3.995355305	0.122898941	10.960287900
7.896957427	0.022817984	2.905912730
11.830471486	4.092693831	6.946085855
7.948018898	3.934083914	6.853827670
7.913174579	4.065815623	10.869661000
11.853989917	7.863585236	2.656248565
3.847299596	7.913471379	6.891865075
7.912580979	7.868262804	6.922419288
11.786343262	11.853348829	11.051536243
3.967764777	11.842533437	6.919596907
3.945113002	4.040635112	11.190842736
7.956958514	11.733132960	6.979048154
7.871373268	11.531534532	10.814948245
11.785666558	4.001599977	2.752960415
11.787257406	4.025070920	10.943845591
7.991767217	4.044564744	2.851044615
11.802679134	7.958715570	6.973455179
11.854524157	7.836730773	10.875176294
4.035435176	7.951390546	2.827818235
3.672781200	7.909173715	10.898868755
7.849077653	7.913851283	2.885223903
8.025661777	7.833928981	10.882555913
0.009010848	11.863261949	0.649043969
11.869079229	11.866657341	5.078161859
11.866028125	0.006315904	9.095341645
2.175733904	11.833332637	2.810573230
1.877580503	11.839791005	6.968613114
2.176944848	11.823550110	11.132375438
11.816984894	2.172196049	2.821914540
11.847104157	1.782200857	7.002145067
11.836846749	2.112669842	11.076678734
4.036800456	11.789513086	0.927113194
3.953019754	0.005852896	4.818502842

3.955869034	11.813826942	9.051581799
5.958770366	11.810063518	2.895762517
6.058851324	11.870195197	6.933760597
5.936094846	11.803118398	10.991618915
4.016321256	1.767147161	2.768884856
3.942382442	1.916176374	7.056857821
4.029689128	1.911332598	11.255886957
7.876751284	11.834875997	0.996248572
7.926625555	11.861279325	4.827643212
7.927919603	11.871358653	8.953186878
9.718573324	11.825247806	2.861013574
9.861262889	0.002706816	6.965997880
9.725945835	0.004903136	11.092888003
7.919383635	1.881296439	2.863085046
7.946439922	2.115732818	6.958100393
7.973021330	2.336599501	11.048662075
11.789109438	4.018968712	0.896843809
11.838390109	3.935366090	5.097115828
11.832418493	3.958172202	8.872813764
1.744519130	3.996055753	2.787217383
1.775528793	3.937040042	6.984770595
1.847663064	4.040599496	11.138175560
11.846000061	6.001438333	2.906093984
11.855283965	5.920364447	6.969830103
11.844207389	5.987073213	10.949956434
3.933229130	3.926212778	0.558261708
3.938120394	3.951120234	4.896933951
3.981583785	3.993610121	9.259790630
6.164725817	3.967135561	2.802080195
6.140305114	3.991686857	6.970373865
6.099774107	4.084537767	11.164172533
3.956818794	6.129204794	2.790946033
3.987507913	6.152794458	6.971124773
3.982201129	6.141326106	11.185482802
7.987232113	3.981192009	0.908340479
7.930662035	3.978556425	4.888855210
7.884313748	4.026246248	9.047723682
9.878809704	4.009839144	2.893458004
9.881243464	3.961080841	6.964884464
9.934596231	4.085297575	11.051277309
7.922363507	6.018771452	2.877300523
7.941881074	5.958212382	6.917887943
11.854464797	7.873854516	0.852100014
0.016324000	7.933143283	5.198876891

11.849371709	7.906051379	8.847153404
1.863132279	7.902050515	2.876316574
2.021932148	7.925141555	6.978918687
1.882198711	7.865924020	11.005109376
0.006577088	9.693060396	2.841981925
11.845608285	9.994597318	7.003336163
11.832335389	9.769136171	11.071733095
3.972335497	7.960365778	0.937703595
3.955726570	7.910455891	4.964489832
3.947914794	7.881120180	8.961136152
5.908266879	7.914243059	2.860340346
6.076730555	7.919597331	6.923739851
6.320557686	7.879731156	11.067046389
3.989680489	9.894278920	2.891490106
3.971789385	9.753951883	6.948131434
3.981951817	9.576607951	11.113110748
7.913578227	7.921318771	0.956087909
7.928062067	7.914824787	4.901465296
7.948054514	7.919015603	8.856397348
10.021060005	7.904531763	2.897316121
9.827273353	7.920998227	6.936660658
9.839335305	7.891389460	11.003245051
7.909280563	9.798080106	2.887269482
7.932062931	9.850020105	6.946137642
7.946831698	9.763152683	10.964586205

Site 2:

11.8719997406	0.0000000000	0.0000000000
0.0000000000	11.8719997406	0.0000000000
0.0000000000	0.0000000000	25.8934001923

Ba	Y	Fe	Zr	Co	O
27	3	10	3	11	81

Cartesian

1.963165749	2.000443828	0.822866365
1.905883350	2.027322036	4.941625960
1.909729878	2.018251828	9.085372686
5.993258525	1.973055125	0.923177397
6.028375900	1.961384949	4.906902910
6.024458140	1.957728373	9.092804092
9.851634697	2.006427316	0.938195569
9.863257384	2.010487540	4.979585684
9.850886761	2.013977908	9.026775921
2.000147028	5.950922974	0.922348808
1.921720598	5.989530717	4.894914266

1.908162774	5.930253822	9.004248663
5.975723581	5.956039806	0.918257651
5.980223069	5.988296029	4.922024656
5.978644093	6.014165117	9.043710205
9.824614025	5.949854494	0.978226766
9.889530120	5.961370334	4.939891102
9.875271848	5.939205310	8.965175522
2.038683539	9.868837224	0.943426036
1.964234229	9.819710889	4.917337950
1.954285493	9.883142984	9.020276678
5.969680734	9.864325864	0.985891212
5.974346429	9.844760809	4.918684407
5.911911583	9.774858474	9.008857688
9.822298985	9.876067272	0.958444208
9.870713000	9.845793673	4.954236046
9.901271528	9.821574793	8.962275461
0.006731424	0.022319360	2.839366691
3.956949386	3.958385898	2.779060962
3.970020457	4.008794408	6.993652032
11.835920733	11.865956893	6.914728948
4.002514121	11.811511902	2.813550971
3.995355305	0.122898941	10.960287900
7.896957427	0.022817984	2.905912730
11.830471486	4.092693831	6.946085855
7.948018898	3.934083914	6.853827670
7.913174579	4.065815623	10.869661000
11.853989917	7.863585236	2.656248565
3.847299596	7.913471379	6.891865075
7.912580979	7.868262804	6.922419288
11.786343262	11.853348829	11.051536243
3.967764777	11.842533437	6.919596907
3.945113002	4.040635112	11.190842736
7.956958514	11.733132960	6.979048154
7.871373268	11.531534532	10.814948245
11.785666558	4.001599977	2.752960415
11.787257406	4.025070920	10.943845591
7.991767217	4.044564744	2.851044615
11.802679134	7.958715570	6.973455179
11.854524157	7.836730773	10.875176294
4.035435176	7.951390546	2.827818235
3.672781200	7.909173715	10.898868755
7.849077653	7.913851283	2.885223903
8.025661777	7.833928981	10.882555913
0.009010848	11.863261949	0.649043969

11.869079229	11.866657341	5.078161859
11.866028125	0.006315904	9.095341645
2.175733904	11.833332637	2.810573230
1.877580503	11.839791005	6.968613114
2.176944848	11.823550110	11.132375438
11.816984894	2.172196049	2.821914540
11.847104157	1.782200857	7.002145067
11.836846749	2.112669842	11.076678734
4.036800456	11.789513086	0.927113194
3.953019754	0.005852896	4.818502842
3.955869034	11.813826942	9.051581799
5.958770366	11.810063518	2.895762517
6.058851324	11.870195197	6.933760597
5.936094846	11.803118398	10.991618915
4.016321256	1.767147161	2.768884856
3.942382442	1.916176374	7.056857821
4.029689128	1.911332598	11.255886957
7.876751284	11.834875997	0.996248572
7.926625555	11.861279325	4.827643212
7.927919603	11.871358653	8.953186878
9.718573324	11.825247806	2.861013574
9.861262889	0.002706816	6.965997880
9.725945835	0.004903136	11.092888003
7.919383635	1.881296439	2.863085046
7.946439922	2.115732818	6.958100393
7.973021330	2.336599501	11.048662075
11.789109438	4.018968712	0.896843809
11.838390109	3.935366090	5.097115828
11.832418493	3.958172202	8.872813764
1.744519130	3.996055753	2.787217383
1.775528793	3.937040042	6.984770595
1.847663064	4.040599496	11.138175560
11.846000061	6.001438333	2.906093984
11.855283965	5.920364447	6.969830103
11.844207389	5.987073213	10.949956434
3.933229130	3.926212778	0.558261708
3.938120394	3.951120234	4.896933951
3.981583785	3.993610121	9.259790630
6.164725817	3.967135561	2.802080195
6.140305114	3.991686857	6.970373865
6.099774107	4.084537767	11.164172533
3.956818794	6.129204794	2.790946033
3.987507913	6.152794458	6.971124773
3.982201129	6.141326106	11.185482802

7.987232113	3.981192009	0.908340479
7.930662035	3.978556425	4.888855210
7.884313748	4.026246248	9.047723682
9.878809704	4.009839144	2.893458004
9.881243464	3.961080841	6.964884464
9.934596231	4.085297575	11.051277309
7.922363507	6.018771452	2.877300523
7.941881074	5.958212382	6.917887943
7.969483474	5.943645438	10.996176153
11.854464797	7.873854516	0.852100014
0.016324000	7.933143283	5.198876891
11.849371709	7.906051379	8.847153404
1.863132279	7.902050515	2.876316574
2.021932148	7.925141555	6.978918687
1.882198711	7.865924020	11.005109376
0.006577088	9.693060396	2.841981925
11.845608285	9.994597318	7.003336163
11.832335389	9.769136171	11.071733095
3.972335497	7.960365778	0.937703595
3.955726570	7.910455891	4.964489832
3.947914794	7.881120180	8.961136152
5.908266879	7.914243059	2.860340346
6.076730555	7.919597331	6.923739851
3.989680489	9.894278920	2.891490106
3.971789385	9.753951883	6.948131434
3.981951817	9.576607951	11.113110748
7.913578227	7.921318771	0.956087909
7.928062067	7.914824787	4.901465296
7.948054514	7.919015603	8.856397348
10.021060005	7.904531763	2.897316121
9.827273353	7.920998227	6.936660658
9.839335305	7.891389460	11.003245051
7.909280563	9.798080106	2.887269482
7.932062931	9.850020105	6.946137642
7.946831698	9.763152683	10.964586205

Site 3:

11.8719997406	0.0000000000	0.0000000000
0.0000000000	11.8719997406	0.0000000000
0.0000000000	0.0000000000	25.8934001923

Ba	Y	Fe	Zr	Co	O
27	3	10	3	11	80

Cartesian

1.963165749	2.000443828	0.822866365

1.905883350	2.027322036	4.941625960
1.909729878	2.018251828	9.085372686
5.993258525	1.973055125	0.923177397
6.028375900	1.961384949	4.906902910
6.024458140	1.957728373	9.092804092
9.851634697	2.006427316	0.938195569
9.863257384	2.010487540	4.979585684
9.850886761	2.013977908	9.026775921
2.000147028	5.950922974	0.922348808
1.921720598	5.989530717	4.894914266
1.908162774	5.930253822	9.004248663
5.975723581	5.956039806	0.918257651
5.980223069	5.988296029	4.922024656
5.978644093	6.014165117	9.043710205
9.824614025	5.949854494	0.978226766
9.889530120	5.961370334	4.939891102
9.875271848	5.939205310	8.965175522
2.038683539	9.868837224	0.943426036
1.964234229	9.819710889	4.917337950
1.954285493	9.883142984	9.020276678
5.969680734	9.864325864	0.985891212
5.974346429	9.844760809	4.918684407
5.911911583	9.774858474	9.008857688
9.822298985	9.876067272	0.958444208
9.870713000	9.845793673	4.954236046
9.901271528	9.821574793	8.962275461
0.006731424	0.022319360	2.839366691
3.956949386	3.958385898	2.779060962
3.970020457	4.008794408	6.993652032
11.835920733	11.865956893	6.914728948
4.002514121	11.811511902	2.813550971
3.995355305	0.122898941	10.960287900
7.896957427	0.022817984	2.905912730
11.830471486	4.092693831	6.946085855
7.948018898	3.934083914	6.853827670
7.913174579	4.065815623	10.869661000
11.853989917	7.863585236	2.656248565
3.847299596	7.913471379	6.891865075
7.912580979	7.868262804	6.922419288
11.786343262	11.853348829	11.051536243
3.967764777	11.842533437	6.919596907
3.945113002	4.040635112	11.190842736
7.956958514	11.733132960	6.979048154
7.871373268	11.531534532	10.814948245

11.785666558	4.001599977	2.752960415
11.787257406	4.025070920	10.943845591
7.991767217	4.044564744	2.851044615
11.802679134	7.958715570	6.973455179
11.854524157	7.836730773	10.875176294
4.035435176	7.951390546	2.827818235
3.672781200	7.909173715	10.898868755
7.849077653	7.913851283	2.885223903
8.025661777	7.833928981	10.882555913
0.009010848	11.863261949	0.649043969
11.869079229	11.866657341	5.078161859
11.866028125	0.006315904	9.095341645
2.175733904	11.833332637	2.810573230
1.877580503	11.839791005	6.968613114
2.176944848	11.823550110	11.132375438
11.816984894	2.172196049	2.821914540
11.847104157	1.782200857	7.002145067
11.836846749	2.112669842	11.076678734
4.036800456	11.789513086	0.927113194
3.953019754	0.005852896	4.818502842
3.955869034	11.813826942	9.051581799
5.958770366	11.810063518	2.895762517
6.058851324	11.870195197	6.933760597
5.936094846	11.803118398	10.991618915
4.016321256	1.767147161	2.768884856
3.942382442	1.916176374	7.056857821
4.029689128	1.911332598	11.255886957
7.876751284	11.834875997	0.996248572
7.926625555	11.861279325	4.827643212
7.927919603	11.871358653	8.953186878
9.718573324	11.825247806	2.861013574
9.861262889	0.002706816	6.965997880
9.725945835	0.004903136	11.092888003
7.919383635	1.881296439	2.863085046
7.946439922	2.115732818	6.958100393
7.973021330	2.336599501	11.048662075
11.789109438	4.018968712	0.896843809
11.838390109	3.935366090	5.097115828
11.832418493	3.958172202	8.872813764
1.744519130	3.996055753	2.787217383
1.775528793	3.937040042	6.984770595
1.847663064	4.040599496	11.138175560
11.846000061	6.001438333	2.906093984
11.855283965	5.920364447	6.969830103

11.844207389	5.987073213	10.949956434
3.933229130	3.926212778	0.558261708
3.938120394	3.951120234	4.896933951
3.981583785	3.993610121	9.259790630
6.164725817	3.967135561	2.802080195
6.140305114	3.991686857	6.970373865
6.099774107	4.084537767	11.164172533
3.956818794	6.129204794	2.790946033
3.987507913	6.152794458	6.971124773
3.982201129	6.141326106	11.185482802
7.987232113	3.981192009	0.908340479
7.930662035	3.978556425	4.888855210
7.884313748	4.026246248	9.047723682
9.878809704	4.009839144	2.893458004
9.881243464	3.961080841	6.964884464
9.934596231	4.085297575	11.051277309
7.922363507	6.018771452	2.877300523
7.941881074	5.958212382	6.917887943
7.969483474	5.943645438	10.996176153
11.854464797	7.873854516	0.852100014
0.016324000	7.933143283	5.198876891
11.849371709	7.906051379	8.847153404
1.863132279	7.902050515	2.876316574
2.021932148	7.925141555	6.978918687
1.882198711	7.865924020	11.005109376
0.006577088	9.693060396	2.841981925
11.845608285	9.994597318	7.003336163
11.832335389	9.769136171	11.071733095
3.972335497	7.960365778	0.937703595
3.955726570	7.910455891	4.964489832
3.947914794	7.881120180	8.961136152
5.908266879	7.914243059	2.860340346
6.076730555	7.919597331	6.923739851
6.320557686	7.879731156	11.067046389
3.989680489	9.894278920	2.891490106
3.971789385	9.753951883	6.948131434
3.981951817	9.576607951	11.113110748
7.913578227	7.921318771	0.956087909
7.928062067	7.914824787	4.901465296
7.948054514	7.919015603	8.856397348
10.021060005	7.904531763	2.897316121
9.827273353	7.920998227	6.936660658
9.839335305	7.891389460	11.003245051
7.909280563	9.798080106	2.887269482

7.932062931	9.850020105	6.946137642

Site 4:

11.8719997406	0.0000000000	0.0000000000
0.0000000000	11.8719997406	0.0000000000
0.0000000000	0.0000000000	25.8934001923

Ba	Y	Fe	Zr	Co	O
27	3	10	3	11	81

Cartesian

1.963165749	2.000443828	0.822866365
1.905883350	2.027322036	4.941625960
1.909729878	2.018251828	9.085372686
5.993258525	1.973055125	0.923177397
6.028375900	1.961384949	4.906902910
6.024458140	1.957728373	9.092804092
9.851634697	2.006427316	0.938195569
9.863257384	2.010487540	4.979585684
9.850886761	2.013977908	9.026775921
2.000147028	5.950922974	0.922348808
1.921720598	5.989530717	4.894914266
1.908162774	5.930253822	9.004248663
5.975723581	5.956039806	0.918257651
5.980223069	5.988296029	4.922024656
5.978644093	6.014165117	9.043710205
9.824614025	5.949854494	0.978226766
9.889530120	5.961370334	4.939891102
9.875271848	5.939205310	8.965175522
2.038683539	9.868837224	0.943426036
1.964234229	9.819710889	4.917337950
1.954285493	9.883142984	9.020276678
5.969680734	9.864325864	0.985891212
5.974346429	9.844760809	4.918684407
5.911911583	9.774858474	9.008857688
9.822298985	9.876067272	0.958444208
9.870713000	9.845793673	4.954236046
9.901271528	9.821574793	8.962275461
0.006731424	0.022319360	2.839366691
3.956949386	3.958385898	2.779060962
3.970020457	4.008794408	6.993652032
11.835920733	11.865956893	6.914728948
4.002514121	11.811511902	2.813550971
3.995355305	0.122898941	10.960287900
7.896957427	0.022817984	2.905912730
11.830471486	4.092693831	6.946085855

7.948018898	3.934083914	6.853827670
7.913174579	4.065815623	10.869661000
11.853989917	7.863585236	2.656248565
3.847299596	7.913471379	6.891865075
7.912580979	7.868262804	6.922419288
11.786343262	11.853348829	11.051536243
3.967764777	11.842533437	6.919596907
3.945113002	4.040635112	11.190842736
7.956958514	11.733132960	6.979048154
7.871373268	11.531534532	10.814948245
11.785666558	4.001599977	2.752960415
11.787257406	4.025070920	10.943845591
7.991767217	4.044564744	2.851044615
11.802679134	7.958715570	6.973455179
11.854524157	7.836730773	10.875176294
4.035435176	7.951390546	2.827818235
3.672781200	7.909173715	10.898868755
7.849077653	7.913851283	2.885223903
8.025661777	7.833928981	10.882555913
0.009010848	11.863261949	0.649043969
11.869079229	11.866657341	5.078161859
11.866028125	0.006315904	9.095341645
2.175733904	11.833332637	2.810573230
1.877580503	11.839791005	6.968613114
2.176944848	11.823550110	11.132375438
11.816984894	2.172196049	2.821914540
11.847104157	1.782200857	7.002145067
11.836846749	2.112669842	11.076678734
4.036800456	11.789513086	0.927113194
3.953019754	0.005852896	4.818502842
3.955869034	11.813826942	9.051581799
5.958770366	11.810063518	2.895762517
6.058851324	11.870195197	6.933760597
5.936094846	11.803118398	10.991618915
4.016321256	1.767147161	2.768884856
3.942382442	1.916176374	7.056857821
4.029689128	1.911332598	11.255886957
7.876751284	11.834875997	0.996248572
7.926625555	11.861279325	4.827643212
7.927919603	11.871358653	8.953186878
9.718573324	11.825247806	2.861013574
9.861262889	0.002706816	6.965997880
9.725945835	0.004903136	11.092888003
7.919383635	1.881296439	2.863085046

7.946439922	2.115732818	6.958100393
7.973021330	2.336599501	11.048662075
11.789109438	4.018968712	0.896843809
11.838390109	3.935366090	5.097115828
11.832418493	3.958172202	8.872813764
1.744519130	3.996055753	2.787217383
1.775528793	3.937040042	6.984770595
1.847663064	4.040599496	11.138175560
11.846000061	6.001438333	2.906093984
11.855283965	5.920364447	6.969830103
11.844207389	5.987073213	10.949956434
3.933229130	3.926212778	0.558261708
3.938120394	3.951120234	4.896933951
3.981583785	3.993610121	9.259790630
6.164725817	3.967135561	2.802080195
6.140305114	3.991686857	6.970373865
6.099774107	4.084537767	11.164172533
3.956818794	6.129204794	2.790946033
3.987507913	6.152794458	6.971124773
3.982201129	6.141326106	11.185482802
7.987232113	3.981192009	0.908340479
7.930662035	3.978556425	4.888855210
7.884313748	4.026246248	9.047723682
9.878809704	4.009839144	2.893458004
9.881243464	3.961080841	6.964884464
9.934596231	4.085297575	11.051277309
7.922363507	6.018771452	2.877300523
7.941881074	5.958212382	6.917887943
7.969483474	5.943645438	10.996176153
11.854464797	7.873854516	0.852100014
0.016324000	7.933143283	5.198876891
11.849371709	7.906051379	8.847153404
1.863132279	7.902050515	2.876316574
2.021932148	7.925141555	6.978918687
1.882198711	7.865924020	11.005109376
0.006577088	9.693060396	2.841981925
11.845608285	9.994597318	7.003336163
11.832335389	9.769136171	11.071733095
3.972335497	7.960365778	0.937703595
3.955726570	7.910455891	4.964489832
3.947914794	7.881120180	8.961136152
5.908266879	7.914243059	2.860340346
6.076730555	7.919597331	6.923739851
6.320557686	7.879731156	11.067046389

3.989680489	9.894278920	2.891490106
3.971789385	9.753951883	6.948131434
3.981951817	9.576607951	11.113110748
7.913578227	7.921318771	0.956087909
7.928062067	7.914824787	4.901465296
7.948054514	7.919015603	8.856397348
10.021060005	7.904531763	2.897316121
9.827273353	7.920998227	6.936660658
7.909280563	9.798080106	2.887269482
7.932062931	9.850020105	6.946137642
7.946831698	9.763152683	10.964586205

Site 5:

11.8719997406	0.0000000000	0.0000000000
0.0000000000	11.8719997406	0.0000000000
0.0000000000	0.0000000000	25.8934001923

Ba Y Fe Zr Co O

27 3 10 3 11 80

Cartesian

1.963165749	2.000443828	0.822866365
1.905883350	2.027322036	4.941625960
1.909729878	2.018251828	9.085372686
5.993258525	1.973055125	0.923177397
6.028375900	1.961384949	4.906902910
6.024458140	1.957728373	9.092804092
9.851634697	2.006427316	0.938195569
9.863257384	2.010487540	4.979585684
9.850886761	2.013977908	9.026775921
2.000147028	5.950922974	0.922348808
1.921720598	5.989530717	4.894914266
1.908162774	5.930253822	9.004248663
5.975723581	5.956039806	0.918257651
5.980223069	5.988296029	4.922024656
5.978644093	6.014165117	9.043710205
9.824614025	5.949854494	0.978226766
9.889530120	5.961370334	4.939891102
9.875271848	5.939205310	8.965175522
2.038683539	9.868837224	0.943426036
1.964234229	9.819710889	4.917337950
1.954285493	9.883142984	9.020276678
5.969680734	9.864325864	0.985891212
5.974346429	9.844760809	4.918684407
5.911911583	9.774858474	9.008857688
9.822298985	9.876067272	0.958444208

9.870713000	9.845793673	4.954236046
9.901271528	9.821574793	8.962275461
0.006731424	0.022319360	2.839366691
3.956949386	3.958385898	2.779060962
3.970020457	4.008794408	6.993652032
11.835920733	11.865956893	6.914728948
4.002514121	11.811511902	2.813550971
3.995355305	0.122898941	10.960287900
7.896957427	0.022817984	2.905912730
11.830471486	4.092693831	6.946085855
7.948018898	3.934083914	6.853827670
7.913174579	4.065815623	10.869661000
11.853989917	7.863585236	2.656248565
3.847299596	7.913471379	6.891865075
7.912580979	7.868262804	6.922419288
11.786343262	11.853348829	11.051536243
3.967764777	11.842533437	6.919596907
3.945113002	4.040635112	11.190842736
7.956958514	11.733132960	6.979048154
7.871373268	11.531534532	10.814948245
11.785666558	4.001599977	2.752960415
11.787257406	4.025070920	10.943845591
7.991767217	4.044564744	2.851044615
11.802679134	7.958715570	6.973455179
11.854524157	7.836730773	10.875176294
4.035435176	7.951390546	2.827818235
3.672781200	7.909173715	10.898868755
7.849077653	7.913851283	2.885223903
8.025661777	7.833928981	10.882555913
0.009010848	11.863261949	0.649043969
11.869079229	11.866657341	5.078161859
11.866028125	0.006315904	9.095341645
2.175733904	11.833332637	2.810573230
1.877580503	11.839791005	6.968613114
2.176944848	11.823550110	11.132375438
11.816984894	2.172196049	2.821914540
11.847104157	1.782200857	7.002145067
11.836846749	2.112669842	11.076678734
4.036800456	11.789513086	0.927113194
3.953019754	0.005852896	4.818502842
3.955869034	11.813826942	9.051581799
5.958770366	11.810063518	2.895762517
6.058851324	11.870195197	6.933760597
5.936094846	11.803118398	10.991618915

4.016321256	1.767147161	2.768884856
3.942382442	1.916176374	7.056857821
4.029689128	1.911332598	11.255886957
7.876751284	11.834875997	0.996248572
7.926625555	11.861279325	4.827643212
7.927919603	11.871358653	8.953186878
9.718573324	11.825247806	2.861013574
9.861262889	0.002706816	6.965997880
9.725945835	0.004903136	11.092888003
7.919383635	1.881296439	2.863085046
7.946439922	2.115732818	6.958100393
7.973021330	2.336599501	11.048662075
11.789109438	4.018968712	0.896843809
11.838390109	3.935366090	5.097115828
11.832418493	3.958172202	8.872813764
1.744519130	3.996055753	2.787217383
1.775528793	3.937040042	6.984770595
1.847663064	4.040599496	11.138175560
11.846000061	6.001438333	2.906093984
11.855283965	5.920364447	6.969830103
11.844207389	5.987073213	10.949956434
3.933229130	3.926212778	0.558261708
3.938120394	3.951120234	4.896933951
3.981583785	3.993610121	9.259790630
6.164725817	3.967135561	2.802080195
6.140305114	3.991686857	6.970373865
6.099774107	4.084537767	11.164172533
3.956818794	6.129204794	2.790946033
3.987507913	6.152794458	6.971124773
3.982201129	6.141326106	11.185482802
7.987232113	3.981192009	0.908340479
7.930662035	3.978556425	4.888855210
7.884313748	4.026246248	9.047723682
9.878809704	4.009839144	2.893458004
9.881243464	3.961080841	6.964884464
9.934596231	4.085297575	11.051277309
7.922363507	6.018771452	2.877300523
7.941881074	5.958212382	6.917887943
7.969483474	5.943645438	10.996176153
11.854464797	7.873854516	0.852100014
0.016324000	7.933143283	5.198876891
11.849371709	7.906051379	8.847153404
1.863132279	7.902050515	2.876316574
2.021932148	7.925141555	6.978918687

1.882198711	7.865924020	11.005109376
0.006577088	9.693060396	2.841981925
11.845608285	9.994597318	7.003336163
11.832335389	9.769136171	11.071733095
3.972335497	7.960365778	0.937703595
3.955726570	7.910455891	4.964489832
3.947914794	7.881120180	8.961136152
5.908266879	7.914243059	2.860340346
6.076730555	7.919597331	6.923739851
6.320557686	7.879731156	11.067046389
3.989680489	9.894278920	2.891490106
3.971789385	9.753951883	6.948131434
3.981951817	9.576607951	11.113110748
7.913578227	7.921318771	0.956087909
7.928062067	7.914824787	4.901465296
10.021060005	7.904531763	2.897316121
9.827273353	7.920998227	6.936660658
9.839335305	7.891389460	11.003245051
7.909280563	9.798080106	2.887269482
7.932062931	9.850020105	6.946137642
7.946831698	9.763152683	10.964586205
